# Usual suspects meet mission impossible: Nutrient losses and effects of mitigation measures on a coastal catchment in the Baltic Sea region

**DOI:** 10.1007/s13280-025-02132-w

**Published:** 2025-02-03

**Authors:** Faruk Djodjic, Oksana Golovko, Linda Kumblad, Emil Rydin, Sara Sandström, Elin Widén-Nilsson

**Affiliations:** 1 Department of Aquatic Sciences and Assessment, Swedish University of Agricultural Sciences, Lennart Hjälmsv. 9, P.O. Box 7050, 750 07 Uppsala, Sweden; 2 Baltic Sea Center, Stockholm University, 106 91 Stockholm, Sweden; 3 Department of Soil and Environment, Swedish University of Agricultural Sciences, Box 7014, 750 07 Uppsala, Sweden

**Keywords:** Agriculture, Eutrophication, Mitigation, Nitrogen, On-site wastewater treatment, Phosphorus

## Abstract

Eutrophication of coastal areas is a global problem. A full-scale coastal remediation project was initiated in Björnöfjärden bay in the Stockholm archipelago in 2011. Measures to reduce external nutrient inputs from the surrounding catchment (15 km^2^) targeted agriculture, on-site wastewater treatment facilities, and horse keeping. The effects were evaluated at 22 water quality monitoring stations over 11 years (2012–2022) to determine temporal trends in nutrient concentrations, spatial correlations within and between monitored sub-catchments, and effects of individual mitigation measures at local and catchment scale. The effect of individual measures varied from no significant effect to significant nutrient decreases (21% reduction in dissolved P concentrations in one lime filter) or increases (11% higher concentrations in total P in one constructed wetland). However, few significant trends were detected at sub-catchment outlet stations. Tailored placement, design, dimensioning, and maintenance of implemented mitigation measures are needed to improve their nutrient retention effect.

**Supplementary Information:**

The online version contains supplementary material available at 10.1007/s13280-025-02132-w.

## Introduction

Eutrophication of inland, coastal, and marine waters remains an important environmental issue. The brackish Baltic Sea is one of the most polluted seas in the world. At least 86% of coastal waters and 97% of the open Baltic Sea are below good eutrophication status (HELCOM 2018). Nutrient loads to the Baltic Sea started to increase with the introduction of water toilets and large-scale application of mineral fertilizers in the early 1950s, and peaked in the 1980s (Gustafsson et al. 2012). After remediation of major point sources in general, and wastewater treatment plant sources in particular, during 1970s (Persson 2001), diffuse losses from agriculture and emissions from local on-site wastewater treatment (OWT) facilities are now the main external phosphorus (P) sources to water recipients. According to Hansson et al. (2019), agriculture contributes 50% of the total anthropogenic load of P to the Baltic Proper and OWTs contribute 12.5%. Unlike much of the nitrogen (N) entering the Baltic Sea, which might be removed from the system through denitrification, most of the P accumulates in sediment and the water column, where P recycling from the sediments occurs, especially under anoxic conditions (Rydin et al. 2017). Therefore, in many Baltic coastal areas and bays with limited water exchange with adjacent basins, mitigation strategies for both internal and external P sources are needed. Addressing external sources requires multiple mitigation measures, ranging from restoration and improvement of OWTs (Envall et al. 2023) to counteracting diffuse losses from arable land at farm (e.g., sustainable nutrient management), field (e.g., tillage, crop rotation, improving soil structure with structure liming), and catchment scale (e.g., grassed waterways and vegetated buffer strips), and in aquatic ecosystems (e.g., applying chemicals to bind P, restore flood plains and wetlands) (Schoumans et al. 2014). Practical implementation of mitigation measures to reduce nutrient losses is increasing, but there are few documented positive effects at catchment scale (Tomczyk et al. 2023). Detailed follow-up studies with systematic monitoring of effects of voluntary measures before, during, and after implementation are important, resulting in management-relevant information regarding measurement efficiency or their effects on nutrient concentrations in recipient waters (Melland et al. 2018; Bieroza et al. 2019; Sandström et al. 2024).

In 2011, a full-scale coastal remediation project addressing both internal and external nutrient sources was initiated in Björnöfjärden bay in the Stockholm archipelago in the Baltic Sea. Internal sediment P recycling was inhibited by injection of dissolved Al into bottom sediment (Rydin et al. 2017; Rydin and Kumblad 2019). In parallel, measures to reduce external sources from agriculture, private sewers, and horse keeping (estimated to be the greatest nutrient sources from the catchment to the bay) were introduced in the 15 km^2^ catchment surrounding Björnöfjärden. Measures aiming at reducing diffuse nutrient sources from arable land and horse paddocks included structure liming (SL), constructed wetlands (CW, *n* = 3), lime filters (LF, *n* = 3), and lime filter drainage systems (LFDS, *n* = 2). Structure liming involves the addition of highly reactive lime materials to improve the structure of clay soils (Blomquist 2021), and thereby decrease P losses (Ulén and Etana 2014; Norberg and Aronsson 2022). Restoration and creation of new wetlands is a commonly used nature-based solution to retain nutrients and counteract eutrophication (Johnston 1991; Hambäck et al. 2023). The efficiency of P removal in CW can be further increased by installing a porous filter with high P retention capacity (in the present case lime filters) at the outlet of the wetland (Ballantine and Tanner 2010). The functioning of tile drainage systems can be further improved at installation or renovation by adding quicklime (CaO) to drain backfill, to reduce surface runoff and associated erosion and P losses (Ulén et al. 2018). Värmdö municipality, where the study catchment is situated, has higher health and environment protection requirements due to sensitive conditions such as thin soil cover, proximity to the coast, and dense settlements (Värmdö municipality 2022), and thus, there is a requirement for removal of at least 50% N and 90% P in effluent from OWTs (Swedish Agency for Marine and Water Management 2016). Consultations and a monetary subsidies were offered to property owners to improve their OWTs and thereby reduce nutrient emissions to the bay.

To monitor the effects of implemented measures, an extensive monitoring program was conducted over 11 years (2012–2022). The aims of this study were to (i) investigate temporal trends in nutrient concentrations, water discharge, and loads, (ii) explore spatial correlations within and between monitored sub-catchments and land use, and (iii) quantify the effects of individual mitigation measures at local and catchment scale.

## Materials and methods

### Study site and water quality monitoring program

**Fig. 1:**
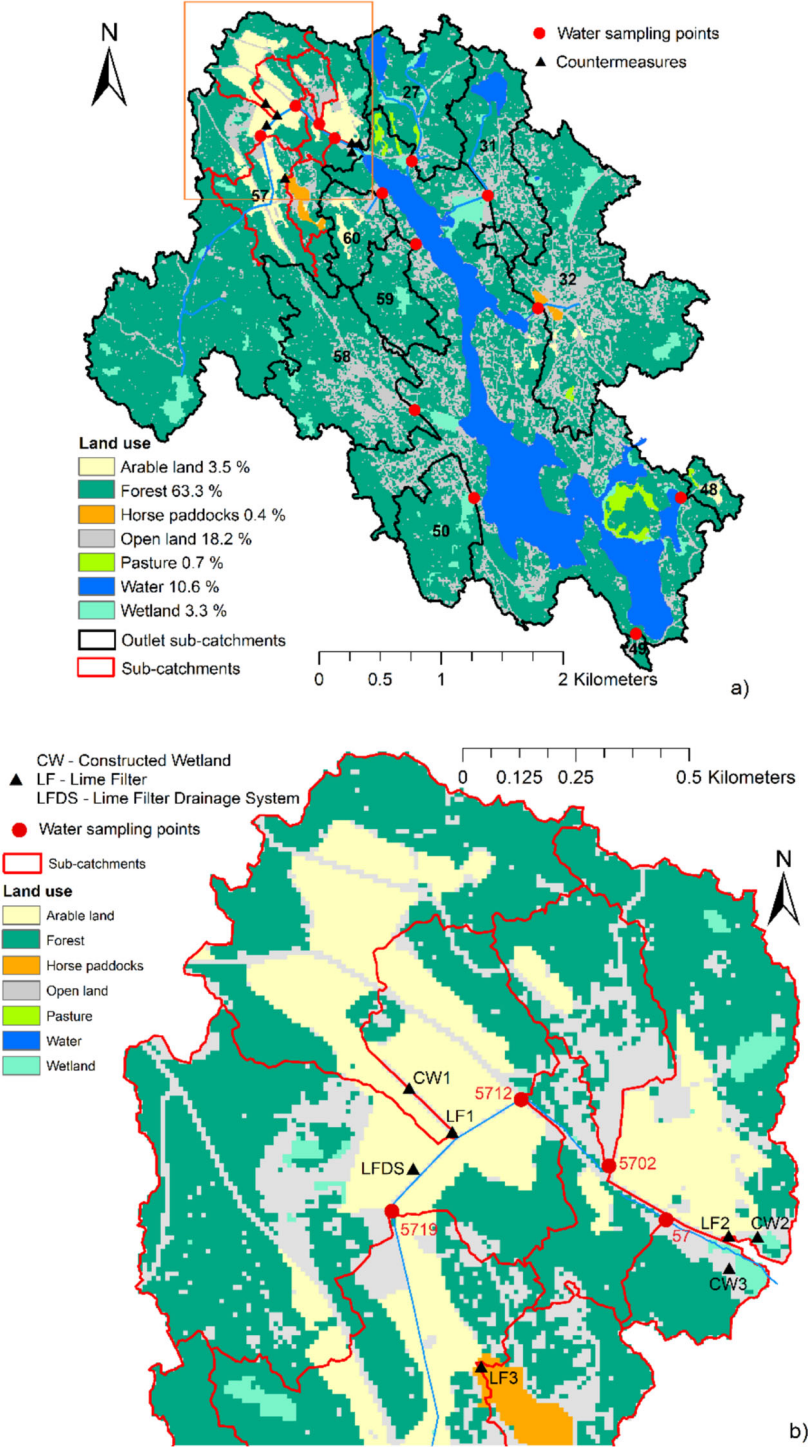
Land use, outlet sub-catchments (in black), other sub-catchments (in red), water quality sampling points (red circle), and location of implemented mitigation measures (black triangles) to reduce nutrient losses from diffuse sources in **a** the whole catchment and **b** sub-catchment 57, where most mitigation measures were implemented

The semi-enclosed bay Björnöfjärden (surface area 1.5 km^2^) is located 34 km east of Stockholm city, in the Stockholm archipelago (Rydin et al. 2017; Rydin and Kumblad 2019), and is surrounded by a 15.4 km^2^ headwater catchment. The catchment is dominated by forest (63.3%), followed by open land (18.2%) (Fig. 1). The proportion of arable land is now low (3.5%) and is concentrated in the northern part of the catchment. However, historical maps show a much larger proportion of arable land than today, before conversion to forest or urban development, with the proportion in the whole of Värmdö municipality decreasing by 72% between 1951 and 2020 (Statistics Sweden 2024a). Therefore, in this study former arable land was digitized from the district economic map of Sweden for the period 1859–1934 (Swedish Environmental Protection Agency 2024), to assess whether legacy nutrients from that land are still influencing nutrient concentrations.

During the monitoring period (2012–2022), monthly grab samples were collected at 65 different stations. Water samples from different stations were collected within the same day and thereby during similar hydrological conditions. However, the number of water samples collected at some stations was low due to occasionally low or no water flow, low relevance, and high sampling costs. Two sets of sampling stations were included in this study to secure stations with high number of sampling occasions and to facilitate analyses. Firstly, 10 outlet sub-catchments flowing directly into the bay (indicated in Fig. 1) were selected to illustrate nutrient pressure on the bay. Secondly, 13 stations within sub-catchment 57, where most mitigation measures were implemented (Fig. 1), were selected to capture the effect of implemented measures directly both at the measure outlet and at the first downstream station. The station at the outlet of sub-catchment 57 is included in both datasets (*n* = 22). Some characteristics of these sub-catchments are presented in Table 1.

**Table 1:** ID code, area, land use distribution, number of properties, and number of people living in each sub-catchment included in the analysis. “Former arable” refers to percentage of arable land digitized from historical economic maps (1859–1934) for Björnöfjärden. The 2-digit ID: s are the outlet sub-catchments while the rest are located within sub-catchment 57

ID	Area ha	Wetland %	Arable %	Former arable %	Open land %	Forest %	Pasture %	Properties	People2022
27	75.4	4.6	0.0	4.2	10.3	76.9	1.6	13	29
31	68.7	0.7	0.0	4.4	25.3	66.5	0.0	85	133
32	163.5	4.8	1.1	17.5	26.6	68.2	0.0	287	350
48	12.2	0.1	11.9	14.2	10.1	74.1	3.8	0	0
49	4.1	0.0	0.0	23.4	24.3	75.7	0.0	0	0
50	57.6	7.4	0.0	6.0	13.9	78.7	0.0	45	81
58	118.8	3.1	0.0	12.5	24.6	72.4	0.0	118	288
59	39.7	4.7	0.0	6.0	11.5	83.8	0.0	20	23
60	32.8	0.0	11.9	20.7	21.9	66.1	0.0	17	15
57	378.3	2.9	10.5	17.2	12.9	73.7	0.0	28	45
5702	12.0	0.0	7.3	17.6	38.4	54.3	0.0	3	4
5712	358.0	3.1	10.6	17.3	11.7	74.6	0.0	31	42
5719	268.5	3.8	4.4	5.1	10.5	81.2	0.0	19	31
CW1in	31.4	0.0	33.1	34.2	9.1	57.7	0.0	1	3
LF1in	33.0	0.0	35.5	34.3	9.2	55.3	0.0	1	3
LF1out	33.0	0.0	35.5	34.3	9.2	55.3	0.0	1	3
CW2in	25.0	3.8	29.0	27.8	11.9	55.3	0.0	1	0
LF2in	26.8	4.2	28.3	30.0	13.2	54.3	0.0	1	0
LF2out	26.8	4.2	28.2	30.0	13.3	54.3	0.0	1	0
LF3in	35.2	0.0	2.5	10.9	38.5	59.0	0.0	11	13
LF3out	35.3	0.0	2.5	10.9	38.6	58.9	0.0	11	13
CW3out	384.1	3.0	10.3	17.1	13.0	73.7	0.0	29	45

Mitigation measures to reduce nutrient losses from diffuse sources included SL of all arable fields in the northwest part of the catchment, conducted in 2013 and 2014, as well as CWs, LFs, and LFDS, as indicated in Fig. 1. Two CWs (CW1 and CW2, Fig. 1) and two LFs (LF1 and LF2) had similar design. (Water first enters a CW and then flows into an LF.) These CWs and LFs were installed in 2013, with volume capacity of 20 m^3^ and 30 m^3^ of filter material (Hyttsand; granulated blast furnace slag) for LF1 and LF2, respectively. CW1 was constructed by broadening an existing ditch and is long (220 m) and narrow (12 m), whereas CW2 is 110 m long and 40 m wide at its widest part, and was created by damming marshland. The third LF (LF3, Fig. 1) receives water from horse paddocks, has volume capacity of 40 m^3^, and uses Filtralite P (manufactured from expanded clay material) as filter material. Water samples were taken before and after all CWs and LFs. The impact of any diffuse or point sources between the inlet and the outlet of the implemented measures was assumed to be minimal due to rather small spatial extent of the measures. CW3 (~ 1 ha) was installed in 2015 with the main purpose to function as a spawning area for pike migrating up from the bay. The tile drainage system in one field (LFDS) was repaired and new tile drains were installed in 2015, with 18 kg of quicklime per meter of ditch added when backfilling (Wesström, unpubl.).

Several measures were also introduced to reduce negative impacts from horse keeping, including frequent/regular picking up droppings in paddocks and pastures, repairing manure storage facilities, introducing buffer strips along ditches, and fencing off horses from ditches, mostly upstream in sub-catchment 57 upstream LF3 (Fig. 1).

Additionally, approximately 50% of 154 property owners, mostly in sub-catchments 32, 57, 59, and 60 (Fig. 1), improved their OWTs between 2013 and 2015 (Norström et al. 2016). A new OWT for wastewater from a conference facility with maximum accommodation capacity of 413 guests per week, and offering lunch for 80–160 guests per day, was built in summer 2014.

### Water discharge and water chemistry

Measurements of water discharge from the adjacent (12 km away) headwater catchment Stormyra (area 4 km^2^, (SMHI 2023)) covering the whole study period were used to describe different water flow regimes on the sampling occasions and to calculate loads. The representativeness of the flow data from Stormyra for the Björnöfjärden was examined by comparisons with the available daily flow measurements data from station 57 (Fig. 1) for period 2013–2019 and with monthly flow data from the LFDS field for period (2015–2019), using Kling–Gupta efficiency (Gupta et al. 2009) as a goodness-of-fit measure. The water discharge data were grouped into environmental flow components (EFC) using the Indicators of Hydrologic Alternation tool, version 7.1.0.10 (Richter et al. 1996), to investigate possible links between the effects of implemented mitigation measures and differences in flow regimes.

Water analyses were performed at the certified (since 1992) Erken laboratory at Uppsala University. The following parameters were analyzed and used in this study: pH, alkalinity, electrical conductivity (EC), suspended solids (SS, only samples from the northwestern sub-catchment), concentrations of phosphate-phosphorus (PO_4_-P), total phosphorus (TP), ammonium-nitrogen (NH_4_-N), nitrate/nitrite-nitrogen (NO_2_-N/NO_3_-N, hereafter called NO_3_-N), and total nitrogen (TN). More details about the analysis methods can be found in Rydin et al. (2017) and references within. In this paper, we focus on NO_3_-N, TN, PO_4_-P, and TP.

Daily loads were calculated by multiplying measured concentrations with recorded daily discharge at Stormyra station at the sampling date, with consideration taken to the upstream area of each sampling point. The daily streamflow at each sub-catchment was calculated using reference streamgage (Stormyra) with drainage area ratio method (Archfield and Vogel 2010), assuming that the streamflow per unit area at the ungaged catchment and reference catchment is equal.

### Trend analysis and statistics

Trend analyses were performed on monthly discharge and nutrient concentrations with the procedure described in von Brömssen et al. (2021), using general additive models (GAM) to detect increasing or decreasing trends at any time during the analyzed period. A regional Mann–Kendall (RMK) test (Helsel and Frans 2006) was used to check for cross-site trends, and a regular Mann–Kendall (MK) trend test was used for trend detection over the entire monitoring period for each station. The RMK and MK were performed separately for outlet sub-catchments (*n* = 10), for the stations within sub-catchment 57 (*n* = 13), and for the whole dataset (*n* = 22, sub-catchment 57 included in both datasets). Additionally, RMK and GAM were performed both on the monthly discharge from Stormyra station and daily discharges at the sampling occasions, and on the calculated daily loads. The R script from von Brömssen et al. (2021) was used for GAM trend analyses and production of trend plots, while RMK and MK were performed using the R package rkt (Marchetto 2021).

Analysis of variance (ANOVA) was performed to test for statistically significant differences in nutrient concentrations and loads between the inlet and the outlet of different implemented measures (CW1-3 and LF1-3), and Student’s *t*-Test was used for multiple comparisons of means for the same data. The same analysis was performed to test statistically significant differences in pH, EC, alkalinity and nutrient concentrations between different EFC. All variables were prior to further analyses tested for normality, and since non-normal distribution was shown, they were log10-transformed. Principal component analysis (PCA) (JMP 13.0.0, SAS Institute, Cary, NC) was performed to identify possible clustering, and Pearson correlation matrix, estimated by a pairwise method in PCA, was calculated.

## Results and discussion

### Water discharge to Björnöfjärden

Comparison of the available measured daily water flows from station 57 and monthly flow from LFDS field with the measured flow values from a nearby Stormyra station resulted in high goodness of fit (KGE = 0.91 and KGE = 0.80, respectively). The water discharge to Björnöfjärden followed a typical pattern for this part of Sweden; the two lowest flow components (extreme low flow and low flow; Fig. S1 in SM) comprised 81% of daily flow, but contributed only 28% of total flow volume. Both alkalinity and EC are used as alternative tracers in flow partitioning studies (Lazo et al. 2024), where higher EC values are coupled to old or pre-event water and low flows (Calvi et al. 2018). Indeed, in our case, data in Table S1 show that values of pH, EC and alkalinity are in majority of the outlet sub-catchment significantly higher during low flows (extremely low flow and low flow), when groundwater contribution is assumed to be proportionally higher, compared to higher flows (high-flow pulse and small flood), indicating consequent patterns between runoff and water chemical composition. The two highest flow components (small and large floods; Fig. S1 in SM) comprised only 3% of daily flows, but contributed 19% of total flow volume. High-flow pulses were responsible for the largest proportion of flow volume (53%), during only 16% of the time.

### Nutrient concentrations at sub-catchment outlets to Björnöfjärden

**Fig. 2:**
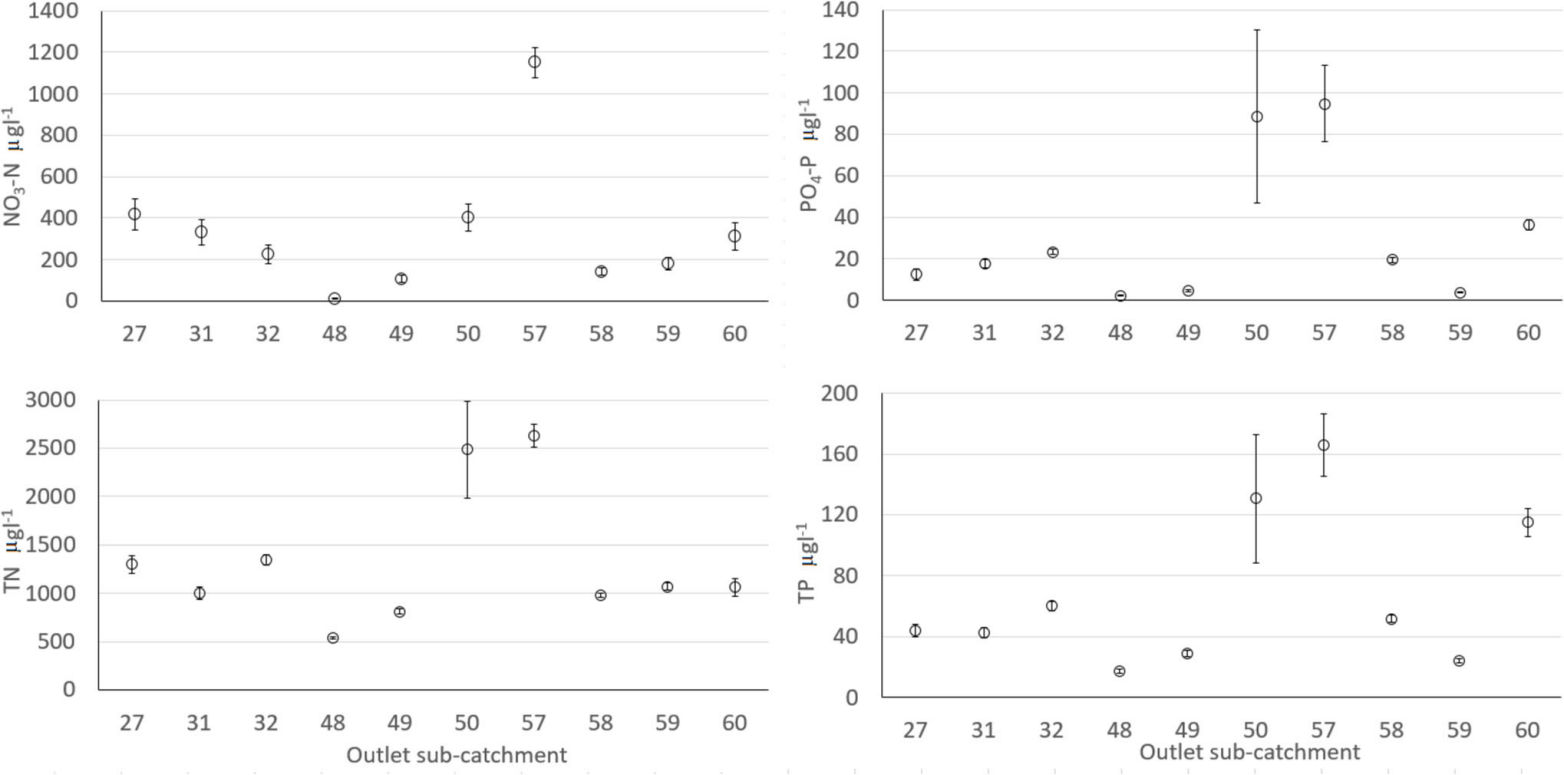
Mean value and standard error of concentrations of nitrate-N (NO_3_-N), total nitrogen (TN), phosphate-phosphorus (PO_4_-P), and total phosphorus (TP) at outlet stations in the 10 main sub-catchments for period 2012–2022

Nutrient concentrations at the outlet of the 10 main sub-catchments showed large variations. Sub-catchment 57 had the highest mean values for all four nutrient compounds studied (NO_3_-N, TN, PO_4_-P, and TP), while sub-catchment 48 had the lowest mean values (Fig. 2).

**Fig. 3:**
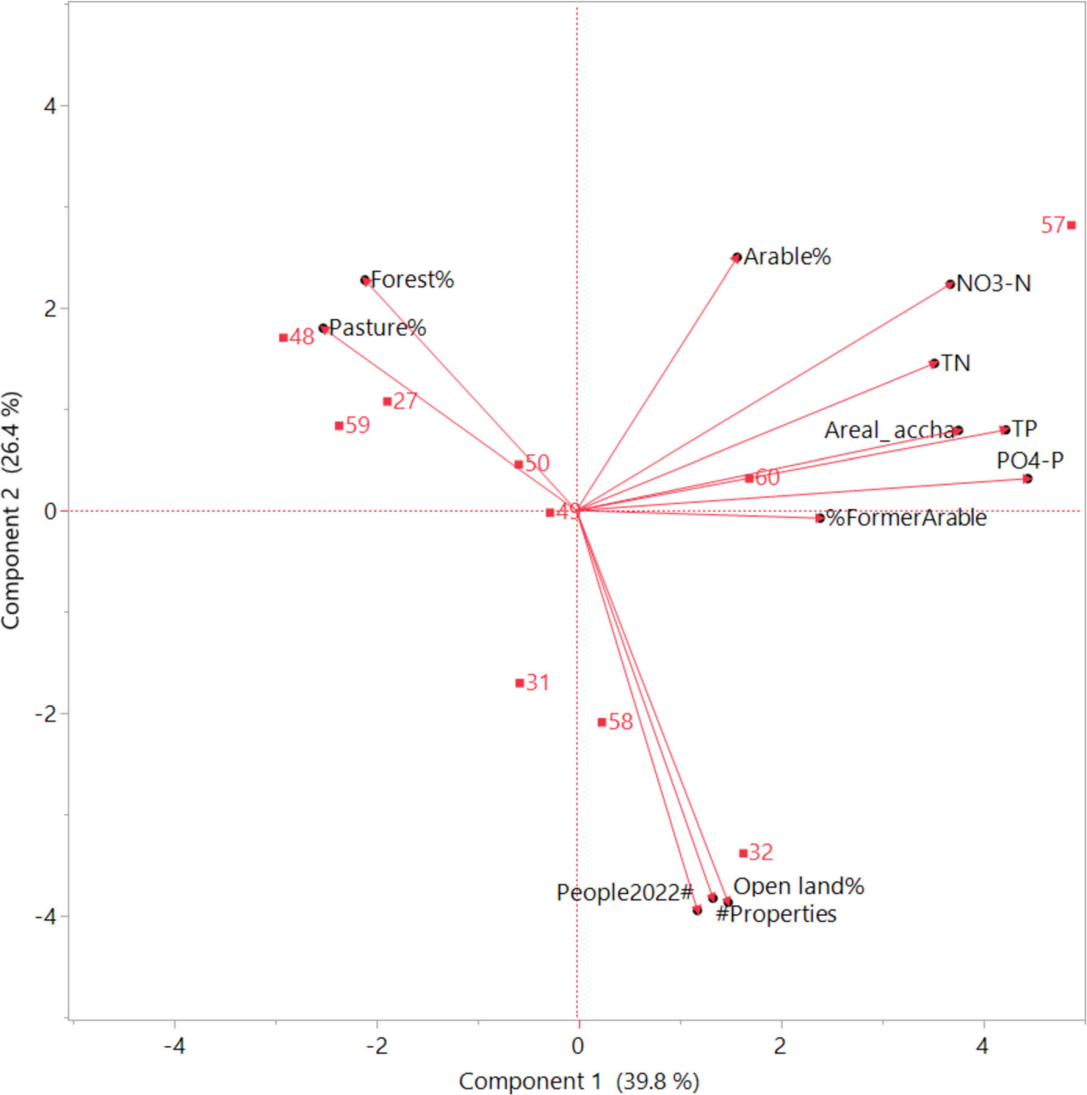
Principal component analysis plot of correlations between concentrations of different nutrient constituents (NO_3_-N, TN, PO_4_-P, and TP) and a set of sub-catchment characteristics, including catchment area (Areal_accha), population (People2022#), number of properties (#Properties), land use distribution (Pasture%, Forest%, Arable%, Open land%), and proportion of former arable land (%FormerArable) digitized from historical maps (1859–1934)

PCA on median values of water constituents and including important sub-catchment characteristics such as area, land use distribution, and population showed that the two first principal components explained 66.2% of the total variance (Fig. 3). However, the correlation matrix (data not shown) indicated generally weak correlations. There was a strong correlation between the nutrient fractions PO_4_-P and TP (*r* = 0.95), and NO_3_-N and TN (*r* = 0.86), respectively. There was also a strong correlation between sub-catchment area and concentrations of all nutrient constituents (*r* = 0.59, 0.73, 0.84, and 0.87 for TP, PO_4_-P, TN, and NO_3_-N, respectively), which could be explained by the overwhelming influence of the largest sub-catchment 57. Therefore, this correlation does not imply causation. Weak (*r* = 0.36 for NO_3_-N) or moderate (*r* = 0.46 and 0.48 for TP and PO_4_-P, respectively) positive correlations were found between these constituents and proportion of arable land. Interestingly, there was a strong correlation (*r* = 0.60) between TP and proportion of former arable land (Table 1, Fig. 3). There was a strong influence of pasture and forest, which are associated with low nutrient concentrations, in sub-catchments 59, 27, and especially 48 (Fig. 3). Reported correlations between nutrient concentrations and land use categories, primarily arable land, are usually much stronger (Wilson 2015; Djodjic et al. 2021). However, in the study area the proportion of arable land in different sub-catchments was at most 10–12%. Additionally, the nutrient and primarily P legacy (Sharpley et al. 2013) of the considerable area of former arable land converted into forest, or more often into peri-urban areas, may blur the connections between current land use and nutrient concentrations.

### Trend analyses of nutrient concentration and loads

**Fig. 4:**
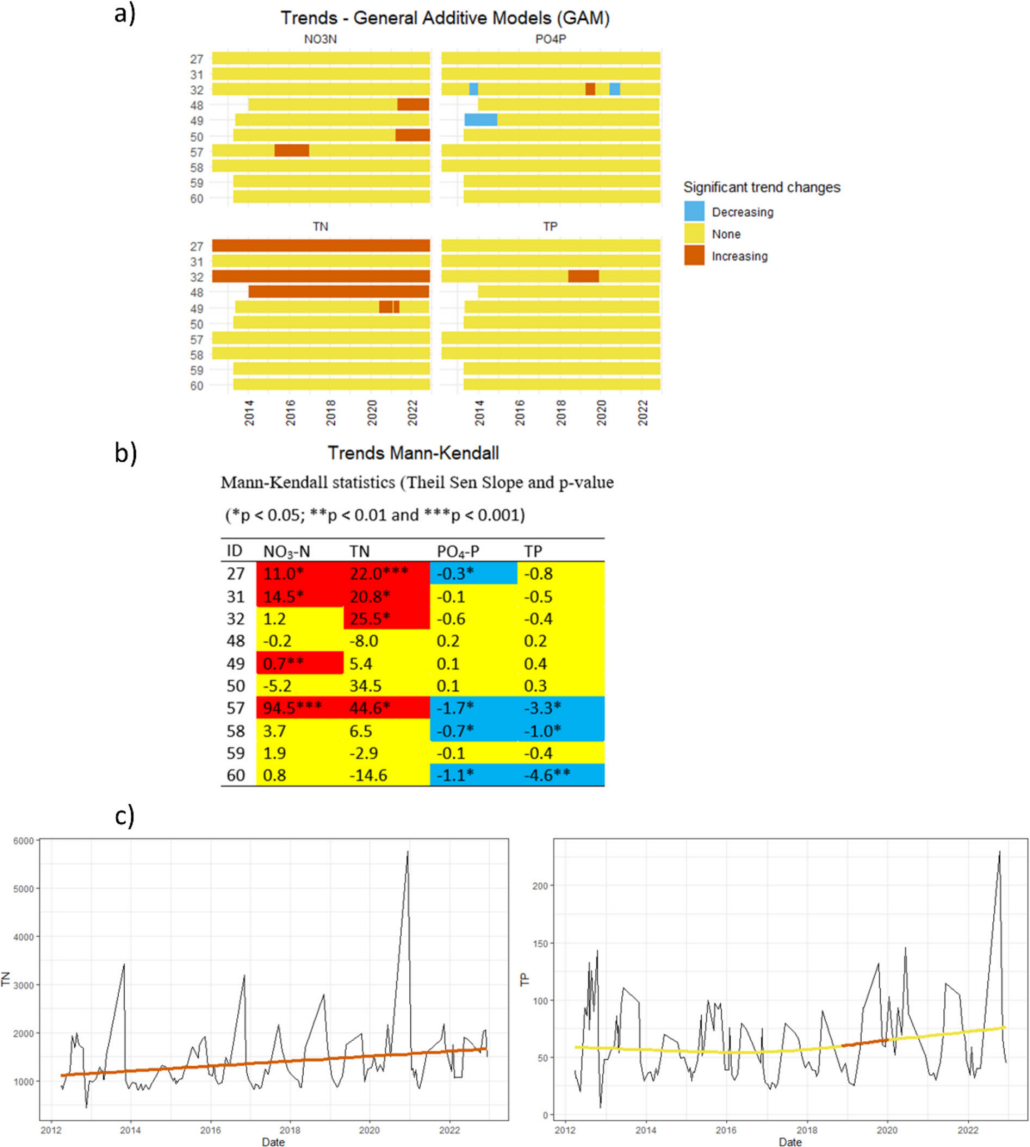
Trends in concentrations of total phosphorus (TP), phosphate-phosphorus (PO_4_-P), total nitrogen (TN), and nitrate-N (NO_3_-N), as calculated with **a** general additive models (GAM) and **b** Mann–Kendall test, and **c** examples of (left) an individual continuous increasing trend in TN and (right) a temporary increasing trend in TP calculated with GAM for sub-catchment 32. Blue color indicates a period with a significant decreasing trend, yellow no significant trend, and red a significant increasing trend

RMK tests including the concentrations at 10 outlet monitoring stations identified increasing trends for both NO_3_-N and TN, but no significant trends for PO_4_-P and TP. Neither GAM nor RMK (*p* = 0.095) showed any significant trend in monthly water discharge. Similarly, there was no significant trends in daily water discharges at the sampling occasions (Fig. S2 in SM). Additionally, there were no significant correlations between water discharge and any of the studied nutrients for the outlet station 57 (*p* > 0.05). Few significant individual trends in concentrations over time at the outlet stations were detected using GAM (Fig. 4). Out of 40 time series (10 sites × 4 compounds), there were nine increasing trends (of which most for TN (4) and NO_3_-N (3)) and only two decreasing trends (both for PO_4_-P). The MK tests showed a higher number of significant individual trends, but the magnitude of these trends (Theil Sen slope) was rather low (Fig. 4). However, none of these concentration trends was confirmed for loads (Fig. S3). Water discharge, although there were no significant trends, had a smoothing effect on already low-magnitude concentration trends. Some concentration trends, particularly those with highest significance level (*p* < 0.001), were identified by both methods.

There were no significant decreasing GAM trends in PO_4_-P or TP concentrations (Fig. 4a) at the outlet of sub-catchment 57, where most of the mitigation measures were implemented (Fig. 1). On the other hand, MK tests identified decreasing trends at this station for both PO_4_-P and TP, but with low magnitude and the lowest significance level (Fig. 4b). Both trend analysis methods showed a significant increasing trend in NO_3_-N in this sub-catchment (Fig. 4). In sub-catchments 27 and 32, increasing trends were recorded for TN with both GAM and MK (Fig. 4). According to Statistics Sweden (Statistics Sweden 2024b), the population in the whole Ingarö district (120 km^2^ including Björnöfjärden bay catchment) increased by 7% between 2015 and 2022. Closer scrutiny revealed that the population increases were even larger in sub-catchments 27 and 32 (26 and 9%, respectively). Although there were some differences between GAM and MK regarding detected trends, the general conclusion was that changes over time are slow and of low magnitude. Phosphorus, the target nutrient, showed either no significant trends or trends with low magnitude, with the highest estimated decreasing trend of less than 5 μg L^−1^ yr^−1^ TP in sub-catchment 60. None of the countermeasures within the remediation project was placed in sub-catchment 60, but possible undocumented improvement of OWTs may be the reason for the decreasing trend. The number of identified significant GAM trends was lower for daily loads, with only five significant temporarily trends (Fig. S3). There were no significant trends in loads according to RMK. The GAMs are usually robust against outliers, unless they are in the beginning or in the end of the time series. Therefore, the identified decreasing trends in TP and PO_4_-P loads in sub-catchment 59 (Fig. S3) should be interpreted carefully, as the time series start with a very high daily load. The same applies for the NO_3_-N load time series for sub-catchments 49 and 59, where both end with a high load value resulting in an increasing trend.

According to Betanzo et al. (2015), the minimum duration of monitoring at monthly time step required in small watersheds to detect water quality change is 8 years, assuming 40% reductions in PO_4_-P and TP over a period of 20 years. Likewise, Wellen et al. (2020) estimated that detection of 40% reductions in flow-weighted mean concentrations would require 3–10 years of TP data, 5–25 years of PO_4_-P data, and 2–6 years of NO_3_-N data. In our case, the possible reductions were of much lower magnitude (Table 2). Therefore, the detected trends should be viewed with caution, especially those involving low magnitude of change at the lowest significance level. A combination of GAM and MK tests, for both concentrations and loads, as used here, may be helpful when interpreting the results, to avoid reliance on the results of one single method, given the high uncertainty.

**Table 2:**
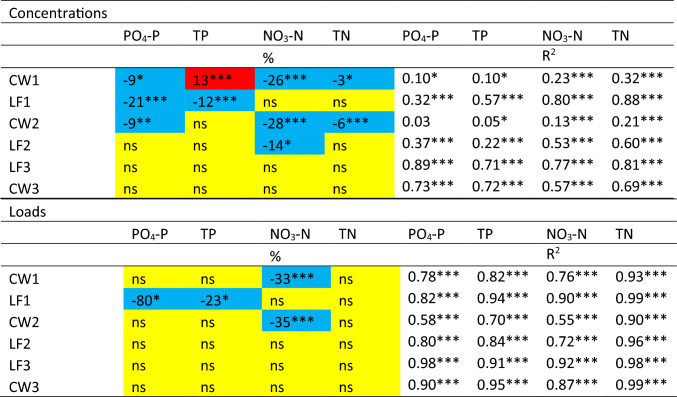
Effect (%) of implemented mitigation measures in reducing nutrient concentrations and loads, based on ANOVA comparison of incoming and outgoing nutrient concentrations, and coefficient of determination (R_2_) between incoming and outgoing nutrient concentrations. Blue indicates significant reductions, yellow no significant differences, and red a significant increase. Level of significance: *p  < 0.05; **p  < 0.01, ***p < 0.001

### Effects of implemented mitigation measures

**Fig. 5:**
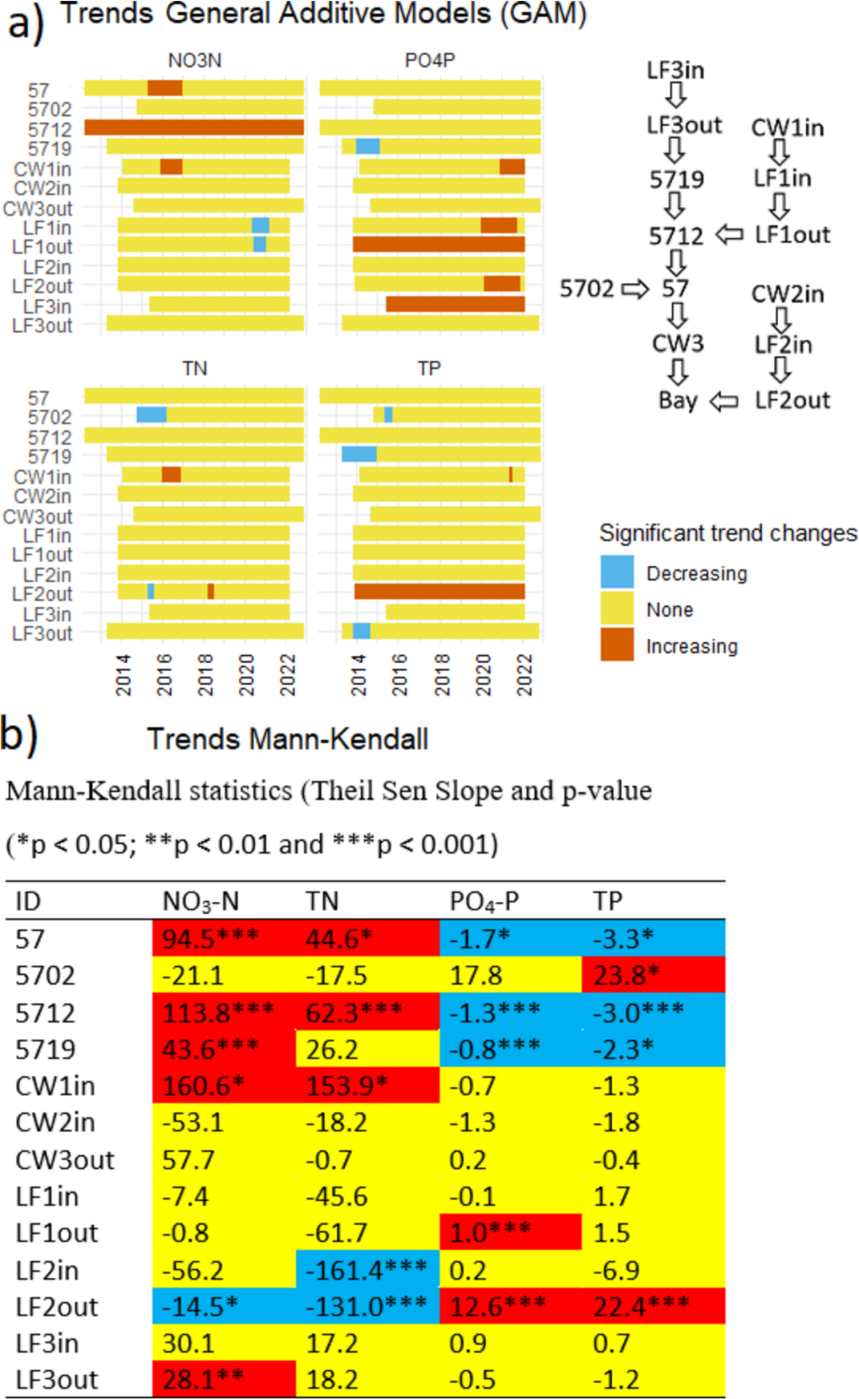
Trends in concentrations of total phosphorus (TP), phosphate-phosphorus (PO_4_-P), total nitrogen (TN), and nitrate-nitrogen (NO_3_-N) in sub-catchment 57, calculated with **a** general additive models (GAM) and **b** Mann–Kendall test, with a flow scheme (to the right) showing how stations connect to each other. Blue indicates a period with a significant decreasing trend, yellow no significant trend, and red a significant increasing trend

Potential effects of implemented mitigation measures can be evaluated by studying temporal trends and, if possible, by comparing nutrient concentrations at the inlet and outlet of each individual measure. Here, we start with the evaluation of the effects of groups of implemented measures (SL and LFDS; measures at horse farms; CWs and finally LFs) and thereafter continue to follow the effects at the downstream monitoring stations (5719, 5712, 5702, and 57, Figs. 1 and 5) in the small stream.

Structure Liming (SL) and Lime Filter Drainage Systems (LFDS).

Structure liming was applied on arable fields upstream of 5712, CW1in and CW2in (Fig. 1). There were no significant decreasing trends in PO_4_-P or TP at neither of these CW inlet stations (CW1 and CW2), whereas small but significant downwards MK trends in concentrations of PO_4_-P and TP (1.3 and 3.0 μg L^−1^ yr^−1^, respectively) at station 5712 (Fig. 5b). This downward trend was confirmed with GAM for PO_4_-P loads, but not for TP (Fig. S4). However, the measurement period before SL was very short (5712) or nonexistent (CW1in and CW2in), and therefore, it is difficult to say whether SL had an effect on nutrient concentrations compared with the previous situation. Additionally, there were several other countermeasures conducted upstream station 5712 (CW1, LF1, LF3 and improvements in horse keeping, Fig. 1), and therefore, the mentioned decreasing trends cannot be attributed solely to SL. Bieroza et al. (2019) found a significant effect on both turbidity and TP concentrations. In our study, P concentrations were very low in both control and lime filter drain pipes (usually < 20 μg L^−1^), and actually lower than the P concentration in the downstream main ditch draining the field. The presence of acid *gyttja* in subsoil may have counteracted any effects of the limited amount lime material (18 kg of quicklime per meter of ditch). *Gyttja* is a Swedish term referring to quaternary deposits with low pH, high clay and organic carbon content (Klöffel et al. 2024), stable structure, and well-developed cracks in deeper subsoil (Svanbäck et al. 2014). This field was also structure limed in 2013, prior to installation of LFDS, so the very low TP and PO_4_-P concentrations may be results of liming (Ulén and Etana 2014; Blomquist 2021). However, nutrient concentrations from this field were not measured prior to SL. Additionally, N losses from this field were rather high compared to similar clay soils, reaching concentrations between 15 and 20 mg L^−1^ (Wesström, unpubl.). Bieroza et al. (2019) showed also a significant (45%) increase in NO_3_-N concentrations in lime filter drains. A rapid increase in NO_3_-N concentrations from ~ 5 mg L^−1^ in 2016 to > 15 mg L^−1^ (data not shown) observed in all replaced tile drains (both with and without lime filter) might have contributed to the increasing trend in NO_3_-N concentrations at downstream station 5712 (Fig. 5). Wesström et al. (2015) reported similar increases in NO_3_-N concentrations in both shallow groundwater and drain water after drainage system repair in a clay soil in western Sweden. Improved subsurface drainage generally reduces losses of P and organic N, whereas it increases losses of NO_3_-N and soluble salts (Skaggs et al. 1994).

#### Measures at horse farms

At the sampling station downstream the main horse farm (5719, Fig. 1), there were significant temporarily downward trends in the concentrations (GAM and MK analyses, Fig. 5) and loads (GAM, Fig. S4) of PO_4_-P and TP. This decrease coincided with implemented measures such as picking up droppings, repairing manure storage facilities, introducing buffer strips along ditches, and fencing off horses from ditches, which was introduced in 2014 and 2015 (Owenius 2015). Such measures can be expected to reduce P concentrations, which often are higher in the vicinity of horse facilities (Kumblad et al. 2024). However, the lime filter (LF3) did not contribute to this decrease (Table 2; section Lime Filters).

#### Constructed wetlands (CW)

Pairwise comparison of concentrations and loads at the inlet and outlet of CWs gave contrasting results (Table 2). For concentrations, TP increased by 13% at the outlet of CW1, whereas PO_4_-P, NO_3_-N, and TN decreased, by 9, 26, and 3%, respectively. For loads, there was only one significant trend for NO_3_-N (decreasing 33%) for this station. The decrease in PO_4_-P concentrations and increase in TP concentrations in CW1 (Table 2) indicate mobilization of soil particles and associated bound P within this wetland. In fact, there was a 29% increase in mean SS concentration at the outlet of CW1 compared with the inlet, mostly due to high SS concentrations immediately after CW construction in 2014–2015 (data not shown). In a systematic review of published studies on constructed and restored wetlands, Land et al. (2016) reported variation in median retention between 6.3 and 29 kg P ha^−1^ and 14 and 140 kg N ha^−1^. High area-specific load (Weisner et al. 2016; Djodjic et al. 2020, 2022) is key for achieving high retention potential. However, CW1 received low TP (16.6 kg P per ha CW and year) and moderate TN (1021 kg N ha^−1^ yr^−1^) loads. Together with the mean TN reduction of 3% (Table 2), this resulted in mean annual retention of approximately 30 kg TN per ha and year, which is in the lower part of the range reported by Land et al. (2016).

CW2 had somewhat higher incoming loads (103.6 kg P ha^−1^ and 1470 kg N ha^−1^) and showed significantly decreasing concentrations for all constituents except TP (Table 2). The 6% reduction in TN resulted in a reduction of 88 kg TN ha^−1^ yr^−1^. Once again, only one significant trend was identified for loads (decreasing NO_3_-N trend, 35%, Table 2). Although the inlet/outlet differences were not significant, TP concentrations at the outlet of CW2 were generally lower than at the inlet, with a tendency for greater differences in later years. As the assumed life span of a CW is 20 years, the 8 years of data presented here needs to be prolonged to capture the whole active period.

CW3 showed no significant trends (Table 2), as it was not designed to retain nutrients but to provide a spawning area for pike migrating up from Björnöfjärden bay. Kynkäänniemi (2014) found a strong positive linear correlation (*R*^2^ = 0.78) between hydraulic load (HL) and annual TP accumulation up to a HL threshold of approximately 120 m yr^−1^, while Djodjic et al. (2020) proposed HL = 100 as an optimal value for the determination of the water area of CWs, with some safety margins to the above-mentioned 120 m yr^−1^. CW1 and CW2 were well below this value, with HL values of 29 and 25, respectively, whereas CW3 was rather close, with HL of 95. This means that both CW1 and CW2 are too large in relation to their catchment, which lowers their nutrient load per wetland area, and consequently their nutrient retention and cost efficiency (Djodjic et al. 2022).

#### Lime filters (LF)

**Fig. 6:**
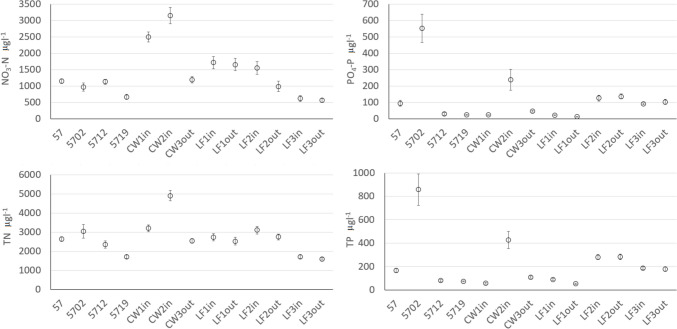
Mean value and standard error of the concentrations of nitrate-nitrogen (NO_3_-N), total nitrogen (TN), phosphate-phosphorus (PO_4_-P), and total phosphorus (TP) at the 13 stations in sub-catchment 57 for period 2012–2022

Concentrations and loads of PO_4_-P and TP were significantly lower at the outlet of LF1 compared with the inlet (Table 2, Fig. S6). Incoming concentrations of both PO_4_-P and TP were considerably lower in LF1in (mean value 21 and 90 μg L^−1^, respectively) compared with LF2in (mean values of 126 and 280 μg L^−1^, respectively) (Fig. 6). The concentration reductions seemed more pronounced during lower flows in LF1 (Fig. S6 in SM). In contrast to LF1, the mean values over the entire period indicated no significant reductions in PO_4_-P or TP in LF2 (Table 2). However, further analysis revealed that the concentrations of TP were generally lower at the outlet than at the inlet of LF2 in the first half of the study period (2014–2017) (Fig. S7 in SM). In contrast, in the second half of the study period (2018–2022), TP concentrations were higher at the outlet than at the inlet of LF2. Thus, LF2 seemed to be efficient in TP reduction during the first 2–3 years. As the high TP concentrations reaching LF2 continued, the active material in LF2 may have been saturated with P and started to act as a PO_4_-P source (Fig. S4 in SM). Ekstrand et al. (2011) recorded average retention over 9–18 months of 38 and 36% for PO_4_-P and TP, respectively, in three comparable LF facilities in central Sweden using the same filter material (Hyttsand). This is in line with results presented here for LF2 during the initial operating period of 2–3 years (Fig. S7), but our results clearly show that longer evaluation periods are needed as the filter function is not constant. There were no significant effects of LF3 on any parameter (Table 2). The incoming and outgoing PO_4_-P and TP concentrations in LF3 were highly correlated (*R*^2^ = 0.89, *p* < 0.0001, Table 2), indicating no significant effect of the LF. A possible explanation might be that preferential flow may have occurred within LF3, with the bulk of incoming water flowing through the filter without interaction with the filter material. Coefficient of determination between incoming and outgoing concentrations in the LFs was generally higher in cases where no significant changes occurred (Table 2). Kirkkala et al. (2012) studied nutrient removal in three on-site lime–sand filters and concluded that filters are especially suitable for treatment of P-rich waters, but the dimensioning criteria is not fully understood (as in L2) and that it might be difficult to ensure sufficient contact between incoming water and filter material under varying field conditions (as in L3).

Trend analysis with GAM also revealed increasing trends in PO_4_-P concentrations (Fig. 5a), comprising either constantly increasing trends (LF1out, LF3in) or temporarily increasing trends in the later part of the study period (CW1in, LF1in, and LF2out). In the case of LF1out and LF2out, the increasing trends suggest a successive increase in filter P saturation and possible conversion of LF from sinks into sources. These trends were confirmed by MK tests. Increasing GAM trend for PO_4_-P load was confirmed for LF2out, but not for other stations (Fig. S4).

The pattern of lower outgoing concentrations during low flows, and either similar or higher outgoing concentrations during higher flows, was observed for several of the implemented measures and nutrient parameters, even in cases without significant reductions, for instance in CW3 (Fig. S6). This indicates a need to slow incoming flows and increase water residence time in order to increase nutrient retention in the LFs. For CWs, high flows in this case mean also dilution and lower concentrations (Fig. S6), which emphasize need for proper targeting and placement of CWs in areas with high nutrient concentrations/loads per unit of CW area.

#### The effects at downstream stations

In the study area, regional Mann–Kendall (RMK) tests including 13 monitoring stations before and after implemented measures identified no significant trends in concentrations or in loads for any parameter (water discharge, NO_3_-N, TN, PO_4_-P, and TP). The same applied for the dataset including all 22 stations (10 outlet sub-catchments plus 13 before/after stations, where station 57 is common to both subsets). Closer inspection of individual concentration trends in sub-catchment 57, where most mitigation measures were implemented, revealed contrasting trends for different water constituents and measuring stations, and the two test methods used (GAM and MK, Fig. 5).

Upstream station 5719 (Fig. 1) was discussed above in section dealing with implemented measures at horse farms.

Station 5712 receives water from upstream arable land treated with mitigation measures SL, LFDS, CW1, LF1, and LF3 and possible improvements in horse keeping (5719). The concentration trends at this station follow the increasing MK trends in NO_3_-N recorded for upstream stations 5719 and CW1in, and the decreasing MK trends in PO_4_-P and TP from upstream station 5719 (Fig. 5b). However, only the increasing NO_3_-N trend was confirmed with GAM (Fig. 5a). The time lags and legacy sources with long groundwater transit times, especially for nitrogen, could blur and delay the positive effects of the mitigation measures (Meals et al. 2010; Bieroza et al. 2019; Basu et al. 2022; Sandström et al. 2024). Similarly, terrestrial P legacies including prior nutrient and land management activities with buildup of soil P content represent a slow and tortuous pathway which may mask or buffer the impact of implemented countermeasures (Sharpley et al. 2013). The above-mentioned strong correlation between the area of former arable land and TP concentrations (Fig. 3) represents potentially an example of the legacy effects. As mentioned earlier, there were distinct differences between low and high flows in alkalinity, EC, and pH (Table S1). On the other hand, nutrient responses to different flow regimes varied among different groups of sub-catchments. Sub-catchments 50 and 57 had significantly higher TN, PO_4_-P, and TP during low flows, indicating large influences of point sources (Helsel and Hirsch 2002). In contrast, sub-catchments 27, 31, 48, 49, and 59 showed low nutrient concentrations regardless flow regime, and there were few significant differences in nutrient concentrations between EFCs (Table S1), indicating low negative impacts on water quality. Finally, sub-catchments 32, 58, and 60 behaved in the same way, with few significant differences between flow components (Table S1), but at considerably higher levels of nutrient concentrations. Sub-catchments 32 and 58 had low share of arable land today (1.1 and 0%, respectively, Table 1) but considerably higher in the past (17.5 and 12.5, respectively, Table 1). High nutrient levels in these sub-catchments even at low flows dominated by groundwater with related time lags may indicate legacy sources and thereby hide eventual positive effects of implemented conservation measures (Basu et al. 2022).

GAM tests indicated significant decreasing trends in TN and TP concentrations (Fig. 5a) and loads (Fig. S4) at station 5702, which encompasses a conference facility, brewery, and cider-making plant, and showed very high mean concentrations of all constituents and the highest concentrations of PO_4_-P and TP (Fig. 6). Construction of a new OWT system in 2014 to treat sewage from the conference facility coincided with the above-mentioned decreasing trends. However, this trend was not confirmed by the MK test. The MK test for TP concentrations at this station in fact showed an increasing trend (Fig. 5b). This discrepancy is explained on examining the individual plot for this station (Fig. S5). GAM recognized the initial temporary decreasing trend, whereas MK identified the small increasing linear trend for the whole period, emphasizing the value of using both methods to get a more balanced picture.

Finally, the increasing MK trends of NO_3_-N and TN concentrations as well as the decreasing trends in PO_4_-P and TP concentrations from upstream stations 5712 and 5719 were confirmed at the outlet station 57 (Fig. 5b). However, only the increasing trend in NO_3_-N concentration was confirmed by GAM analyses (Fig. 5a) and no significant trends in loads were detected at station 57 (Fig. S4).

### Lessons learned

The ambition level and implementation rate in important efforts to reduce eutrophication in Sweden (Swedish Agency for Marine and Water Management 2022) and elsewhere in the world need to be intensified. However, systematic monitoring and reporting on existing mitigation measures before, during, and after implementation are rare, resulting in lack of data on mitigation efficiency and possible effects on water recipients downstream. Recent findings showing a lack of improvement in water quality (Tomczyk et al. 2023; Sandström et al. 2024) and in biodiversity connected to water quality (Haase et al. 2023) in response to implemented mitigation measures highlight the need to refine and improve mitigation strategies, especially regarding diffuse nutrient losses. This study demonstrated the importance of several aspects before, during, and after implementation of mitigation measures:

1. *Doing the right thing* proper targeting of nutrient sources (agriculture, OWTs, horse keeping) and selection of suitable mitigation measures is essential. In this regard, the decreasing trends in PO_4_-P and TP indicate correct targeting and mitigation of pollution sources within horse keeping operations as promising (Kumblad et al. 2024). Implemented measures (picking up droppings, repairing manure storage facilities, introducing buffer strips along ditches, and fencing off horses from ditches) targeting direct P sources at horse farms were more successful in decreasing P concentrations than the measures targeting to reduce P concentrations in the ditch (LF). SL has been earlier used as an efficient measure to P losses (Ulén and Etana 2014; Norberg and Aronsson 2022), and the measured P concentrations in both control and lime filter drain pipes in LFDS field may be a result of SL.
2. *Right measure in the right place* the efficiency of mitigation measures is highly site-specific. In the case of CWs, the nutrient retention efficiency (Land et al. 2016) and cost efficiency (Djodjic et al. 2022) are highly dependent on the nutrient loading rate. Despite somewhat lower nutrient concentrations at the outlets of CW1 and CW2, the total effect was small due to low hydraulic and nutrient loading rates, indicating that the location of these CWs is not most favorable. In contrast, CW3 was placed optimally in regard to the hydraulic load (right place), but not designed to reduce nutrients (not right measure). Usually, CWs designed to reduce P losses consist of a deeper sedimentation basin at the inlet allowing particle sedimentation followed by a shallow area planted with macrophytes where chemical sorption and biological P uptake can take place (Kynkäänniemi 2014). LFDS were shown as an efficient measure to reduce P losses (Bieroza et al. 2019) but at much higher incoming P concentrations compared to this study, where LFDS was not able to lower already low P concentrations (not right place). Addressing the sources of the pollution directly, as done with horse keeping operations, is strongly recommended. In spite of the long history of using the concept of critical source areas (CSAs) to reduce nonpoint source pollutants (McDowell et al. 2024), their identification and targeting are still recognized (Liu et al. 2024) as one of important research and management needs, especially as in some cases agri-environmental policy aims at extensive coverage, not at spatial precision, which contradicts the principles of CSA.
3. *Doing things right* the implementation phase of mitigation measures regarding their design and size is crucial. Securing proper size and design of CWs in relation to incoming water and nutrient load and proper balancing of the filter size and sorption capacity of the materials used in LFs in relation to incoming P concentrations and predicted longevity of the measures are preconditions for their proper functioning in the long term.
4. *Consider the time aspect and maintenance needs*: the increasing concentrations of PO_4_-P and TP in the outlet of LF and distinct change from sink to source emphasize the importance of including the time aspect and maintenance needs after implementation of mitigation measures. The life span of LFs was assumed to be 20 years, but, based on the results presented here, it seems to be much shorter and dependent on incoming concentrations and loads. The same life span (20 years) was assumed for CWs, highlighting the limitations of shorter monitoring programs attempting to evaluate the effects of CWs on nutrient retention. Further, continuous and consistently maintenance of recommended daily routines at horse farms and securing long-term functioning of OWTs are needed to prevent increasing nutrient trends.

## Conclusions and future implications

Various and temporally variable nutrient sources, current and past management, changing land use patterns, and spatial heterogeneity in highly dynamic systems hamper reliable estimation of the effects of mitigation measures and detection of change in water quality, especially at catchment scale at some distance downstream from implemented measures. Low measurement frequency, short monitoring periods, legacy sources, low (here maximum 28%) and flow-dependent nutrient reductions may further limit the ability of statistical methods to reliably detect trends. However, using several methods for trend detection (GAM, MK), together with inlet/outlet comparisons as well as detailed studies looking into different phases (initial vs later stages) and hydrological regimes (low vs high flows) during the countermeasures’ lifetime is recommended to further reveal processes in complex natural systems. The results presented here show that the implementation phase, with proper selection, placement, design, and dimensioning of mitigation measures and clear consideration of local preconditions (incoming hydraulic and nutrient load), is important. Some measures did not have any detectable effect because of inappropriate design (L3) or placement (LFDS). Others had short-lived effects (L1, L2), probably because of limited longevity of the filter material, turning them into P sources in the second half of the study period. The long life span of wetlands emphasizes the importance of suitable sampling frequency and a long follow-up period. Constructed wetlands had some positive effects on nutrient retention, but hydraulic load, and consequently nutrient load, was too low to achieve high retention efficiency. Additionally, nutrient retention seemed to be higher during low flow periods compared with peak flows. As low flows prevail during most of the year, reductions in nutrient concentrations might be more ecologically important for the local stream than for nutrient loads to downstream water recipients. This raises the question of whether measures to reduce nutrient loads should target reductions in water volume, rather than in nutrient concentrations, by attenuating and slowing water flows in the landscape.

Despite the ambitious monitoring program reported here, it was not always possible to obtain measurements before and after implementation of all individual measures (e.g., SL). Considering the large investments in mitigation programs to reduce nutrient losses and the rather modest results achieved so far, together with the increasing need for future mitigation actions, investing in comparable follow-up studies might help improve both the nutrient and cost efficiency of implemented measures.

## Supplementary Information

Below is the link to the electronic supplementary material.

Supplementary file1 (PDF 1472 KB)

## Data Availability

The authors declare that the data supporting the findings of this study are available within the paper and its Supplementary Material files. Should any raw data files be needed in another format, they are available from the corresponding author upon reasonable request.
