# Supplementary material for: Usual suspects meet mission impossible: Nutrient losses and effects of mitigation measures on a coastal catchment in the Baltic Sea region
Source: Ambio. 2025 Feb 3;54(6):1026–42. doi: 10.1007/s13280-025-02132-w (PMC12055691; doi:10.1007/s13280-025-02132-w)
Supplement: Supplementary file 1 — Supplementary file1 (PDF 1472 KB) [file 13280_2025_2132_MOESM1_ESM.pdf]

Usual suspects meet mission impossible: Nutrient losses and effects of mitigation measures on a coastal catchment in the Baltic Sea region

Faruk Djodjic[1*], Oksana Golovko[1], Linda Kumblad[2], Emil Rydin[2], Sara Sandström[3], Elin Widén-Nilsson[1]

[1]Department of Aquatic Sciences and Assessment, Swedish University of Agricultural Sciences, P.O. Box 7050, 750 07 Uppsala, Sweden.

[2]Baltic Sea Center, Stockholm University, SE-106 91 Stockholm, Sweden.

[3]Department of Soil and Environment, Swedish University of Agricultural Sciences, Box 7014, 750 07 Uppsala, Sweden.

Ambio

Supplementary Information

This supplementary information has not been peer reviewed.

Title: **Usual suspects meet mission impossible: Nutrient losses and effects of mitigation measures on a coastal catchment in the Baltic Sea region**

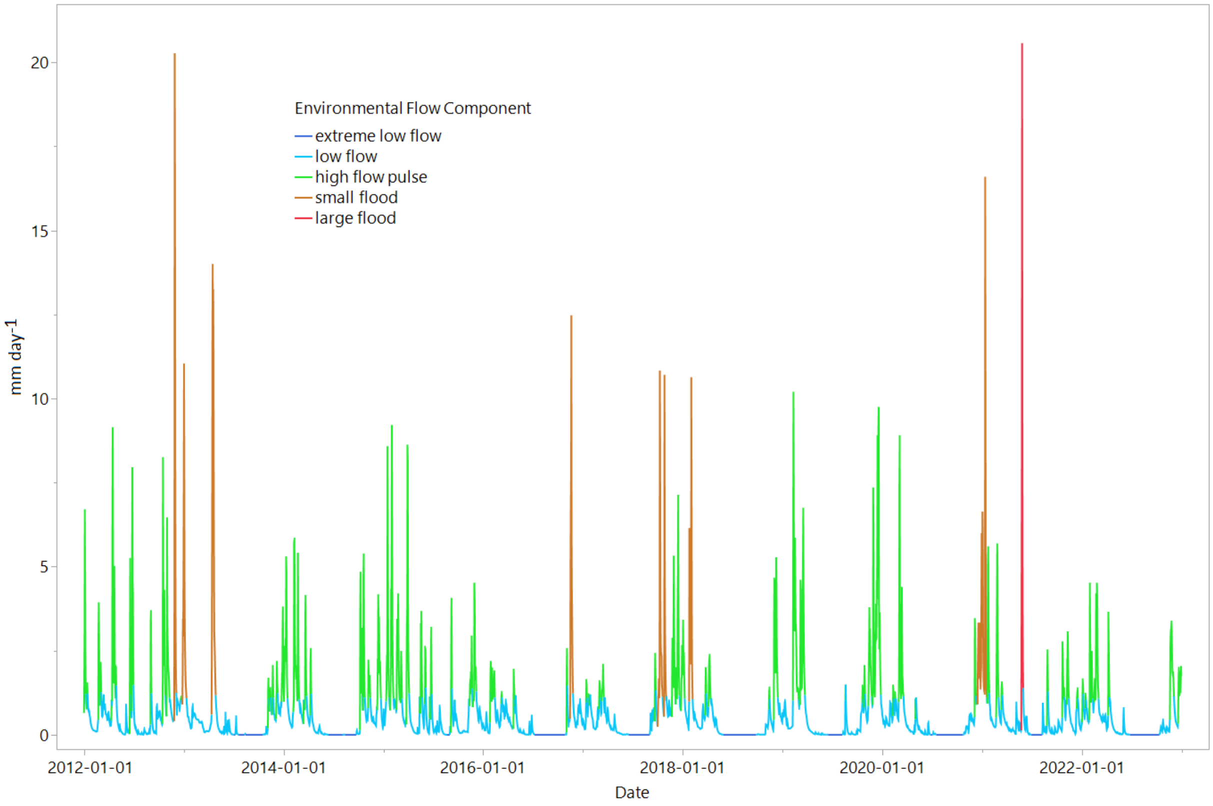

Figure S1. Environmental flow components of daily runoff measurements at Stormyra station.

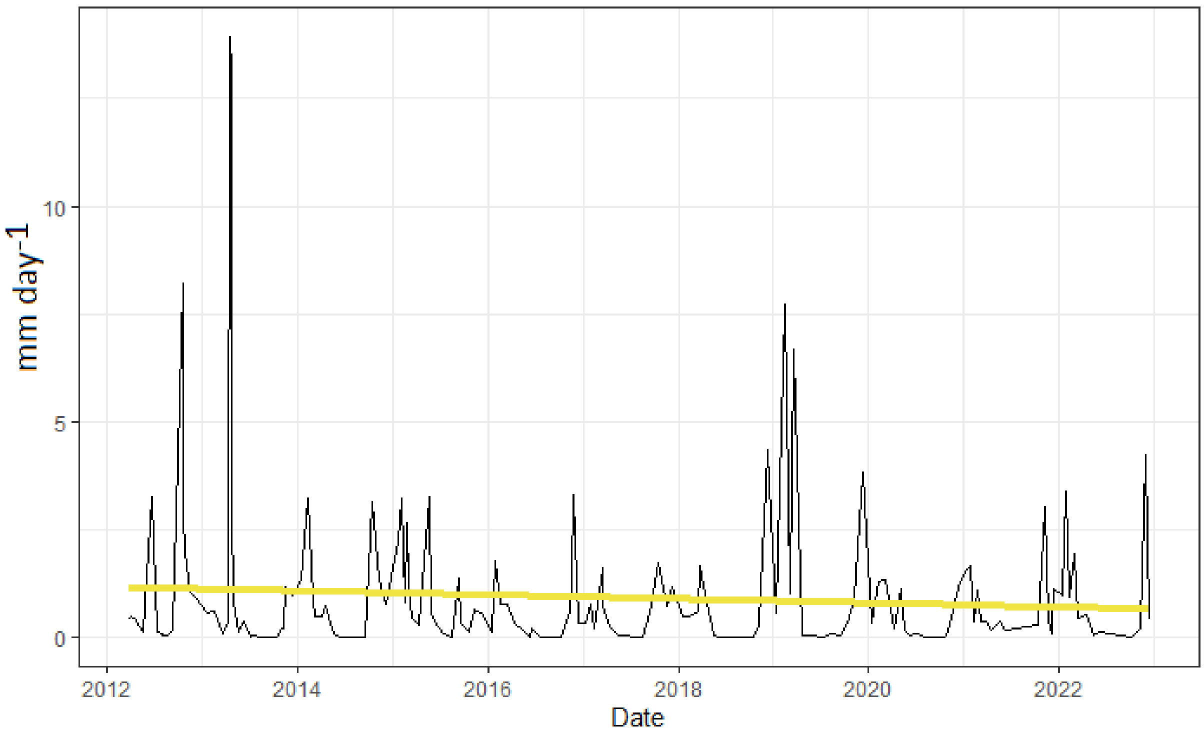

Figure S2. Daily runoff at Stormyra station (black line) and yellow line indicating no significant trend calculated by General Additive Model (GAM).

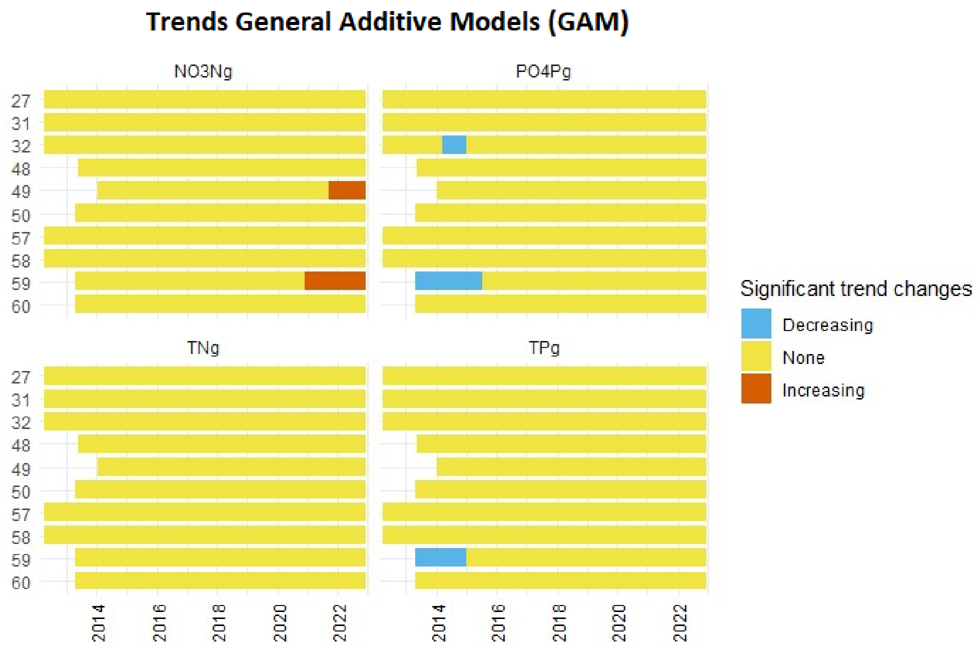

Trends Mann-Kendall

Mann-Kendall statistics on nutrient loads (Theil Sen Slope and p-value (*p < 0.05; **p < 0.01; ***p < 0.001)

ID	NO_3-N	TN	PO_4-P	TP
27	0.08	-0.01	-0.11	-0.06
31	0.11	0.02	0.00	0.00
32	0.00	0.00	-0.05	0.02
48	0.05	0.06	0.13	0.11
49	0.12	-0.02	0.05	0.06
50	-0.06	-0.02	0.00	0.01
57	0.07	0.02	-0.04	-0.01
58	0.04	-0.01	-0.06	-0.03
59	0.01	-0.06	-0.07	-0.05
60	-0.01	-0.06	-0.08	-0.09

Figure S3. Trends in loads of total phosphorus (TP), phosphate-phosphorus (PO_4-P), total nitrogen (TN) and nitrate-N (NO_3-N), as calculated with (top panel) general additive models (GAM) and (bottom panel) Mann-Kendall test for 10 outlet stations. Blue color indicates a period with a significant decreasing trend, yellow no significant trend, and red a significant increasing trend.

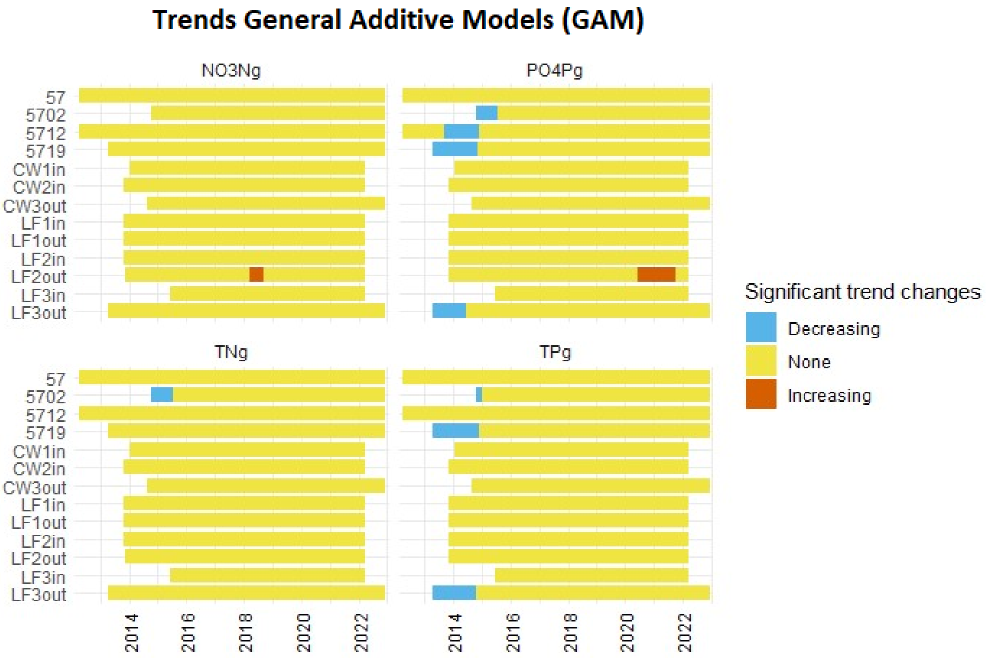

Trends Mann-Kendall

Mann-Kendall statistics on nutrient loads (Theil Sen

Slope and p-value (*p < 0.05; **p < 0.01; ***p < 0.001)

ID	NO_3-N	TN	PO_4-P	TP
57	0.07	0.02	-0.04	-0.01
5702	-0.15	-0.15	0.04	0.02
5712	0.10	0.02	-0.08	-0.06
5719	0.08	0.01	-0.02	0.00
CW1in	0.09	0.07	-0.03	0.00
CW2in	-0.07	-0.06	-0.03	-0.02
CW3out	0.07	0.02	0.06	0.04
LF1in	-0.03	-0.04	-0.03	-0.03
LF1out	-0.03	-0.04	0.06	0.00
LF2in	-0.12	-0.12	-0.02	-0.08
LF2out	-0.10	-0.13	0.14	0.09
LF3in	0.22*	0.12	0.12	0.13
LF3out	0.11	0.06	0.02	0.04

Figure S4. Trends in loads of total phosphorus (TP), phosphate-phosphorus (PO4-P), total nitrogen (TN), and nitrate-nitrogen (NO_3-N) in sub-catchment 57, calculated with (upper panel) general additive models (GAM) and (lower panel) Mann-Kendall test. Blue indicates a period with a significant decreasing trend, yellow no significant trend, and red a significant increasing trend.

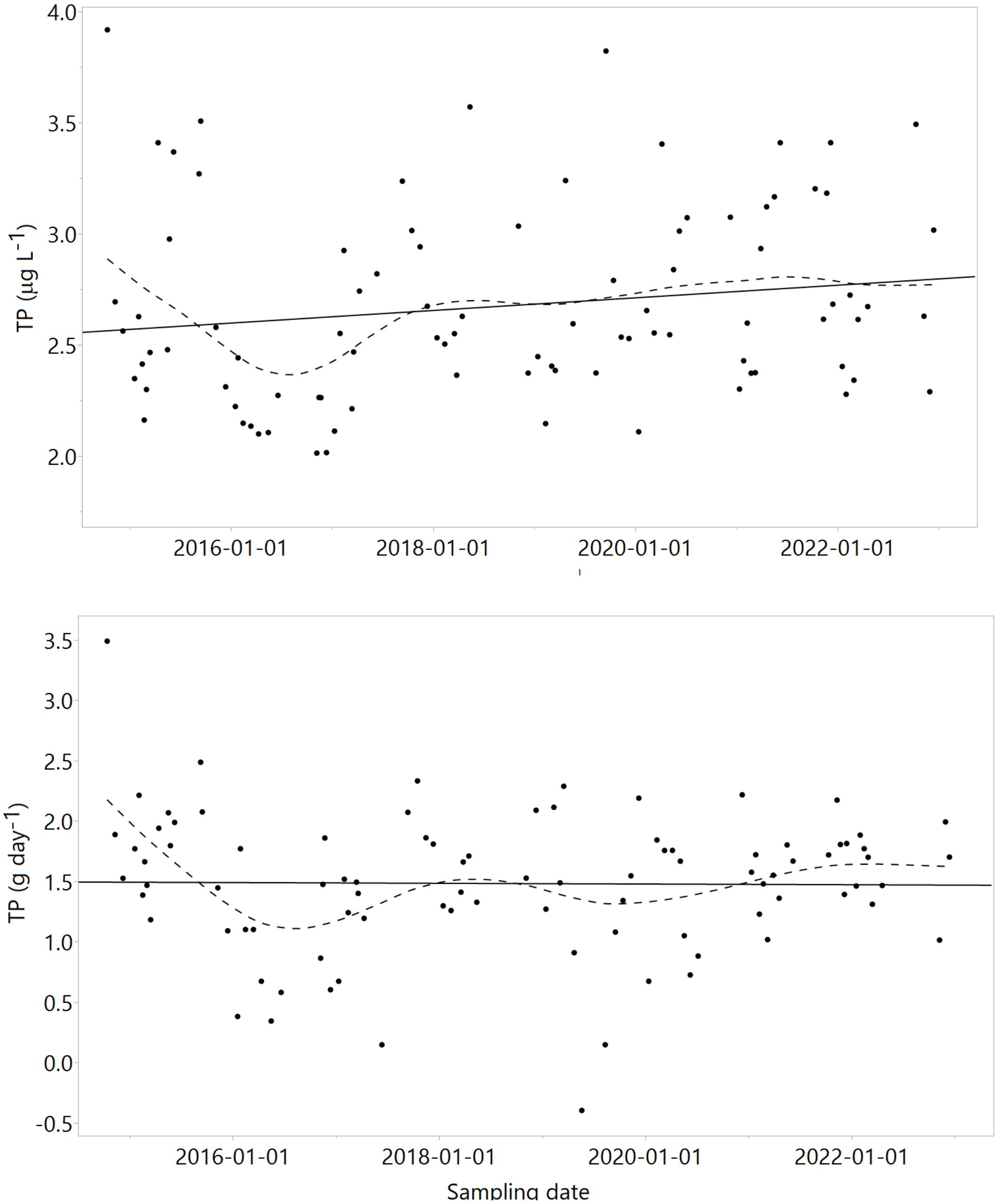

Figure S5. Measured values of total phosphorus concentrations (upper panel) and loads (lower panel) with a linear fit (solid line) and smoother (dotted line) at station 5702. General additive model identified the temporarily decreasing trend in concentrations and loads between 2014 and 2016 (dotted line), while Mann-Kendall test identified the increasing linear trend in concentrations over the whole period (solid line).

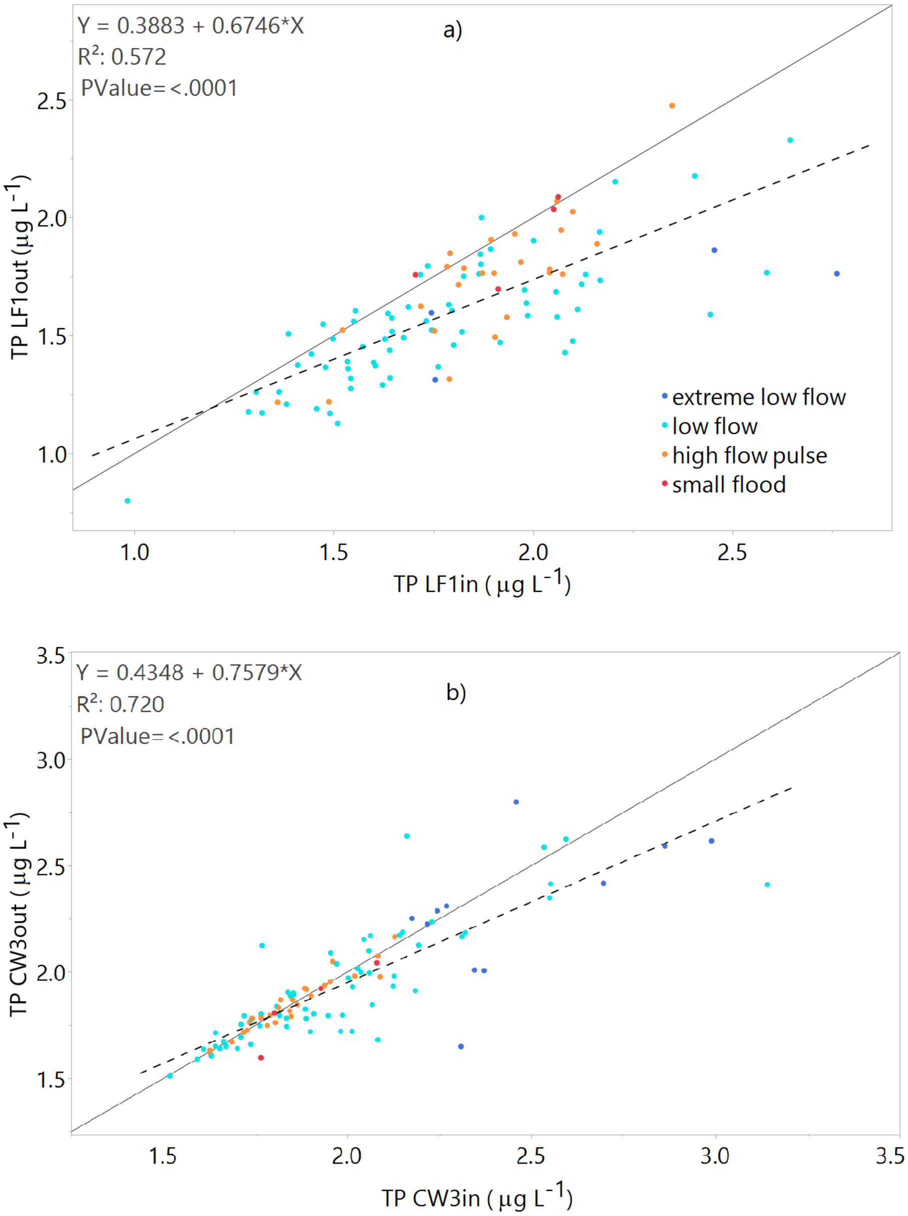

Figure S6. Concentrations of a) TP at the outlet (y-axis) of Lime Filter 1 (LF1) plotted against corresponding concentrations at the inlet of LF1 (x-axis), and b) TP at the outlet (y-axis) of Constructed Wetland 3 (CW3) plotted against corresponding concentrations at the inlet of CW3 (x-axis). The solid line represents the 1:1 line and the dashed line is linear fit. Environmental flow components at the sampling occasion are indicated by color. Note logarithmic axes.

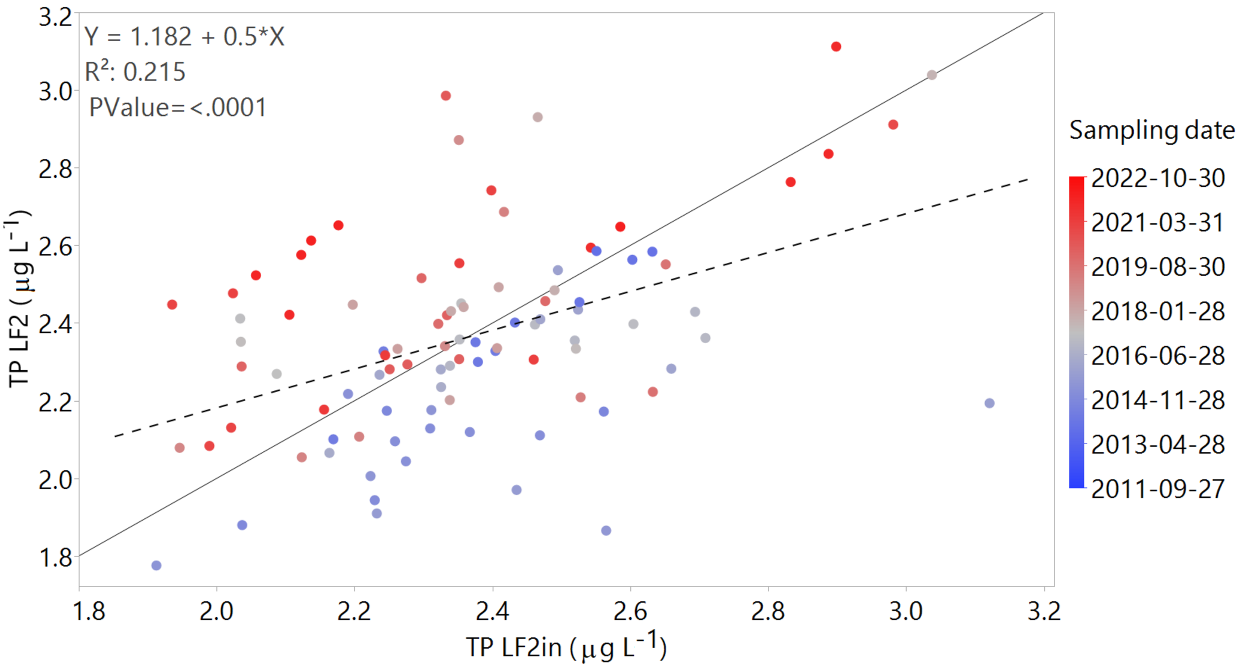

Figure S7. Concentrations of TP at the outlet (y-axis) of Lime Filter 2 (LF2) plotted against corresponding concentrations at the inlet of LF2 (x-axis). The solid line represents the 1:1 line and the dashed line is linear fit. Sampling occasion dates are indicated by color. Note logarithmic axes.

Table S1. Mean values of pH, electrical conductivity, alkalinity, nitrate-N (NO_3-N), total nitrogen (TN), phosphate-phosphorus (PO_4-P), and total phosphorus (TP) at outlet stations in the 10 main sub-catchments calculated for each environmental flow component (EFC). Mean values of different variables per EFC within the same sub-catchment with different letters (A-C) are significantly different ($p < 0.05$) according to Student's t- test.

	**EFC**	**27**		**31**		**32**		**48**		**49**		**50**		**57**		**58**		**59**		**60**	
pH	Extreme low flow	-	-	-	-	-	-	-	-	-	-	7.5	A	7.3	A	7.1	A	-	-	-	-
	Low flow	7.2	A	7.01	A	7.0	A	7.7	A	6.8	A	6.8	B	7.1	B	7.0	A	6.8	A	7.3	A
	High flow pulse	7.0	B	6.9	AB	6.9	A	7.5	B	6.5	B	6.7	B	6.7	C	6.7	B	6.4	B	6.9	B
	Small flood	6.8	C	6.8	B	6.8	A	7.3	B	6.1	B	6.6	B	6.5	D	6.5	B	6.3	C	6.6	C
Electrical conductivity mSm^{-1}	Extreme low flow	-	-	-	-	794	A	-	-	-	-	115	A	72	A	63	A	-	-	-	-
	Low flow	21	A	16	A	37	B	18	A	22	A	34	B	32	B	23	B	15	A	25	A
	High flow pulse	14	B	15	A	23	C	13	B	16	B	23	C	20	C	15	C	11	B	13	B
	Small flood	11	B	13	A	20	C	12	B	16	AB	19	C	16	C	14	C	9	AB	10	C
Alkalinity $meql^{-1}$	Extreme low flow	-	-	-	-	-	-	-		-	-	8.3	A	2.7	A	0.21	A	-	-	-	-
	Low flow	1.2	A	0.5	A	1.5	A	1.5	A	0.7	A	1.0	B	1.2	B	0.9	B	0.4	A	1.5	A
	High flow pulse	0.7	B	0.4	B	1.0	B	1.0	B	0.4	B	0.6	C	0.5	C	0.4	C	0.2	B	0.6	B
	Small flood	0.4	C	0.3	B	1.0	B	0.6	C	0.1	C	0.5	C	0.3	D	0.3	D	0.1	C	0.4	B
NO3-N μgl^{-1}	Extreme low flow	-	-	-	-	3	B	-	-	-	-	2	C	166	C	23	B	-	-	-	-
	Low flow	186	A	195	A	42	B	6	A	48	A	83	B	646	B	62	B	62	B	112	B
	High flow pulse	263	A	214	A	224	A	6	A	66	A	437	A	1349	A	178	A	123	A	275	A
	Small flood	275	A	174	A	234	A	5	A	158	A	550	A	1148	AB	204	A	71	AB	324	A
TN μgl^{-1}	Extreme low flow	-	-	-	-	1585	A	-	-	-	-	50119	A	3802	A	776	C	-	-	-	-
	Low flow	1096	A	891	A	1266	A	479	B	759	A	1862	B	2188	B	912	BC	1023	A	794	B
	High flow pulse	1202	A	871	A	1255	A	589	A	832	A	1660	B	2512	B	1072	A	1047	A	1122	A
	Small flood	1122	A	813	A	12509	A	661	A	871	A	1622	B	2188	B	1072	AB	1000	A	1122	AB
PO4-P μgl^{-1}	Extreme low flow	-	-	-	-	52	A	-	-	-	-	3715	A	234	A	30	A	-	-	-	-
	Low flow	8	A	13	A	19	A	2	A	4	A	19	B	43	B	19	A	3	A	29	A
	High flow pulse	6	AB	9	B	15	A	3	A	4	A	11	C	25	C	12	B	3	A	35	A
	Small flood	4	B	9	AB	20	A	1	A	2	A	11	BC	32	BC	11	B	3	A	34	A
TP μgl^{-1}	Extreme low flow	-	-	-	-	229	A	-	-	-	-	3715	A	380	A	74	A	-	-	-	-
	Low flow	36	A	35	A	54	B	13	B	26	A	63	B	102	B	48	AB	21	A	79	B
	High flow pulse	33	A	30	A	48	B	19	A	26	A	44	C	76	C	42	B	22	A	112	A
	Small flood	32	A	30	A	59	B	18	A	20	A	49	BC	93	BC	40	B	26	A	120	AB

Table S2. Measured data

AMBIO
Supplementary Information
Title: Usual suspects meet mission impossible: Nutrient losses and effects of mitigation measures on a coastal catchment in the Baltic Sea region

Sub-catch	Date	pH	EC (mS/m)	Alkal. (meq/l)	NO3-N (µg/l)	TN (µg/l)	PO4-P (µg/l)	TP (µg/l)
27	3/27/2012	7.4	20.5	1.6	136	740	7	28
27	4/2/2012	7.3	21.2	1.4	143	833	7	30
27	4/10/2012	7.4	21.9	1.5	127	752	6	24
27	6/19/2012	7.1		0.9	58	1045	8	64
27	7/10/2012	7.6	17.8	1.5	40	1231	9	59
27	7/30/2012	7.4	41.8	2.9	430	1616	42	104
27	7/31/2012	7.4	43.1	3.6	218	1228	34	79
27	8/20/2012	7.4		4.5	422	1352	62	167
27	9/10/2012	7.2		0.6	33	834	5	22
27	10/16/2012	6.9		0.7	227	1457	11	83
27	10/18/2012	6.8	9.7	0.6	52	958	9	59
27	10/23/2012	6.8	8.7	0.5	29	917	6	36
27	11/12/2012	7.3		0.8	59	816	7	40
27	12/12/2012	6.9	10.6	0.7	79	742	5	31
27	1/16/2013		10.6					
27	2/11/2013	7.0	12.7	0.9	105	863	7	31
27	4/10/2013	7.1	32.6	1.7	102	1044	16	77
27	4/16/2013	6.7		0.3	305	1011	5	50
27	4/18/2013	6.8	8.1	0.3	259	977	8	55
27	4/22/2013	6.9	7.9	0.4	168	731	4	21
27	4/29/2013	7.1	10.2	0.6	93	801	5	28
27	5/15/2013	7.4	20.5	1.4	57	844	14	41
27	6/10/2013	7.3		4.0	156	925	27	62
27	10/29/2013	6.9	27.7	0.9	648	1969	22	128
27	11/4/2013	7.0	30.4	0.9	1249	2552	18	80
27	11/12/2013	7.0	19.7	0.9	625	1487	7	36
27	12/11/2013	7.0	12.6	0.5	196	893	4	24
27	1/14/2014	6.8	9.6	0.5	150	749	4	21
27	2/11/2014	7.0	10.6	0.4	262	825	4	20
27	3/3/2014	7.1	14.4	0.7	217	755	5	20
27	3/10/2014	7.2	12.1	0.7	138	706	2	17
27	4/1/2014	7.1	12.0	0.7	136	723	4	15
27	4/14/2014	7.3	12.2	0.7	102	800	4	24
27	5/13/2014	7.4	17.8	1.1	115	906	10	44
27	10/13/2014	6.9	16.0	0.6	336	1560	13	45
27	11/10/2014	7.1	14.9	0.8	92	1006	11	30
27	12/8/2014	7.3	18.1	1.1	268	1176	10	82
27	1/19/2015	7.0	10.0	0.4	245	850	6	20
27	2/3/2015	6.8	7.3	0.3	176	744	4	18
27	2/16/2015	6.9	11.0	0.5	156	769	7	16
27	2/23/2015	7.0	12.5	0.6	204	847	3	24
27	3/3/2015	7.1	13.9	0.7	187	874	8	29

27	3/16/2015	7.2	13.6	0.8	173	890	7	25
27	4/14/2015	7.2	14.0	0.9	105	815	8	23
27	5/18/2015	7.1	14.3	0.9	307	1403	7	76
27	5/25/2015	7.4	13.9	0.9	29	842	5	30
27	6/9/2015	7.6	17.9	1.3	59	961	7	32
27	7/14/2015	7.5	21.6	1.7	167	1177	24	52
27	9/9/2015	7.2		1.3	119	1170	13	48
27	9/15/2015	7.3	46.4	3.0	268	1018	10	39
27	10/19/2015	7.2	53.5	4.2	443	1573	39	111
27	11/9/2015	7.2	23.1	1.5	295	2079	0	87
27	12/14/2015	7.0	13.9	0.8	102	899	7	24
27	1/18/2016	6.8	24.6	1.7	67	837	12	33
27	1/28/2016	7.2	17.5	0.9	502	1367	10	51
27	2/15/2016	7.0	14.1	0.7	155	725	4	18
27	3/14/2016	7.2	16.7	0.9	176	750	4	24
27	4/11/2016	7.4	21.0	1.5	102	801	11	27
27	5/16/2016	7.6	30.5	1.9	147	1218	21	61
27	6/20/2016	7.6	30.2	1.4	204	1432	15	69
27	11/7/2016	7.2	37.6	1.3	3594	5213	30	70
27	11/15/2016	6.7	17.5	0.4	1528	2542	6	40
27	11/22/2016	6.8	10.7	0.4	257	1006	3	21
27	12/12/2016	6.7	19.5	0.9	243	914	7	21
27	1/10/2017	6.8	19.2	0.9	206	814	3	18
27	1/30/2017	7.2	17.2	0.9	272	944	6	24
27	2/13/2017	6.7	22.2	1.3	96	897	13	32
27	3/14/2017	7.2	17.9	0.8	284	953	0	21
27	3/20/2017	7.2	15.4	0.8	210	786	5	17
27	4/10/2017	7.4	19.1	1.1	126	805	9	28
27	5/15/2017	7.4	25.3	1.8	109	928	14	44
27	9/12/2017	7.4	71.2	2.9	4546	7856	331	438
27	10/16/2017	6.7	13.1	0.6	67	1189	2	35
27	11/15/2017	7.1	14.0	0.7	91	1030	3	24
27	12/11/2017	6.9	12.3	0.6	107	950	4	22
27	1/15/2018	6.9	13.9	0.7	124	892	5	20
27	2/12/2018	6.8	12.6	0.7	120	821	7	15
27	3/19/2018	6.7	14.9	0.8	152	926	3	19
27	3/27/2018	6.7	15.4	0.8	188	889	5	28
27	4/16/2018	6.8	14.8	0.8	119	747	6	25
27	5/14/2018	7.4	29.0	2.4	187	1148	14	39
27	11/5/2018	7.2	61.2	1.6	4459	6236	47	96
27	12/10/2018	6.9	19.4	0.7	1762	2732	9	33
27	1/14/2019	6.9	20.2	0.9	336	1141	4	21
27	2/11/2019	6.5	15.5	0.4	521	1397	9	29
27	3/5/2019	7.1	17.8	0.7	204	940	4	24
27	3/18/2019	7.0	11.7	0.5	230	859	5	24
27	4/24/2019	7.4	27.5	1.8	92	940	13	42
27	5/21/2019	7.4	27.6	2.0	135	1499	23	76
27	8/12/2019	7.1	46.8	1.5	1463	2691	37	109
27	9/17/2019	7.6	31.2	2.1	281	1619	12	50
27	10/14/2019	7.2	31.9	1.6	361	1748	27	151

27	11/11/2019	7.1	16.9	1.0	154	867	9	35
27	12/9/2019	6.9	11.9	0.6	119	1041	7	32
27	1/13/2020	7.1	15.2	1.0	100	701	4	24
27	2/11/2020	7.1	17.1	1.0	366	1350	17	59
27	3/9/2020	7.0	10.6	0.5	128	957	3	24
27	4/6/2020	7.3	18.5	1.2	130	904	6	26
27	5/4/2020	7.5	18.5	1.2	172	1052	7	50
27	5/18/2020	7.7	22.9	1.6	87	1033	9	33
27	6/8/2020	7.6	49.9	3.5	436	1504	22	53
27	12/9/2020	7.2		1.4	5495	5939	11	50
27	1/11/2021		15.4		400	1015	2	18
27	1/26/2021		12.2		270	924	2	16
27	2/8/2021		15.5		303	974	5	19
27	2/22/2021		22.0		451	1150	7	27
27	3/9/2021		17.2		280	897	3	22
27	3/29/2021		19.0		181	812	5	23
27	4/19/2021		27.7		141	1366	30	58
27	5/17/2021		24.8		238	1261	8	68
27	6/7/2021		16.0		104	1075	2	40
27	11/10/2021		26.1		1705	2859	8	44
27	11/22/2021		26.5		193	1229	5	29
27	12/6/2021		36.6		131	993	7	27
27	12/14/2021		19.7		547	1432	5	38
27	1/17/2022		18.6		313	1201	5	22
27	1/31/2022		9.1		562	1380	5	30
27	2/14/2022		20.5		243	1023	6	35
27	2/28/2022		11.6		218	854	1	16
27	3/15/2022		16.3		183	780	2	16
27	4/19/2022		20.7		163	908	3	26
27	5/16/2022		28.0		199	1347		
27	6/7/2022		22.9		132	1191		
27	11/29/2022		23.6		2411	3679	6	34
27	12/13/2022		20.4		589	1482	2	17
31	3/27/2012	7.2	19.9	0.9	111	493	13	27
31	4/2/2012	7.2	21.1	0.9	159	621	10	26
31	4/10/2012	7.3	19.7	1.0	113	508	11	22
31	5/15/2012	6.9		0.3	65	407	2	5
31	6/19/2012	7.1		0.7	84	882	19	69
31	7/10/2012	7.0	9.4	0.5	118	864	11	38
31	7/30/2012	7.1	17.6	1.0	179	1100	22	68
31	7/31/2012	7.0	18.7	1.2	33	918	31	61
31	8/2/2012	6.8	13.4	0.7	43	887	14	43
31	8/20/2012	6.7		1.1	10	851	33	101
31	9/10/2012	6.9		0.6	33	905	138	230
31	10/16/2012	6.9		0.5	116	1054	15	67
31	10/18/2012	7.0	11.9	0.6	64	691	13	45
31	10/23/2012	7.0	10.8	0.5	39	574	9	26
31	11/12/2012	7.3		0.6	69	546	8	30
31	12/12/2012	7.0	11.4	0.6	80	553	6	23
31	1/16/2013	7.0	11.4	0.7	104	743	9	32

31	2/11/2013	7.0	11.6	0.6	124	579	9	24
31	3/18/2013	6.9		1.6	115	777	35	84
31	4/10/2013	7.1	26.6	0.9	221	867	19	60
31	4/16/2013	6.7		0.3	189	768	10	38
31	4/18/2013	6.8	11.2	0.3	194	842	14	47
31	4/22/2013	6.9	10.1	0.3	136	558	4	21
31	4/29/2013	7.0	10.1	0.4	120	525	9	23
31	5/15/2013	6.9	12.7	0.6	34	585	37	74
31	6/10/2013	6.9		1.0	60	936	113	204
31	10/29/2013	6.4	25.6	0.3	2461	3499	14	64
31	11/4/2013	6.6	23.8	0.4	1843	2828	21	59
31	11/12/2013	6.6	20.7	0.5	801	1458	11	34
31	12/11/2013	6.9	12.6	0.3	285	778	6	20
31	1/14/2014	6.7	8.8	0.3	149	558	3	13
31	2/11/2014	6.9	11.3	0.3	254	621	4	16
31	3/3/2014	7.0	14.8	0.4	330	738	5	17
31	3/10/2014	7.0	10.8	0.4	143	514	2	12
31	4/1/2014	7.0	10.6	0.4	134	485	4	9
31	4/14/2014	7.1	10.5	0.4	95	484	2	17
31	5/13/2014	7.1	18.9	0.7	110	664	12	35
31	6/10/2014	6.6	43.3	2.2	59	1663	106	151
31	10/13/2014	6.8	19.5	0.4	293	1282	21	44
31	11/10/2014	6.2	15.3	0.4	137	781	8	27
31	12/8/2014	7.1	16.7	0.5	282	856	7	27
31	1/19/2015	7.0	10.9	0.3	210	601	6	13
31	2/3/2015	6.9	8.3	0.3	148	593	3	16
31	2/16/2015	6.9	11.3	0.4	190	627	4	23
31	2/23/2015	7.0	13.8	0.4	262	695	9	21
31	3/3/2015	7.0	12.1	0.3	191	624	4	13
31	3/16/2015	7.0	10.2	0.3	172	608	3	11
31	4/14/2015	7.0	9.9	0.4	110	527	3	10
31	5/18/2015	7.0	17.8	0.6	333	1198	19	73
31	5/25/2015	7.1	14.5	0.7	88	856	26	46
31	6/9/2015	7.1	9.6	0.4	107	813	6	22
31	7/14/2015	7.0	18.0	1.1	255	1111	47	65
31	8/18/2015	6.8	13.7	0.7	1242	1800	24	55
31	9/9/2015	6.9		0.7	176	1215	27	50
31	9/15/2015	6.9	9.7	0.5	574	1454	32	63
31	10/19/2015	6.7	9.8	0.5	283	914	27	49
31	11/9/2015	7.1	16.4	0.6	439	1482	28	70
31	12/14/2015	7.1	11.0	0.4	153	741	15	29
31	1/18/2016	6.7	12.7	0.5	210	1076	37	53
31	1/28/2016	7.0	19.8	0.5	425	1221	20	56
31	2/15/2016	7.0	13.8	0.4	189	726	12	24
31	3/14/2016	7.1	13.6	0.5	221	740	18	19
31	4/11/2016	7.1	12.7	0.4	202	791	29	40
31	5/16/2016	7.1	18.9	0.8	251	1045	55	80
31	6/20/2016	7.0	21.7	0.6	558	1512	45	95
31	11/7/2016	6.6	19.5	0.3	928	1759	29	62
31	11/15/2016	6.6	19.9	0.3	391	1201	13	43

31	11/22/2016	6.7	11.8	0.2	126	753	12	26
31	12/12/2016	7.0	13.7	0.3	187	692	11	28
31	1/10/2017	7.1	15.1	0.3	246	759	10	24
31	1/30/2017	7.1	15.4	0.3	243	736	14	30
31	2/13/2017	7.0	15.3	0.5	368	1098	35	51
31	3/14/2017	7.0	19.4	0.4	217	682	2	19
31	3/20/2017	7.2	14.2	0.4	194	648	12	22
31	4/10/2017	7.1	15.2	0.4	213	808	18	30
31	5/15/2017	7.2	20.1	0.7	896	1770	51	92
31	6/12/2017	7.1	45.7	1.7	113	2041	94	206
31	10/16/2017	7.0	14.8	0.4	103	1031	9	31
31	11/15/2017	7.2	12.0	0.3	110	769	10	23
31	12/11/2017	7.2	12.9	0.3	129	690	11	24
31	1/15/2018	7.3	11.2	0.3	169	649	8	16
31	2/12/2018	7.8	10.8	0.3	162	543	6	9
31	3/19/2018	7.2	15.7	0.4	181	688	6	20
31	3/27/2018	6.9	16.7	0.5	210	644	10	23
31	4/16/2018	7.2	14.1	0.4	153	538	8	21
31	5/14/2018	7.6	13.4	0.5	91	630	9	25
31	11/5/2018	6.7	49.4	0.4	6813	7504	9	46
31	12/10/2018	6.9	24.6	0.3	549	1327	10	28
31	1/14/2019	7.0	21.4	0.3	359	931	5	19
31	2/11/2019	6.6	14.3	0.2	282	877	6	23
31	3/5/2019	7.0	21.3	0.3	231	773	5	17
31	3/18/2019	6.9	13.2	0.2	185	635	6	19
31	4/24/2019	7.0	18.1	0.5	64	591	4	22
31	5/21/2019	7.1	39.8	1.1	45	722	4	41
31	8/12/2019	7.0	24.6	0.7	467	2193	19	79
31	10/14/2019	7.0	27.2	0.9	231	1551	24	119
31	11/11/2019	7.1	21.0	0.5	122	724	7	30
31	12/9/2019	7.2	13.9	0.4	129	748	10	26
31	1/13/2020	7.2	11.6	0.4	184	713	7	21
31	2/11/2020	7.3	18.6	0.6	352	1235	20	60
31	3/9/2020	7.3	11.0	0.3	130	607	4	16
31	4/6/2020	7.3	11.4	0.4	158	809	9	21
31	5/4/2020	7.2	19.1	0.6	158	838	25	56
31	5/18/2020	7.2	15.5	0.7	168	804	18	36
31	6/8/2020	7.0	22.6	1.0	1165	2006	39	113
31	12/9/2020	6.6		0.4	1729	2601	8	38
31	1/11/2021		11.9		199	703	5	15
31	1/26/2021		10.3		164	671	2	12
31	2/8/2021		10.1		241	753	9	17
31	2/22/2021		18.8		534	1150	21	37
31	3/9/2021		11.1		218	739	4	17
31	3/29/2021		11.7		182	639	8	20
31	4/19/2021		17.1		315	1109	24	43
31	5/17/2021		21.6		207	993	16	56
31	6/7/2021		12.8		215	1000	15	48
31	10/11/2021		25.3		422	1368	26	79
31	11/10/2021		21.4		353	1362	19	56

31	11/22/2021		12.6		236	1157	16	38
31	12/6/2021		16.5		553	1625	30	56
31	12/14/2021		15.9		413	1195	10	38
31	1/17/2022		15.2		225	786	11	28
31	1/31/2022		60.7		332	941	13	31
31	2/14/2022		16.5		180	677	9	23
31	2/28/2022		12.8		140	585	8	21
31	3/15/2022		12.9		155	636	16	27
31	4/19/2022		15.7		180	721	16	31
31	5/16/2022		24.8		652	1321		
31	6/7/2022		18.5		173	962		
31	11/8/2022		33.8		609	1525	32	70
31	11/29/2022		19.7		382	1152	8	34
31	12/13/2022		17.4		243	830	7	21
32	3/27/2012	7.1	32.9	1.8	114	891	17	38
32	4/2/2012	7.0	29.2	1.5	170	894	12	32
32	4/10/2012	7.1	31.6	1.8	86	831	13	32
32	5/15/2012	6.1		0.2	0	1085	5	20
32	6/19/2012	7.0		1.1	54	1190	35	93
32	7/10/2012	7.0	31.3	2.5	0	1926	35	86
32	7/30/2012	6.9	25.0	1.6	33	1734	59	133
32	7/31/2012	7.0	26.8	1.8	0	1693	44	96
32	8/2/2012	7.2	34.5	2.6	0	1710	37	74
32	8/20/2012	7.1		3.5	4	1994	63	126
32	9/10/2012	6.9		1.8	4	1753	48	90
32	10/16/2012	6.9		0.9	223	1669	54	143
32	10/18/2012	7.0	17.8	1.2	63	1290	27	83
32	10/23/2012	6.9	17.8	1.2	60	1189	30	65
32	11/12/2012	7.2		1.6	98	444	1	6
32	12/12/2012	7.0	20.4	1.5	139	998	22	48
32	1/16/2013	6.9	22.8	1.6	104	972	26	48
32	2/11/2013	6.8	24.3	1.6	123	1033	30	56
32	3/18/2013	6.9		2.6	76	1289	32	60
32	4/10/2013	7.0	34.3	1.9	261	1200	42	86
32	4/16/2013	6.9		0.6	412	1202	23	71
32	4/18/2013	7.0	14.5	0.7	329	1183	22	77
32	4/22/2013	7.0	17.5	1.0	220	1027	22	63
32	4/29/2013	7.1	24.3	1.5	102	1016	25	54
32	5/15/2013	7.2	38.9	2.3	34	1524	57	95
32	6/10/2013	7.1		2.9	3	1749	66	111
32	10/29/2013	6.2	89.6	0.4	2073	3417	28	98
32	11/4/2013	6.3	58.7	0.6	272	1496	26	70
32	11/12/2013	6.5	29.4	0.7	108	1007	19	46
32	12/11/2013	7.0	24.1	0.9	141	838	13	29
32	1/14/2014	6.7	19.6	0.9	157	957	17	36
32	2/11/2014	6.9	18.1	0.8	237	951	14	37
32	3/3/2014	6.9	24.2	1.1	152	823	12	29
32	3/10/2014	7.0	24.0	1.2	101	808	7	30
32	4/1/2014	7.0	23.4	1.2	87	935	15	40
32	4/14/2014	7.2	22.1	1.2	36	813	9	34

32	5/13/2014	7.1	26.0	1.5	44	916	14	48
32	6/10/2014	7.3	82.7	5.3	0	822	16	70
32	10/13/2014	6.8	24.1	0.8	91	1308	25	63
32	11/10/2014	6.7	23.3	1.0	63	1294	20	49
32	12/8/2014	7.0	26.1	1.2	206	1208	14	50
32	1/19/2015	7.0	18.0	0.9	274	991	13	37
32	2/3/2015	7.0	16.1	0.7	267	1059	14	41
32	2/16/2015	6.8	22.5	1.0	200	924	13	29
32	2/23/2015	7.1	11.5	0.9	274	993	11	34
32	3/3/2015	7.0	24.8	1.2	177	958	15	37
32	3/16/2015	6.9	24.8	1.3	204	1021	17	37
32	4/14/2015	6.9	26.1	1.7	68	1038	18	47
32	5/18/2015	7.1	20.2	1.1	255	1317	26	87
32	5/25/2015	7.1	23.7	1.5	5	1248	17	51
32	6/9/2015	7.1	24.8	1.7	1	1393	20	61
32	7/14/2015	6.8	31.2	2.0	16	1699	46	100
32	9/9/2015	6.8		1.2	20	1466	29	73
32	9/15/2015	6.9	30.9	1.4	10	1736	46	97
32	10/19/2015	7.0	47.0	3.3	8	1869	50	92
32	11/9/2015	7.0	33.8	1.9	423	1917	31	97
32	12/14/2015	7.0	24.9	1.4	123	1124	12	39
32	1/18/2016	6.7	36.9	2.2	46	1101	21	39
32	1/28/2016	7.3	26.9	1.2	416	1326	35	76
32	2/15/2016	7.0	27.8	1.4	217	1056	11	35
32	3/14/2016	7.2	27.2	1.4	223	924	14	30
32	4/11/2016	7.1	33.9	1.9	130	969	15	35
32	5/16/2016	7.2	43.2	2.4	96	1486	38	80
32	6/20/2016	6.9	34.6	1.1	60	1296	17	75
32	11/7/2016	6.5	57.1	0.4	1632	3192	9	45
32	11/15/2016	6.8	20.1	0.4	528	1409	36	75
32	11/22/2016	6.7	23.8	0.6	175	1191	15	42
32	12/12/2016	7.0	36.0	1.0	177	1007	7	28
32	1/10/2017	6.7	39.9	1.1	107	914	2	26
32	1/30/2017	6.9	29.0	1.0	138	827	2	22
32	2/13/2017	6.8	37.6	1.5	68	929	8	28
32	3/14/2017	7.0	25.8	1.0	224	903	3	26
32	3/20/2017	7.0	28.9	1.2	123	837	4	23
32	4/10/2017	7.0	34.3	1.5	25	874	7	28
32	5/15/2017	7.4	47.3	2.1	6	1240	17	58
32	6/12/2017	7.4	84.2	4.4	0	1126	27	80
32	9/12/2017	6.7	140.4	0.6	152	2165	30	71
32	10/16/2017	6.8	17.8	1.0	43	1614	23	66
32	11/15/2017	6.9	26.4	1.2	56	1404	18	49
32	12/11/2017	7.0	25.5	1.2	117	1205	13	41
32	1/15/2018	7.0	26.2	1.3	93	1120	19	46
32	2/12/2018	7.2	27.0	1.3	354	1048	28	39
32	3/19/2018	6.9	28.5	1.3	100	996	14	38
32	3/27/2018	6.8	29.5	1.0	188	864	12	33
32	4/16/2018	7.0	29.2	1.3	58	864	13	37
32	5/14/2018	7.1	39.9	2.2	4	1690	44	91

32	11/5/2018	6.2	215.0	0.3	2083	2791	5	37
32	12/10/2018	6.8	28.2	0.6	894	1680	17	45
32	1/14/2019	7.1	37.8	0.8	206	1048	8	28
32	2/11/2019		20.5					
32	3/5/2019	6.9	29.7	0.9	144	877	5	25
32	3/18/2019	7.0	18.9	0.7	273	923	9	33
32	4/24/2019	6.9	41.9	1.3	3	1094	24	58
32	5/21/2019	7.1	48.7	1.7	4	1551	41	93
32	5/27/2019	7.1	60.2	1.8	7	1754	33	93
32	10/14/2019	6.6	55.5	1.5	38	1982	67	132
32	11/11/2019	7.0	29.1	1.4	113	984	22	61
32	12/9/2019	7.0	22.0	1.1	198	1234	21	59
32	1/13/2020	6.9	29.4	1.6	33	1270	55	103
32	2/11/2020	7.2	29.4	1.5	444	1501	26	72
32	3/9/2020	7.0	29.5	1.1	148	1188	21	52
32	4/6/2020	7.0	34.5	1.9	10	1338	52	93
32	5/4/2020	7.2	25.9	1.5	150	1197	19	69
32	5/18/2020	7.2	34.6	2.1	1	1598	48	100
32	6/8/2020	7.2	47.5	3.0	22	2168	86	146
32	7/6/2020	7.4	81.9	3.3	69	1504	37	88
32	12/9/2020	6.1		0.3	4753	5760	6	51
32	1/11/2021		33.4		330	1216	11	35
32	1/26/2021		27.2		282	1249	7	34
32	2/8/2021		35.9		117	1070	10	34
32	2/22/2021		39.2		416	1227	10	36
32	3/9/2021		32.0		117	930	6	30
32	3/29/2021		33.0		26	838	10	35
32	4/19/2021		40.3		10	1035	13	46
32	5/17/2021		34.5		11	1347	17	69
32	6/7/2021		31.2		3	1594	41	115
32	10/11/2021		76.1		3	1852	48	104
32	11/10/2021		32.3		980	2172	29	75
32	11/22/2021		39.4		38	1602	33	70
32	12/6/2021		50.5		6	1580	27	63
32	12/14/2021		29.0		402	1509	14	48
32	1/17/2022		30.1		296	1196	13	34
32	1/31/2022		27.0		832	1749	23	59
32	2/14/2022		31.2		178	1055	14	40
32	2/28/2022		22.7		281	1067	10	32
32	3/15/2022		32.0		58	1066	15	40
32	4/19/2022		32.3		13	1074	9	40
32	5/16/2022		44.7		8	1903		
32	6/7/2022		34.4		6	1876		
32	10/10/2022		800.0		3	1575	53	230
32	11/8/2022		626.0		486	2016	29	67
32	11/29/2022		25.2		934	2052	19	50
32	12/13/2022		42.1		182	1478	9	45
48	1/14/2014	7.3	12.4	0.9	2	492	0	14
48	2/11/2014	7.4	10.9	1.0	6	489	7	15
48	3/3/2014	7.6	14.8	1.3	8	449	1	15

48	3/10/2014	7.7	15.8	1.4	3	395	0	9
48	4/1/2014	7.7	15.9	1.4	3	412	4	8
48	4/14/2014	7.8	15.9	1.4	0	421	0	11
48	5/13/2014	7.8	20.2	1.9	3	561	3	20
48	10/13/2014	7.5	12.2	0.8	5	732	4	19
48	11/10/2014	7.7	16.2	1.3	1	536	2	13
48	12/8/2014	7.9	18.6	1.7	5	522	1	14
48	1/19/2015	7.5	11.9	0.8	7	461	2	12
48	2/3/2015	7.3	9.2	0.7	0	537	2	14
48	2/16/2015	7.5	14.4	1.1	5	465	3	9
48	2/23/2015	7.5	14.0	1.1	0	515	0	17
48	3/3/2015	7.6	34.9	2.9	4	452	4	11
48	3/16/2015	7.6	17.9	1.6	7	382	2	9
48	4/14/2015	7.6	17.2	1.6	12	491	6	15
48	5/18/2015	7.4	9.7	0.7	3	784	2	47
48	5/25/2015	7.7	17.9	1.7	3	551	4	12
48	6/9/2015	7.8	19.6	2.0	57	552	2	39
48	9/9/2015	7.5		1.4	0	654	1	16
48	9/15/2015	7.7	20.1	1.8	14	534	0	17
48	11/9/2015	7.8	25.8	2.3	12	829	3	40
48	12/14/2015	7.8	19.7	1.7	7	487	1	9
48	1/28/2016	7.4	11.5	1.0	16	665	2	37
48	2/15/2016	7.7	19.3	1.6	6	415	1	9
48	3/14/2016	7.6	20.3	1.9	6	423	1	13
48	4/11/2016	7.8	24.2	2.3	10	399	1	6
48	6/20/2016	7.7	17.7	1.4	6	740	3	36
48	11/7/2016	7.5	13.7	0.7	4	570	3	20
48	11/15/2016	7.1	8.5	0.4	14	789	5	31
48	11/22/2016	7.2	11.5	0.7	0	619	0	14
48	12/12/2016	7.7	17.8	1.4	9	412	1	7
48	1/10/2017	7.8	21.1	1.6	15	376	0	8
48	1/30/2017	7.7	18.0	1.5	7	380	6	9
48	3/14/2017	7.6		1.0	2	477	1	12
48	3/20/2017	7.7	17.4	1.4	1	445	6	18
48	4/10/2017	7.8	20.0	1.8	6	391	1	6
48	10/16/2017	7.7	14.4	1.1	1	819	0	20
48	11/15/2017	7.3	16.4	1.4	1	596	1	15
48	12/11/2017	7.7	15.1	1.2	4	507	1	13
48	1/15/2018	7.5	15.0	1.2	2	500	1	9
48	2/12/2018	7.6	15.8	1.3	22	503	1	21
48	3/19/2018	7.5	15.6	1.4	3	471	2	13
48	3/27/2018	7.5	12.5	1.0	5	468	3	18
48	4/16/2018	7.6	15.5	1.3	10	502	3	16
48	12/10/2018	7.4	14.0	0.8	23	637	2	18
48	1/14/2019	7.7	15.4	1.1	8	433	2	8
48	2/11/2019	7.0	6.5	0.3	23	662	3	23
48	3/5/2019	7.5	12.6	0.9	7	484	3	13
48	3/18/2019	7.3	9.4	0.6	5	545	2	21
48	10/14/2019	7.6	20.2	1.6	5	706	3	34
48	11/11/2019	7.7	20.9	1.7	5	422	2	20

48	12/9/2019	7.5	12.5	1.0	9	766	3	27
48	1/13/2020	7.9	20.8	1.9	11	347	2	11
48	2/11/2020	7.7	14.3	1.2	28	789	5	43
48	3/9/2020	7.5	14.1	1.2	3	565	3	18
48	5/4/2020	7.7	15.6	1.3	3	672	4	39
48	5/18/2020	8.0	23.9	2.2	13	439	5	14
48	1/11/2021		15.0		14	485	3	12
48	1/26/2021		13.0		6	537	2	12
48	2/8/2021		17.2		12	483	2	8
48	2/22/2021		13.6		40	578	4	24
48	3/9/2021		15.6		6	425	0	6
48	3/29/2021		17.7		6	350	1	10
48	12/14/2021		16.9		64	654	2	23
48	1/17/2022		17.2		9	486	2	16
48	1/31/2022		11.0		7	695	4	25
48	2/14/2022		8.1		5	516	3	15
48	2/28/2022		11.8		3	551	3	15
48	3/15/2022		16.5		7	392	1	6
48	4/19/2022		21.0		10	542	1	16
48	6/7/2022		21.6		8	589		
48	11/29/2022		15.0		186	942	4	26
49	5/15/2013	7.2	65.2	3.9	18	753	5	29
49	6/10/2013	6.9		1.5	703	1539	20	88
49	10/29/2013	5.5	12.5	0.1	132	1311	12	82
49	11/4/2013	6.0	12.6	0.2	137	1038	8	56
49	11/12/2013	5.9	10.8	0.1	52	872	7	33
49	12/11/2013	6.4	12.1	0.4	236	816	12	38
49	1/14/2014	6.7	23.0	1.2	122	893	0	17
49	2/11/2014	6.7	16.4	1.0	89	763	9	24
49	3/3/2014	6.8	22.6	1.1	244	807	5	25
49	3/10/2014	7.0	27.9	1.5	15	623	0	17
49	4/1/2014	7.0	30.3	1.8	4	579	4	14
49	4/14/2014	7.1	24.5	1.3	8	651	1	14
49	5/13/2014	7.0	17.4	0.9	7	851	3	42
49	10/13/2014	5.6	23.0	0.1	96	1130	6	42
49	11/10/2014	6.7	46.2	0.6	119	917	5	29
49	12/8/2014	6.8	38.5	0.6	206	1022	3	38
49	1/19/2015	6.9	24.6	1.1	35	714	3	17
49	2/3/2015	6.6	23.8	0.8	79	754	3	19
49	2/16/2015	6.8	31.5	1.4	24	682	4	16
49	2/23/2015	6.6	24.9	0.7	78	724	0	16
49	3/3/2015	6.8	17.3	1.0	151	664	3	15
49	3/16/2015	7.2	37.2	1.7	4	655	3	13
49	4/14/2015	6.9	38.8	2.2	0	630	1	16
49	5/18/2015	6.3	11.5	0.4	39	909	1	48
49	5/25/2015	7.0	17.4	1.6	1	733	2	13
49	9/9/2015	6.2		0.2	2	1058	3	34
49	9/15/2015	6.4	11.0	0.3	0	1066	1	41
49	11/9/2015	6.4	18.9	0.2	118	971	7	52
49	12/14/2015	7.0	33.1	1.2	92	846	3	20

49	1/28/2016	6.2	9.0	0.1	74	841	13	59
49	2/15/2016	6.7	27.3	1.2	134	805	3	21
49	3/14/2016	6.9	40.1	1.4	292	816	2	19
49	4/11/2016	7.1	38.1	1.4	53	660	2	19
49	6/20/2016	6.0	18.2	0.1	56	1050	5	54
49	11/7/2016	6.0	10.3	0.1	111	686	3	21
49	11/15/2016	5.6	14.2	0.1	191	839	4	24
49	11/22/2016	6.2	20.3	0.2	204	877	2	16
49	12/12/2016	7.1	19.0	0.6	72	583	2	19
49	1/10/2017	6.9	17.5	0.7	58	619	2	45
49	1/30/2017	7.0	19.9	0.8	27	506	3	13
49	3/14/2017	6.7	14.1	0.5	68	649	4	24
49	3/20/2017	7.0	29.2	1.0	305	769	4	17
49	4/10/2017	7.3	24.1	1.1	3	635	7	23
49	10/16/2017	6.5	12.1	0.2	149	973	1	20
49	11/15/2017	6.9	15.5	1.3	16	785	2	23
49	12/11/2017	7.0	20.7	0.8	86	802	3	19
49	1/15/2018	7.1	26.3	1.4	38	678	3	20
49	2/12/2018	7.2	23.2	1.4	60	641	4	21
49	3/19/2018	6.9	20.0	1.2	11	530	3	13
49	3/27/2018	6.8	13.9	0.7	51	600	3	18
49	4/16/2018	7.2	29.5	1.3	83	621	4	19
49	12/10/2018	6.3	14.9	0.1	190	921	3	21
49	1/14/2019	6.9	13.8	0.5	7	615	3	18
49	2/11/2019	6.3	8.7	0.2	87	779	5	29
49	3/5/2019	6.6	13.1	0.4	61	682	4	20
49	3/18/2019	6.5	15.4	0.4	151	735	3	20
49	10/14/2019	6.9	11.2	0.2	40	630	7	105
49	11/11/2019	6.5	23.9	0.2	297	891	7	31
49	12/9/2019	6.5	17.5	0.4	147	1084	5	32
49	1/13/2020	7.3	30.6	1.3	141	646	4	22
49	2/11/2020	6.9	17.4	0.5	197	1004	6	39
49	3/9/2020	7.0	24.0	1.0	77	759	4	20
49	5/4/2020	6.7	11.9	0.5	53	849	5	39
49	5/18/2020	7.5	24.4	1.3	0	627	3	20
49	1/11/2021		20.5		108	835	5	21
49	1/26/2021		19.3		52	733	4	18
49	2/8/2021		17.0		14	612	5	18
49	2/22/2021		34.0		210	955	7	26
49	3/9/2021		28.1		72	616	3	21
49	11/10/2021		8.9		122	1169	15	48
49	12/14/2021		6.7		12	823	4	32
49	1/17/2022		7.6		18	676	6	30
49	1/31/2022		7.5		7	870	5	28
49	2/14/2022		11.7		62	654	24	66
49	2/28/2022		16.1		31	701	5	23
49	4/19/2022		22.1		63	804	4	37
49	6/7/2022		207.0		1018	1862		
49	11/29/2022		24.6		159	918	3	19
50	4/16/2013	6.7		0.4	650	1398	10	59

50	4/18/2013	6.6	11.4	0.5	548	1363	10	47
50	4/22/2013	6.6	14.1	0.6	472	1348	8	42
50	4/29/2013	6.7	16.3	0.8	127	1260	22	55
50	5/15/2013	6.9	29.4	1.5	82	2746	111	164
50	6/10/2013	6.8		1.7	48	1404	78	120
50	10/29/2013	6.1	122.0	0.4	1216	2674	24	133
50	11/4/2013	6.1	109.8	0.5	268	1577	13	83
50	11/12/2013	6.5	37.8	0.6	214	1176	10	49
50	12/11/2013	6.4	21.1	0.4	1301	2148	5	22
50	1/14/2014	6.6	18.1	0.7	884	1733	2	24
50	2/11/2014	6.6	15.3	0.6	509	1283	10	24
50	3/3/2014	6.6	21.4	0.7	257	1113	5	24
50	3/10/2014	6.8	19.3	0.7	181	1032	2	26
50	4/1/2014	6.8	18.6	0.8	229	1097	8	24
50	4/14/2014	6.9	17.2	0.7	134	1017	7	29
50	5/13/2014	6.9	24.5	1.0	64	1241	20	59
50	10/13/2014	6.7	25.8	0.7	1283	2022	13	43
50	11/10/2014	6.8	20.3	0.8	244	1509	17	47
50	12/8/2014	7.0	22.2	0.9	238	1410	14	48
50	1/19/2015	6.6	18.9	0.5	991	1941	7	29
50	2/3/2015	6.6	13.1	0.5	947	1761	8	32
50	2/16/2015	6.5	19.2	0.7	256	1158	10	26
50	2/23/2015	6.8	17.6	0.6	385	1230	5	30
50	3/3/2015	6.7	18.8	0.7	304	1174	10	28
50	3/16/2015	6.6	23.1	1.0	114	1228	18	48
50	4/14/2015	6.7	19.9	1.1	95	1321	17	47
50	5/18/2015	6.9	13.6	0.7	241	1267	10	73
50	5/25/2015	6.9	18.2	1.0	10	1248	19	53
50	6/9/2015	7.0	23.0	1.4	5	1388	28	66
50	9/9/2015	6.5		0.6	355	2078	13	59
50	9/15/2015	6.7	27.5	1.3	40	2469	32	120
50	10/19/2015	6.5	43.6	1.9	25	2276	70	127
50	11/9/2015	6.8	29.7	1.0	69	2118	16	96
50	12/14/2015	7.4	42.1	1.3	130	709	7	19
50	1/18/2016	6.5	52.0	2.1	17	2055	55	152
50	1/28/2016	6.9	35.8	0.9	286	1291	18	61
50	2/15/2016	6.5	23.0	1.0	260	1285	12	34
50	3/14/2016	6.7	19.6	0.9	101	938	10	33
50	4/11/2016	6.8	23.8	1.0	45	1063	10	37
50	5/16/2016	7.1	35.0	1.7	89	2282	33	75
50	6/20/2016	7.3	39.6	2.3	22	6681	76	151
50	11/7/2016	6.4	50.2	0.4	1103	2275	5	38
50	11/15/2016	6.6	25.3	0.4	1631	3033	9	38
50	11/22/2016	6.4	19.1	0.4	1199	2464	3	27
50	12/12/2016	6.7	31.4	0.7	120	1080	3	27
50	1/10/2017	6.3	146.1	1.0	54	1422	5	75
50	1/30/2017	6.6	26.9	0.8	63	989	10	37
50	2/13/2017	6.6	58.1	1.9	95	1179	5	37
50	3/14/2017	6.8	22.2	0.7	131	975	5	27
50	3/20/2017	6.6	24.2	1.0	156	1106	7	31

50	4/10/2017	6.9	25.8	1.1	130	1407	10	36
50	5/15/2017	7.0	44.1	1.7	244	2661	43	88
50	6/12/2017	6.9	112.6	1.9	66	2399	66	144
50	7/18/2017	7.5	115.0	8.2	2	50695	3746	3748
50	9/12/2017	7.2	63.3	4.1	1	21755	2326	2429
50	10/16/2017	6.7	21.1	0.9	43	2118	146	208
50	11/15/2017	6.8	21.4	1.1	50	1644	57	98
50	12/11/2017	7.1	17.3	0.9	116	1062	23	45
50	1/15/2018	7.0	36.5	2.1	27	10231	745	905
50	2/12/2018	6.8	24.4	1.2	47	1660	95	136
50	3/19/2018	6.8	27.8	1.2	73	1259	37	75
50	3/27/2018	6.8	21.6	0.9	101	918	25	51
50	4/16/2018	7.1	16.0	0.7	42	879	27	56
50	5/14/2018	7.2	25.0	1.5	24	1669	112	159
50	12/10/2018	6.5	26.4	0.3	2042	2666	14	44
50	1/14/2019	6.5	34.9	0.6	107	1129	10	28
50	2/11/2019	6.5	21.0	0.4	555	1383	9	41
50	3/5/2019	6.5	26.8	0.6	154	997	18	40
50	3/18/2019	6.6	18.0	0.5	242	995	6	27
50	4/24/2019	6.4	27.5	0.5	51	1277	11	48
50	5/21/2019	6.5	36.4	0.6	5	2384	42	139
50	10/14/2019	6.8	39.9	0.8	210	1892	13	156
50	11/11/2019	6.8	27.4	0.8	74	932	25	58
50	12/9/2019	6.9	18.3	0.7	142	1523	46	83
50	1/13/2020	7.0	20.3	0.9	196	1217	26	66
50	2/11/2020	6.9	24.0	0.8	182	1363	28	83
50	3/9/2020	7.1	37.8	0.7	247	1216	12	39
50	4/6/2020	6.9	64.8	1.0	134	1315	20	53
50	5/4/2020	7.1	26.1	1.4	229	3941	208	325
50	5/18/2020	7.2	39.1	1.4	11	1467	37	83
50	6/8/2020	6.7	79.0	1.9	13	2964	140	238
50	7/6/2020	6.8	76.5	1.3	50	1858	16	152
50	12/9/2020	6.2		0.4	2685	3501	23	55
50	1/11/2021		31.3		1068	2048	8	27
50	1/26/2021		22.5		525	1512	6	26
50	2/8/2021		45.4		243	1316	11	32
50	2/22/2021		48.1		246	1140	13	42
50	3/9/2021		33.5		89	1045	8	37
50	3/29/2021		30.2		94	1121	12	46
50	4/19/2021		49.8		44	1558	15	68
50	5/17/2021		36.7		46	1983	35	120
50	6/7/2021		32.1		19	2004	81	152
50	10/11/2021		64.1		3	1669	46	105
50	11/10/2021		37.1		1065	2375	13	60
50	11/22/2021		65.0		1904	3652	5	35
50	12/6/2021		104.6		455	1825	6	33
50	12/14/2021		36.5		745	2100	8	42
50	1/17/2022		36.0		556	1609	8	29
50	1/31/2022		18.5		933	2009	7	35
50	2/14/2022		23.7		417	1495	5	28

50	2/28/2022		17.7		429	1289	6	22
50	3/15/2022		27.8		169	1198	5	28
50	4/19/2022		23.3		66	1150	7	32
50	5/16/2022		37.4		22	2623		
50	6/7/2022		33.3		24	2472		
50	11/8/2022		17.3					
50	11/29/2022		35.9		4211	5668	9	36
50	12/13/2022		58.6		3652	5300	7	28
57	3/27/2012	7.2	23.3	1.0	1995	2646	35	66
57	4/2/2012	7.0	21.2	0.8	1422	2231	25	58
57	4/10/2012	7.1	22.2	1.0	1224	1975	25	51
57	5/15/2012	7.4		1.4	1473	2415	31	54
57	5/15/2012	7.1		1.2	1561	2526	20	42
57	6/19/2012	6.6		0.4	379	1635	56	146
57	7/10/2012	7.2	25.9	1.5	575	1995	74	131
57	7/30/2012	7.1	29.2	1.1	371	1997	86	175
57	7/31/2012	7.0	18.2	0.8	150	1833	65	133
57	8/2/2012	7.3	23.1	1.2	153	1685	70	125
57	8/20/2012	7.3		3.0	7	3752	140	830
57	9/10/2012	7.0		1.1	471	2863	63	290
57	10/16/2012	6.5		0.3	755	2337	83	229
57	10/18/2012	6.5	10.3	0.4	477	1797	39	108
57	10/23/2012	6.5	11.2	0.4	422	1675	49	108
57	11/12/2012	6.9		0.7	682	990	4	24
57	12/12/2012	6.7	15.9	0.8	574	1787	44	97
57	1/16/2013	6.8	16.4	0.8	493	1442	33	68
57	2/11/2013	6.9	19.0	0.9	346	1432	47	81
57	3/18/2013	7.0		2.0	551	1932	167	184
57	4/10/2013	7.1	29.3	1.3	677	1744	61	130
57	4/16/2013	6.3		0.2	682	1438	40	136
57	4/18/2013	6.4	7.7	0.2	696	1574	38	138
57	4/22/2013	6.6	10.4	0.4	452	1329	31	87
57	4/29/2013	6.9	15.6	0.8	363	1411	48	97
57	5/15/2013	7.4	32.6	1.4	286	2034	107	179
57	6/10/2013	7.4		2.2	214	2975	130	217
57	7/2/2013	7.3	53.9	2.6	137	2333	167	213
57	7/16/2013	7.4	54.3	2.5	131	3045	227	294
57	8/6/2013	6.8		2.0	0	2459	2212	2221
57	9/18/2013	7.2	59.4	1.9	170	2061	289	374
57	10/14/2013	7.1	57.7	1.9	58	2301	608	698
57	10/29/2013	6.8	51.8	0.9	3785	5262	68	183
57	11/4/2013	7.0	47.3	1.1	2471	4013	66	150
57	11/12/2013	6.9	26.3	0.7	2021	2962	50	102
57	12/11/2013	6.9	19.3	0.5	1255	1975	29	75
57	1/14/2014	6.7	16.2	0.5	824	1884	47	83
57	2/11/2014	6.7	14.0	0.4	1227	1945	33	88
57	3/3/2014	7.0	20.6	0.6	747	1617	34	69
57	3/10/2014	7.2	21.3	0.8	633	1479	20	59
57	4/1/2014	7.2	20.4	0.8	854	1554	7	61
57	4/14/2014	7.2	17.1	0.7	639	1474	92	133

57	5/13/2014	7.4	26.0	1.0	631	2010	66	152
57	5/19/2014				378	1914	58	168
57	6/10/2014	7.4	53.9	2.9	16	4443	509	825
57	7/14/2014	7.7	54.8	3.3	4	2385	76	914
57	8/24/2014	7.6	85.7	5.4	7	9890	124	287
57	9/15/2014	7.2	110.2	5.3	1	7400	121	175
57	10/13/2014	6.5	20.1	0.4	1595	2554	23	76
57	11/10/2014	6.8	20.0	0.6	539	2089	19	62
57	12/8/2014	7.1	26.0	0.9	1034	2044	16	65
57	1/19/2015	6.7	15.0	0.4	1064	1763	12	42
57	2/3/2015	6.7	13.1	0.4	1113	1836	18	61
57	2/16/2015	6.9	19.6	0.7	759	1600	12	39
57	2/23/2015	6.8	18.1	0.6	1408	2115	16	54
57	3/3/2015	6.9	19.3	0.7	1000	2014	16	53
57	3/16/2015	7.1	23.6	1.1	912	1810	14	44
57	4/14/2015	7.2	23.4	1.1	653	1613	28	59
57	5/18/2015	6.8	18.7	0.6	2537	3762	23	121
57	5/25/2015	7.1	17.2	0.8	623	1687	23	71
57	6/9/2015	7.1	19.5	1.0	486	1662	43	111
57	7/14/2015	7.2	44.8	2.4	73	2505	231	354
57	8/18/2015	7.5	57.1	3.2	6	2943	281	356
57	9/9/2015	6.6		0.4	114	1490	21	122
57	9/15/2015	6.9	24.5	1.0	101	1620	29	138
57	10/19/2015	7.2	55.0	2.8	229	2362	40	103
57	11/9/2015	7.0	39.9	1.2	860	3078	31	115
57	12/14/2015	7.0	22.9	1.0	613	1659	14	51
57	1/18/2016	6.8	36.2	1.6	755	1765	25	55
57	1/28/2016	6.9	25.0	0.7	1490	2664	30	105
57	2/15/2016	6.9	22.5	0.8	733	1785	46	121
57	3/14/2016	7.0	25.2	0.9	849	1611	21	55
57	4/11/2016	7.1	28.7	1.3	609	1629	33	64
57	5/16/2016	7.4	44.9	1.9	1219	3018	42	97
57	6/20/2016	7.3	27.2	1.1	423	2001	58	141
57	9/13/2016	7.4	62.9	3.2	2916	3423	174	203
57	10/10/2016	7.5	63.1	3.0	2400	4781	482	495
57	11/7/2016	6.9	34.6	0.8	2841	4334	11	71
57	11/15/2016	6.6	25.4	0.3	3814	4675	33	120
57	11/22/2016	6.5	18.9	0.3	1940	3250	20	63
57	12/12/2016	6.8	33.4	0.9	1829	2585	25	65
57	1/10/2017	6.9	36.7	1.0	1788	2553	14	47
57	1/30/2017	6.9	23.4	0.8	1124	2117	8	47
57	2/13/2017	7.1	36.0	1.4	1593	2582	25	96
57	3/14/2017	6.9	22.5	0.6	1692	2409	14	52
57	3/20/2017	7.1	22.7	0.8	1306	2199	18	58
57	4/10/2017	7.3	30.3	1.2	1020	1894	35	77
57	5/15/2017	7.6	50.6	2.4	1000	1939	49	117
57	6/12/2017	7.4	60.9	3.0	804	4218	163	209
57	7/18/2017	7.7	77.4	4.0	1935	9480	91	165
57	8/14/2017	7.5	69.2	2.8	3895	9936	172	221
57	9/12/2017	6.9	47.5	1.6	102	2627	142	204

57	10/16/2017	6.8	18.5	0.5	1232	2592	44	85
57	11/15/2017	6.9	22.6	0.7	988	2152	35	88
57	12/11/2017	6.8	20.2	0.6	1172	2277	30	73
57	1/15/2018	7.4	23.4	0.8	1191	1812	25	103
57	2/12/2018	7.3	23.5	0.8	816	1921	25	44
57	3/19/2018	7.1	22.5	0.7	874	1674	22	46
57	3/27/2018	6.9	21.6	0.6	2126	2758	22	55
57	4/16/2018	7.1	19.9	0.8	747	1478	23	52
57	5/14/2018	7.4	38.0	1.8	589	1628	76	107
57	8/13/2018	7.1	83.8	0.7	3696	5266	59	149
57	9/10/2018	7.5	79.3	3.5	488	8478	167	235
57	10/15/2018	7.0	83.1	2.2	0	2123	1174	1380
57	11/5/2018	7.3	52.7	0.9	2830	4269	40	134
57	12/10/2018	6.9	23.9	0.2	3643	4593	23	58
57	1/14/2019	7.1	29.0	0.6	2484	3003	12	33
57	2/11/2019	6.3	17.2	0.2	2575	4148	20	64
57	3/5/2019	6.8	29.4	0.5	1568	2238	20	55
57	3/18/2019	6.5	16.5	0.3	2202	2836	24	70
57	4/24/2019	7.4	52.9	1.8	1486	2365	32	69
57	5/21/2019	7.4	60.3	2.4	710	2611	100	156
57	5/27/2019	7.4	75.5	3.4	487	3353	77	145
57	7/8/2019	7.3	70.9	2.2	1237	3506	141	186
57	8/12/2019	6.9	58.1	0.7	2673	3947	64	169
57	9/17/2019	7.4	58.4	2.0	0	1092	395	438
57	10/14/2019	6.8	38.3	1.2	705	5186	80	342
57	11/11/2019	7.0	27.6	0.7	1805	2826	30	70
57	12/9/2019	6.9	19.9	0.5	2080	3185	26	77
57	1/13/2020	7.1	29.1	1.0	1358	2125	22	51
57	2/11/2020	7.0	26.2	0.8	2754	3921	45	134
57	3/9/2020	6.9	20.2	0.6	2208	2853	24	66
57	4/6/2020	7.2	32.6	1.4	1137	2109	53	77
57	5/4/2020	7.1	21.6	0.7	1995	2760	27	90
57	5/18/2020	7.4	33.1	1.5	973	1727	28	79
57	6/8/2020	7.5	47.4	2.3	923	1983	85	133
57	7/6/2020	7.3	50.2	1.9	7	1818	194	392
57	9/15/2020	7.1		2.5	0	849	559	728
57	12/9/2020	6.9		0.7	2708	3628	45	91
57	1/11/2021		23.6		1302	2118	27	58
57	1/26/2021		22.3		1636	2498	21	48
57	2/8/2021		30.4		1485	2304	21	43
57	2/22/2021		38.5		1862	2694	38	80
57	3/9/2021		28.8		1476	1984	13	41
57	3/29/2021		30.7		1300	1734	24	68
57	4/19/2021		49.6		709	1563	65	115
57	5/17/2021		34.8		517	1520	37	114
57	6/7/2021		28.8		943	1777	52	93
57	10/11/2021		41.5		660	1633	41	90
57	11/10/2021		28.5		1888	2871	28	87
57	11/22/2021		31.4		1044	2168	29	81
57	12/6/2021		50.0		1023	2225	36	68

57	12/14/2021		19.3		735	1756	19	71
57	1/17/2022		22.3		1189	2387	16	153
57	1/31/2022		19.8		2474	3761	20	65
57	2/14/2022		24.5		1018	2043	39	100
57	2/28/2022		20.5		2206	3024	16	42
57	3/15/2022		30.7		1763	2551	21	45
57	4/19/2022		27.9		1594	2546	14	58
57	5/16/2022		55.1		1022	2037		
57	6/7/2022		35.0		2467	3719		
57	10/10/2022		94.6		3	1134	823	974
57	11/8/2022		96.0		494	1310	45	109
57	11/29/2022		30.8		3967	5355	17	70
57	12/13/2022		31.5		1611	2614	10	50
58	3/27/2012	7.0	25.2	0.8	100	768	13	30
58	4/2/2012	6.9	23.6	0.7	150	786	11	26
58	4/10/2012	7.0	21.2	0.8	92	750	14	32
58	5/15/2012	7.0		1.1	24	796	24	40
58	6/19/2012	6.8		0.5	53	1071	22	74
58	7/10/2012	7.2	19.5	1.4	13	1029	39	72
58	7/30/2012	6.9	16.8	0.8	0	1520	48	107
58	7/31/2012	6.9	16.8	1.1	0	1252	37	75
58	8/2/2012	7.1	18.9	1.3	0	960	36	61
58	8/20/2012	7.0		2.0	13	907	40	85
58	9/10/2012	6.9		0.7	16	1326	54	144
58	10/16/2012	6.5		0.4	230	1385	25	95
58	10/18/2012	6.6	9.3	0.5	119	1190	23	66
58	10/23/2012	6.6	9.8	0.5	86	1096	22	54
58	11/12/2012	7.0		0.7	79	825	8	31
58	12/12/2012	6.8	12.9	0.8	85	863	22	49
58	1/16/2013	6.9	13.5	0.7	82	776	24	42
58	2/11/2013	6.9	19.9	0.9	64	811	22	39
58	3/18/2013	7.0		1.3	104	782	55	93
58	4/10/2013	6.9	32.3	1.0	236	1007	27	54
58	4/16/2013	6.4		0.2	333	1010	13	52
58	4/18/2013	6.4	9.5	0.2	249	934	11	46
58	4/22/2013	6.6	13.0	0.4	198	858	10	36
58	4/29/2013	6.9	18.0	0.8	151	899	17	37
58	5/15/2013	7.2	28.5	1.1	81	966	39	64
58	6/10/2013	7.1		1.5	7	654	34	58
58	7/2/2013	7.0	15.1	1.0	8	793	28	66
58	7/16/2013	7.1	16.4	1.2	8	747	31	61
58	8/6/2013	7.3		2.0	0	983	9	65
58	9/18/2013	6.8	74.2	1.2	2	748	51	111
58	10/14/2013	7.2	73.8	1.8	105	593	36	55
58	10/29/2013	7.0	34.6	0.7	665	1589	32	93
58	11/4/2013	7.0	31.9	0.7	447	1687	10	192
58	11/12/2013	6.7	17.3	0.6	222	1026	16	43
58	12/11/2013	6.8	17.4	0.4	140	825	16	38
58	1/14/2014	6.6	11.6	0.4	136	815	7	31
58	2/11/2014	6.6	13.8	0.4	175	770	13	27

58	3/3/2014	6.8	17.9	0.5	137	825	11	36
58	3/10/2014	7.0	17.1	0.7	88	705	11	32
58	4/1/2014	6.9	15.6	0.6	84	683	17	29
58	4/14/2014	7.0	14.1	0.5	62	728	11	31
58	5/13/2014	7.2	20.1	0.8	34	853	21	66
58	6/10/2014	7.6	38.9	2.3	0	710	23	106
58	10/13/2014	6.6	12.9	0.4	183	1242	12	44
58	11/10/2014	6.9	13.6	0.5	85	1091	14	43
58	12/8/2014	7.1	16.6	0.7	137	1081	15	53
58	1/19/2015	6.7	14.2	0.4	183	859	9	30
58	2/3/2015	6.5	11.8	0.3	187	855	9	32
58	2/16/2015	6.7	15.6	0.5	104	756	11	26
58	2/23/2015	6.7	17.5	0.4	204	877	8	27
58	3/3/2015	6.8	16.4	0.5	132	830	11	30
58	3/16/2015	6.8	17.9	0.7	92	801	17	35
58	4/14/2015	6.9	16.8	0.7	68	733	18	38
58	5/18/2015	6.7	12.5	0.5	213	1068	11	75
58	5/25/2015	6.9	12.8	0.6	10	969	14	40
58	6/9/2015	7.1	13.9	0.8	21	946	22	52
58	9/9/2015	6.5		0.4	40	1214	15	48
58	9/15/2015	6.8	13.6	0.7	77	1216	29	84
58	11/9/2015	7.0	19.0	0.9	153	1659	33	102
58	12/14/2015	7.1	14.2	0.7	106	1036	15	40
58	1/18/2016	7.0	23.5	1.1	145	1004	32	92
58	1/28/2016	6.8	23.6	0.5	363	1278	19	91
58	2/15/2016	6.8	18.8	0.6	152	813	10	30
58	3/14/2016	7.0	22.0	0.7	128	742	11	27
58	4/11/2016	7.0	22.3	0.9	123	833	20	41
58	5/16/2016	7.2	33.6	1.2	71	1110	44	77
58	6/20/2016	7.1	25.5	1.4	262	1147	27	67
58	11/7/2016	6.7	20.8	0.4	732	1690	16	52
58	11/15/2016	6.5	19.4	0.2	494	1339	14	44
58	11/22/2016	6.3	12.9	0.3	157	1012	6	30
58	12/12/2016	6.9	22.9	0.6	117	777	9	31
58	1/10/2017	6.8	32.1	0.7	131	769	9	33
58	1/30/2017	6.9	19.8	0.6	108	742	7	27
58	2/13/2017	6.9	24.6	1.0	104	744	12	37
58	3/14/2017	6.9	20.7	0.5	143	793	5	24
58	3/20/2017	7.0	20.0	0.6	112	738	7	22
58	4/10/2017	7.1	27.0	0.9	51	719	18	33
58	5/15/2017	7.3	42.2	1.6	18	740	44	93
58	6/12/2017	7.3	52.9	1.4	55	967	47	96
58	9/12/2017	7.3	36.8	1.0	11	795	28	43
58	10/16/2017	6.7	13.5	0.4	60	1394	11	42
58	11/15/2017	6.8	14.8	0.6	54	1257	13	47
58	12/11/2017	7.0	15.0	0.5	76	1034	13	35
58	1/15/2018	7.1	16.4	0.7	58	851	18	39
58	2/12/2018	7.0	33.3	0.6	137	1018	20	42
58	3/19/2018	6.9	26.0	0.6	97	767	12	32
58	3/27/2018	6.9	20.0	0.5	152	719	10	31

58	4/16/2018	7.1	22.6	0.6	81	799	16	46
58	5/14/2018	7.3	24.5	1.0	29	797	31	56
58	12/10/2018	6.7	16.2	0.3	263	1207	7	35
58	1/14/2019	6.8	24.4	0.5	133	865	9	26
58	2/11/2019	6.4	20.5	0.2	344	1081	10	35
58	3/5/2019	6.7	25.0	0.4	144	831	8	26
58	3/18/2019	6.6	15.7	0.3	187	808	5	25
58	4/24/2019	7.2	39.4	1.2	10	639	14	39
58	5/21/2019	7.2	53.3	1.6	1	630	13	31
58	8/12/2019	7.0	87.1	1.2	6	1025	64	113
58	9/17/2019	7.2	56.3	1.8	13	748	17	50
58	10/14/2019	7.1	32.9	1.0	64	1035	36	93
58	11/11/2019	6.9	18.9	0.7	107	877	17	54
58	12/9/2019	6.9	13.7	0.5	116	1118	11	45
58	1/13/2020	7.1	18.4	0.9	61	880	20	50
58	2/11/2020	7.0	18.3	0.6	250	1270	15	62
58	3/9/2020	7.1	12.2	0.5	108	912	11	37
58	4/6/2020	7.2	21.2	1.0	53	919	23	49
58	5/4/2020	7.0	14.9	0.6	152	990	20	68
58	5/18/2020	7.3	20.8	1.1	14	966	25	57
58	6/8/2020	7.4	34.8	1.8	53	954	41	84
58	7/6/2020	7.8	45.0	2.3	63	827	21	74
58	12/9/2020	6.9		0.7	2002	2946	18	50
58	1/11/2021		16.3		183	1055	11	35
58	1/26/2021		13.5		148	1016	10	31
58	2/8/2021		17.9		144	1036	14	33
58	2/22/2021		33.1		329	1008	14	43
58	3/9/2021		18.1		126	906	9	43
58	3/29/2021		21.9		88	773	9	30
58	4/19/2021		31.3		45	693	16	37
58	5/17/2021		20.7		127	1025	14	66
58	6/7/2021		15.4		44	882	17	50
58	10/11/2021		23.9		31	1147	28	63
58	11/10/2021		15.7		227	1330	17	57
58	11/22/2021		17.9		99	1331	17	48
58	12/14/2021		16.9		195	1153	10	41
58	1/17/2022		17.9		170	1029	9	27
58	1/31/2022		14.8		449	1432	10	36
58	2/14/2022		20.2		137	934	10	31
58	2/28/2022		13.8		155	887	7	25
58	3/15/2022		19.6		121	848	8	27
58	4/19/2022		22.0		128	888	8	27
58	5/16/2022		38.4		23	747		
58	6/7/2022		22.5		141	1113		
58	10/10/2022		46.5		73	809	50	78
58	11/8/2022		36.0		153	760	33	63
58	11/29/2022		18.3		585	1760	24	56
58	12/13/2022		15.5		151	1269	12	35
59	4/18/2013	6.0	3.5	0.1	82	827	6	42
59	4/22/2013	6.3	4.7	0.2	44	961	4	36

59	4/29/2013	6.5	6.0	0.3	18	1298	6	31
59	5/15/2013	6.8	8.8	0.4	24	1893	13	54
59	6/10/2013	7.1		0.9	66	1979	14	58
59	10/29/2013	6.7	63.4	0.5	1804	2574	5	32
59	11/4/2013	6.6	53.7	0.6	1149	1763	4	19
59	11/12/2013	6.5	19.5	0.2	199	842	3	21
59	12/11/2013	6.6	11.5	0.2	115	837	3	20
59	1/14/2014	6.2	7.2	0.1	19	796	0	16
59	2/11/2014	6.4	6.5	0.2	65	760	6	18
59	3/3/2014	6.6	8.6	0.2	130	907	1	15
59	3/10/2014	6.7	8.2	0.3	49	768	5	16
59	4/1/2014	6.6	7.1	0.2	33	845	4	30
59	4/14/2014	6.7	7.2	0.2	29	862	2	15
59	5/13/2014	6.9	11.2	0.4	97	1044	4	28
59	10/13/2014	6.7	13.9	0.2	125	947	4	25
59	11/10/2014	6.7	11.8	0.2	7	923	8	20
59	12/8/2014	7.0	14.0	0.4	193	1070	2	22
59	1/19/2015	6.5	6.7	0.2	99	884	3	16
59	2/3/2015	6.2	5.1	0.1	56	826	3	16
59	2/16/2015	6.6	7.6	0.2	54	834	3	12
59	2/23/2015	6.6	7.8	0.2	130	897	1	12
59	3/3/2015	6.7	8.7	0.3	106	931	3	17
59	3/16/2015	6.9	9.2	0.4	64	914	4	17
59	4/14/2015	6.6	7.8	0.3	29	1001	3	17
59	5/18/2015	6.9	9.4	0.3	175	1117	5	56
59	5/25/2015	6.6	6.6	0.3	2	1253	7	44
59	6/9/2015	6.7	6.8	0.2	11	1335	5	26
59	9/9/2015	6.6		0.3	19	1089	4	34
59	9/15/2015	6.6	12.3	0.3	18	1146	14	34
59	11/9/2015	7.0	19.1	0.5	395	1685	7	47
59	12/14/2015	6.9	9.2	0.3	37	1143	3	20
59	1/28/2016	6.8	9.7	0.4	387	1356	14	60
59	2/15/2016	6.7	8.1	0.3	48	959	2	18
59	3/14/2016	6.9	9.3	0.4	87	897	3	17
59	4/11/2016	6.9	10.7	0.5	53	1051	2	21
59	5/16/2016	7.1	12.5	0.7	18	1432	11	44
59	6/20/2016	6.9	22.2	0.4	498	1483	3	38
59	11/7/2016	6.3	45.9	0.3	1084	1887	1	16
59	11/15/2016	6.6	16.7	0.2	403	1169	3	29
59	11/22/2016	6.1	11.1	0.1	65	988	0	21
59	12/12/2016	6.9	14.3	0.2	199	872	1	14
59	1/10/2017	6.8	13.5	0.2	212	915	0	16
59	1/30/2017	6.9	12.0	0.3	98	779	1	13
59	2/13/2017	6.9	15.0	0.6	121	819	1	19
59	3/14/2017	6.9	11.1	0.2	133	799	3	30
59	3/20/2017	7.0	10.4	0.3	72	837	2	17
59	4/10/2017	7.1	12.8	0.5	43	853	3	17
59	5/15/2017	7.1	16.0	0.7	14	777	7	16
59	10/16/2017	6.6	11.6	0.1	14	1322	1	27
59	11/15/2017	6.8	10.9	0.3	60	1242	1	23

59	12/11/2017	6.9	8.0	0.2	60	1167	1	16
59	1/15/2018	7.1	8.9	0.3	39	926	2	15
59	2/12/2018	7.1	7.6	0.2	102	1010	2	14
59	3/19/2018	6.9	7.5	0.3	47	890	2	23
59	3/27/2018	6.9	7.3	0.2	87	841	1	20
59	4/16/2018	7.1	7.9	0.3	45	877	4	18
59	5/14/2018	7.2	10.2	0.4	15	1218	15	29
59	12/10/2018	6.6	19.8	0.2	211	951	2	20
59	1/14/2019	6.9	17.5	0.2	88	782	1	12
59	2/11/2019	6.2	9.1	0.1	160	972	2	24
59	3/5/2019	6.7	11.5	0.2	162	845	2	16
59	3/18/2019	6.4	7.3	0.1	75	750	0	15
59	4/24/2019	6.9	16.4	0.5	20	742	2	17
59	5/21/2019	6.9	23.5	1.0	14	737	1	21
59	8/12/2019	6.7	63.1	0.5	205	1054	3	34
59	9/17/2019	6.3	65.6	0.3	3	492	2	10
59	10/14/2019	6.9	47.7	0.6	1174	2336	8	60
59	11/11/2019	6.9	19.0	0.4	147	758	5	25
59	12/9/2019	6.7	9.1	0.2	77	1136	4	24
59	1/13/2020	7.0	10.3	0.3	28	893	4	24
59	2/11/2020	7.0	11.5	0.3	282	1358	6	36
59	3/9/2020	6.9	6.8	0.2	25	1089	3	21
59	4/6/2020	7.0	9.9	0.4	20	1200	4	23
59	5/4/2020	6.9	10.8	0.4	79	1134	6	36
59	5/18/2020	7.0	11.7	0.5	12	1317	4	31
59	6/8/2020	7.1	18.4	0.9	27	1542	5	41
59	12/9/2020	6.5		0.4	772	1210	1	10
59	1/11/2021		19.4		89	788	2	14
59	1/26/2021		13.6		76	854	1	12
59	2/8/2021		17.8		52	792	3	15
59	2/22/2021		19.7		504	1161	5	22
59	3/9/2021		14.9		37	740	1	12
59	3/29/2021		15.4		28	699	2	15
59	4/19/2021		19.6		17	648	2	20
59	5/17/2021		15.5		30	1078	6	41
59	6/7/2021		12.0		18	1134	3	29
59	10/11/2021		52.7		44	580	4	23
59	11/10/2021		29.2		1012	1678	8	35
59	11/22/2021		36.3		199	831	1	15
59	12/6/2021		48.4		89	686	3	12
59	12/14/2021		25.7		454	1235	2	26
59	1/17/2022		17.8		184	939	2	16
59	1/31/2022		12.4		283	1103	3	24
59	2/14/2022		13.3		99	972	1	20
59	2/28/2022		9.0		82	959	1	15
59	3/15/2022		12.9		70	1013	2	16
59	4/19/2022		13.3		98	1049	4	23
59	5/16/2022		22.0		27	940		
59	6/7/2022		16.5		40	1439		
59	11/8/2022		55.9		400	958	8	23

59	11/29/2022		15.4		1457	2330	6	30
59	12/13/2022		30.2		217	928	3	13
60	4/16/2013	6.6		1.2	361	1000	27	91
60	4/18/2013	6.6	4.9	0.2	283	890	28	102
60	4/22/2013	6.6	5.2	0.3	305	873	32	203
60	4/29/2013	7.3	18.2	1.5	178	776	42	104
60	5/15/2013	7.6	33.8	3.1	34	447	47	87
60	6/10/2013	7.8		3.7	17	511	45	71
60	10/29/2013	6.7	21.4	0.5	229	1817	155	487
60	11/4/2013	6.8	17.5	0.7	153	1429	98	305
60	11/12/2013	6.8	15.3	0.6	164	820	36	97
60	12/11/2013	7.0	12.2	0.5	245	1009	50	150
60	1/14/2014	7.0	13.1	0.7	152	686	31	63
60	2/11/2014	6.9	10.1	0.6	236	840	31	98
60	3/3/2014	7.0	13.3	0.8	279	1175	61	194
60	3/10/2014	7.3	18.8	1.2	119	549	17	50
60	4/1/2014	7.2	19.3	1.4	97	496	15	40
60	4/14/2014	7.3	16.2	1.0	101	621	19	52
60	5/13/2014	7.3	19.7	1.3	166	970	29	127
60	10/13/2014	6.7	10.4	0.4	210	1438	68	216
60	11/10/2014	7.1	15.3	0.8	180	991	46	135
60	12/8/2014	7.3	15.9	1.0	377	1603	54	276
60	1/19/2015	7.1	12.2	0.6	213	885	27	111
60	2/3/2015	6.7	7.5	0.4	243	1306	47	219
60	2/16/2015	7.1	16.3	1.0	156	678	24	66
60	2/23/2015	6.9	10.9	0.6	170	877	20	94
60	3/3/2015	7.1	14.2	0.8	182	999	43	158
60	3/16/2015	7.3	21.2	1.6	98	523	17	41
60	4/14/2015	7.3	24.0	1.8	16	446	20	39
60	5/18/2015	6.7	17.1	0.4	6839	8570	44	146
60	5/25/2015	7.4	22.1	1.8	41	923	28	59
60	6/9/2015	7.5	27.3	2.3	95	610	26	47
60	9/9/2015	7.2		1.6	78	839	32	88
60	9/15/2015	7.4	35.7	2.7	171	742	28	59
60	11/9/2015	7.4	26.2	1.6	583	2946	85	354
60	12/14/2015	7.3	23.2	1.7	158	1055	33	112
60	1/28/2016	6.7	11.2	0.4	1337	2756	62	216
60	2/15/2016	7.1	19.5	1.3	216	848	29	72
60	3/14/2016	7.2	16.8	1.2	611	1641	61	211
60	4/11/2016	7.3	28.0	2.1	107	685	53	91
60	5/16/2016	7.7	38.1	3.0	247	790	52	70
60	6/20/2016	7.5	32.2	1.9	1332	2026	42	108
60	11/7/2016	7.1	24.1	0.8	190	919	29	88
60	11/15/2016	6.6	9.8	0.2	833	1893	58	150
60	11/22/2016	6.7	13.5	0.4	644	1835	43	197
60	12/12/2016	7.0	20.0	1.1	444	1122	30	126
60	1/10/2017	7.1	21.6	1.2	521	1145	30	78
60	1/30/2017	7.2	19.0	1.1	291	758	15	61
60	2/13/2017	7.2	30.0	2.0	241	496	9	31
60	3/14/2017	7.1	14.8	0.7	345	950	20	71

60	3/20/2017	7.3	19.6	1.1	294	825	16	64
60	4/10/2017	7.5	26.5	1.8	95	544	15	48
60	5/15/2017	7.7	37.4	2.9	6	645	25	48
60	6/12/2017	7.7	45.8	4.2	102	1643	40	254
60	10/16/2017	6.8	13.8	0.7	89	1060	40	116
60	11/15/2017	6.9	15.9	0.9	122	821	21	71
60	12/11/2017	7.0	14.7	0.8	166	908	27	87
60	1/15/2018	7.1	20.7	1.3	115	544	15	41
60	2/12/2018	7.1	20.1	1.3	272	804	20	53
60	3/19/2018	7.1	20.7	1.3	278	789	22	63
60	3/27/2018	6.9	13.7	0.8	282	766	25	75
60	4/16/2018	7.1	16.1	1.0	426	1192	42	156
60	5/14/2018	7.5	32.2	2.4	1	476	27	50
60	12/10/2018	6.8	13.3	0.5	169	1169	43	171
60	1/14/2019	7.1	21.1	1.0	190	632	17	50
60	2/11/2019	6.6	8.5	0.3	233	922	25	72
60	3/5/2019	7.1	14.8	0.7	161	800	24	75
60	3/18/2019	6.7	9.6	0.3	171	858	30	107
60	4/24/2019	7.7	36.6	2.3	7	313	9	21
60	5/21/2019	7.7	42.1	2.9	5	515	14	51
60	8/12/2019	7.4	28.5	1.3	41	1488	49	199
60	9/17/2019	7.4	42.2	3.0	7	673	18	61
60	10/14/2019	6.8	16.6	0.7	389	2920	122	574
60	11/11/2019	7.1	17.8	1.0	152	868	50	162
60	12/9/2019	7.0	11.8	0.6	138	1078	45	146
60	1/13/2020	7.3	20.8	2.0	162	586	25	67
60	2/11/2020	7.0	13.5	0.7	479	1737	61	215
60	3/9/2020	7.3	17.6	1.0	122	794	38	105
60	4/6/2020	7.4	34.5	2.6	104	518	24	42
60	5/4/2020	7.3	15.6	1.0	135	1227	60	199
60	5/18/2020	8.0	34.3	2.5	0	642	32	90
60	6/8/2020	7.8	47.0	4.0	1	727	18	93
60	12/9/2020	7.2		1.5	338	901	25	70
60	1/11/2021		18.7		234	734	22	57
60	1/26/2021		16.3		187	741	19	55
60	2/8/2021		27.4		216	583	20	39
60	2/22/2021		17.1		682	1400	48	119
60	3/9/2021		26.7		159	526	10	33
60	3/29/2021		28.0		137	485	20	50
60	4/19/2021		39.8		20	327	19	23
60	5/17/2021		18.9		417	2331	85	388
60	6/7/2021		35.3		68	519	22	44
60	10/11/2021		33.4		20	638	22	49
60	11/10/2021		17.3		376	1363	59	141
60	11/22/2021		33.8		185	662	21	44
60	12/6/2021		48.7		229	559	23	47
60	12/14/2021		19.3		453	1223	36	88
60	1/17/2022		22.7		310	820	28	57
60	1/31/2022		14.2		659	1497	35	100
60	2/14/2022		20.7		225	789	31	80

60	2/28/2022		16.2		208	697	18	49
60	3/15/2022		29.7		155	480	15	30
60	4/19/2022		25.3		378	1033	20	79
60	5/16/2022		44.9		5	464		
60	6/7/2022		34.2		99	754		
60	11/8/2022		29.6		1002	2919	145	417
60	11/29/2022		12.5		755	1742	46	118
60	12/13/2022		26.5		428	858	19	37
5702	10/13/2014	6.3	26.7	0.8	452	32051	2848	8252
5702	11/10/2014	6.6	33.4	1.1	0	504	304	493
5702	12/8/2014	7.1	29.5	1.2	694	1858	185	364
5702	1/19/2015	7.0	21.5	0.9	1085	2105	100	223
5702	2/3/2015	6.9	25.5	0.8	5025	6605	246	423
5702	2/16/2015	7.1	22.4	1.1	379	1723	88	259
5702	2/23/2015	7.0	20.5	0.7	907	2103	90	145
5702	3/3/2015	7.0	22.7	1.0	374	1758	121	199
5702	3/16/2015	7.2	35.4	1.7	401	1833	183	292
5702	4/14/2015	7.1	30.6	1.5	399	9707	1272	2562
5702	5/18/2015	6.8	18.5	0.5	825	2362	126	300
5702	5/25/2015	7.1	28.1	1.5	2	2426	515	945
5702	6/9/2015	6.6	32.4	0.9	2	3284	1624	2329
5702	9/9/2015	5.4		0.1	16	1966	1157	1857
5702	9/15/2015	5.1	51.9	0.1	70	5931	1700	3206
5702	11/9/2015	7.1	31.3	1.0	1736	3696	153	378
5702	12/14/2015	7.2	33.5	1.7	1157	2647	115	204
5702	1/18/2016	7.2	35.7	2.2	966	2316	97	167
5702	1/28/2016	7.1	20.8	0.7	1032	2584	119	276
5702	2/15/2016	7.4	29.9	1.4	814	1981	75	140
5702	3/14/2016	7.5	32.1	1.7	919	1858	79	136
5702	4/11/2016	7.8	33.4	2.1	705	1492	91	125
5702	5/16/2016	7.8	45.5	1.9	1779	3229	93	127
5702	6/20/2016	7.9	46.2	1.6	1433	2493	85	187
5702	11/7/2016	7.5	46.8	1.3	2127	3279	48	103
5702	11/15/2016	6.7	21.4	0.4	1685	2810	101	183
5702	11/22/2016	6.7	30.0	0.6	6677	8564	64	183
5702	12/12/2016	6.9	31.4	1.0	2632	3488	34	103
5702	1/10/2017	7.1	25.2	1.1	745	1311	78	129
5702	1/30/2017	6.9	29.6	1.3	2	1251	188	355
5702	2/13/2017	6.5	48.0	1.4	2178	3665	395	839
5702	3/14/2017	6.9	31.7	1.0	3494	4454	79	163
5702	3/20/2017	7.1	35.4	1.4	1150	3773	154	293
5702	4/10/2017	7.3	41.9	2.5	1837	3312	505	551
5702	6/12/2017	7.4	30.0	1.6	2073	3395	344	659
5702	9/12/2017	6.8	48.5	2.3	2	2313	1601	1721
5702	10/16/2017	7.0	30.5	1.3	1265	3245	986	1031
5702	11/15/2017	6.8	30.2	1.0	41	1373	810	872
5702	12/11/2017	7.1	24.8	1.1	508	1706	359	471
5702	1/15/2018	7.2	25.3	1.4	325	1201	284	340
5702	2/12/2018	7.3	25.5	1.3	586	1479	238	318
5702	3/19/2018	7.2	21.0	1.2	281	1083	295	355

5702	3/27/2018	6.8	19.9	0.8	802	1542	158	231
5702	4/16/2018	7.1	27.3	1.4	651	1823	303	424
5702	5/14/2018	7.2	37.7	2.5	188	4468	3673	3713
5702	11/5/2018	7.1	39.3	0.9	22	1196	895	1080
5702	12/10/2018	7.2	25.3	0.6	1359	2273	114	236
5702	1/14/2019	7.1	34.9	1.1	3524	4780	105	279
5702	2/11/2019	6.5	14.4	0.4	1015	1950	65	140
5702	3/5/2019	7.0	22.0	0.9	51	855	198	253
5702	3/18/2019	6.9	21.0	0.6	3947	4471	86	242
5702	4/24/2019	7.1	49.2	3.5	378	3869	1545	1731
5702	5/21/2019	7.5	40.7	2.4	20	2792	292	393
5702	8/12/2019	7.1	38.1	0.8	1968	3565	154	236
5702	9/17/2019	5.5	62.4	0.5	0	2894	4901	6622
5702	10/14/2019	6.9	20.3	0.5	252	2092	150	615
5702	11/11/2019	7.2	26.4	1.0	217	1008	269	342
5702	12/9/2019	7.1	25.7	0.9	1553	2783	188	337
5702	1/13/2020	7.4	26.3	1.6	378	1356	93	128
5702	2/11/2020	7.1	22.4	0.8	1065	2172	237	450
5702	3/9/2020	7.0	22.6	1.0	1846	2789	206	357
5702	4/6/2020	6.7	38.2	1.8	232	6935	2392	2527
5702	5/4/2020	7.2	20.0	1.0	352	1792	215	351
5702	5/18/2020	7.6	32.6	2.4	17	1590	457	688
5702	6/8/2020	7.4	44.4	3.6	18	4181	408	1026
5702	7/6/2020	6.3	31.8	0.7	6	3834	795	1178
5702	12/9/2020	6.0		0.6	2	930	1028	1185
5702	1/11/2021		24.2		989	2157	151	200
5702	1/26/2021		25.0		1902	3313	170	268
5702	2/8/2021		27.5		2	1152	316	395
5702	2/22/2021		20.2		1562	2499	140	235
5702	3/9/2021		31.9		1376	2344	163	237
5702	3/29/2021		44.0		0	1515	822	856
5702	4/19/2021		58.3		3	1660	739	1319
5702	5/17/2021		38.4		20	3226	360	1463
5702	6/7/2021		34.1		1406	3204	2030	2562
5702	10/11/2021		40.8		0	1375	1458	1590
5702	11/10/2021		33.7		0	1162	303	412
5702	11/22/2021		57.3		358	1748	1302	1519
5702	12/6/2021		85.0		229	2096	2163	2562
5702	12/14/2021		30.3		159	1858	229	481
5702	1/17/2022		27.8		1825	3093	186	252
5702	1/31/2022		19.7		2266	3475	122	189
5702	2/14/2022		19.5		1144	3126	232	529
5702	2/28/2022		25.2		3020	3994	146	219
5702	3/15/2022		31.4		1344	2479	317	411
5702	4/19/2022		32.4		33	2209	350	469
5702	6/7/2022		43.6		10	2532		
5702	10/10/2022		71.0		14	3874	480	3101
5702	11/8/2022		46.8		1001	2661	293	425
5702	11/29/2022		29.5		1477	3024	34	194
5702	12/13/2022		45.8		221	1884	807	1036

5712	3/27/2012	6.9	20.1	0.8	1893	2515	20	48
5712	4/2/2012	6.8	18.4	0.6	1391	2169	14	46
5712	4/10/2012	6.9	19.4	0.9	1214	1965	17	42
5712	5/15/2012	6.8		0.5	0	779	5	28
5712	6/19/2012	6.5		0.3	406	1599	35	117
5712	7/10/2012	6.9	23.2	1.3	401	2025	51	108
5712	7/30/2012	6.8	23.7	0.9	141	1980	65	162
5712	7/31/2012	6.7	15.8	0.8	62	1839	52	122
5712	8/2/2012	7.0	20.2	1.1	65	1742	48	105
5712	8/20/2012	7.1		2.2	139	4276	18	559
5712	9/10/2012	6.8		1.0	654	2338	37	80
5712	10/16/2012	6.3		0.3	761	2323	67	204
5712	10/18/2012	6.4	10.0	0.3	463	1770	25	83
5712	10/23/2012	6.3	10.2	0.4	442	1672	26	76
5712	11/12/2012	6.8		0.7	661	1934	50	116
5712	12/12/2012	6.6	14.3	0.6	574	1686	27	71
5712	1/16/2013	6.6	15.7	0.7	480	1411	23	58
5712	2/11/2013	6.7	16.9	0.8	337	1353	23	55
5712	3/18/2013	6.8		1.6	589	1666	39	87
5712	4/10/2013	6.9	26.2	1.0	677	1734	39	103
5712	4/16/2013	6.3		0.2	650	1408	32	133
5712	4/18/2013	6.4	7.9	0.2	658	1525	27	121
5712	4/22/2013	6.5	9.4	0.3	436	1341	41	19
5712	4/29/2013	6.9	14.1	0.6	363	1354	21	60
5712	5/15/2013	7.2	28.6	1.2	250	1821	37	142
5712	6/10/2013	7.2		1.9	174	2342	30	106
5712	7/2/2013	7.6	51.1	2.4	8	1665	23	68
5712	7/16/2013	8.3	50.7	2.0	40	1497	15	51
5712	8/6/2013	7.4		2.5	13	1070	30	65
5712	9/18/2013	7.2	53.6	1.7	219	1569	36	94
5712	10/14/2013	7.1	51.7	1.4	740	1865	21	70
5712	10/29/2013	6.7	52.5	0.9	4630	6172	40	129
5712	11/4/2013	6.8	47.1	0.9	2860	4283	32	96
5712	11/12/2013	6.7	25.9	0.7	2201	3129	32	93
5712	12/11/2013	6.7	17.9	0.4	1239	1968	17	49
5712	1/14/2014	6.6	14.8	0.4	704	1774	18	45
5712	2/11/2014	6.6	12.7	0.3	953	1864	24	67
5712	3/3/2014	6.9	18.8	0.6	751	1619	19	53
5712	3/10/2014	7.1	19.5	0.8	676	1466	10	43
5712	4/1/2014	7.0	18.8	0.8	820	1581	6	38
5712	4/14/2014	7.0	15.6	0.6	634	1707	18	119
5712	5/13/2014	7.5	22.7	0.8	569	1549	10	60
5712	6/10/2014	7.2	44.9	2.4	26	1419	10	52
5712	7/14/2014	7.2	43.6	2.3	16	1108	27	82
5712	9/15/2014	7.1	49.3	2.5	17	787	36	78
5712	10/13/2014	6.4	18.3	0.3	1415	2902	15	68
5712	11/10/2014	6.7	18.3	0.5	725	1741	17	49
5712	12/8/2014	7.0	23.5	0.8	1052	1803	12	47
5712	1/19/2015	6.7	13.8	0.4	1119	1626	11	37
5712	2/3/2015	6.8	12.2	0.4	1062	1668	15	53

5712	2/16/2015	6.7	17.7	0.6	806	1489	10	28
5712	2/23/2015	6.7	17.4	0.5	1457	2186	14	44
5712	3/3/2015	6.9	17.7	0.6	941	1725	12	43
5712	3/16/2015	7.0	21.3	0.9	901	1592	11	39
5712	4/14/2015	6.9	21.4	1.0	628	1581	30	72
5712	5/18/2015	6.8	16.9	0.5	2352	3528	13	86
5712	5/25/2015	7.1	14.6	0.6	537	1459	7	42
5712	6/9/2015	7.2	16.0	0.8	481	1482	9	100
5712	7/14/2015	7.3	38.5	2.1	48	1095	23	57
5712	8/18/2015	7.5	49.2	2.7	12	858	10	37
5712	9/9/2015	6.5		0.3	183	1353	11	52
5712	9/15/2015	6.8	22.4	0.8	202	1502	18	68
5712	10/19/2015	7.1	47.4	2.3	242	1168	22	41
5712	11/9/2015	6.9	36.7	1.0	1463	2841	34	105
5712	12/14/2015	6.9	20.7	0.7	678	1735	11	38
5712	1/18/2016	6.7	34.5	1.4	889	1613	17	46
5712	1/28/2016	6.8	23.3	0.6	1524	2532	19	76
5712	2/15/2016	6.8	20.2	0.6	829	1483	11	35
5712	3/14/2016	6.8	22.5	0.8	804	3125	12	42
5712	4/11/2016	7.0	26.2	1.2	642	1607	16	43
5712	5/16/2016	7.4	40.3	1.7	613	1862	39	66
5712	6/20/2016	7.3	21.7	0.9	333	1708	26	100
5712	9/13/2016	7.2	44.8	2.3	15	544	17	57
5712	10/10/2016	7.5	57.4	2.4	12	607	9	34
5712	11/7/2016	6.8	32.3	0.5	2856	4622	7	47
5712	11/15/2016	6.5	23.7	0.3	3818	4699	25	84
5712	11/22/2016	6.4	17.6	0.3	1799	3087	9	54
5712	12/12/2016	6.7	31.6	0.7	1857	2897	6	36
5712	1/10/2017	6.7	38.4	0.9	2421	3686	4	53
5712	1/30/2017	6.8	21.0	0.6	1265	1781	9	45
5712	2/13/2017	7.1	32.0	1.3	1517	2808	13	42
5712	3/14/2017	6.8	20.6	0.5	1537	2307	6	41
5712	3/20/2017	7.0	20.9	0.7	1470	2175	8	55
5712	4/10/2017	7.3	27.2	1.0	1046	1957	15	45
5712	5/15/2017	7.7	44.8	1.9	1117	1673	9	40
5712	6/12/2017	7.6	58.4	2.9	206	1299	14	49
5712	7/18/2017	7.5	91.4	4.8	1619	27402	1153	1502
5712	8/14/2017	7.3	43.6	2.1	16	895	47	122
5712	9/12/2017	7.3	39.4	1.1	406	1549	19	74
5712	10/16/2017	6.4	16.6	0.3	1170	2437	10	44
5712	11/15/2017	6.7	21.3	0.6	1096	2164	10	49
5712	12/11/2017	6.9	20.2	0.5	1296	2281	13	44
5712	1/15/2018	6.8	21.4	0.7	1663	1816	10	38
5712	2/12/2018	6.9	21.1	0.7	1237	1958	10	28
5712	3/19/2018	6.8	20.5	0.6	934	1594	10	29
5712	3/27/2018	6.7	19.0	0.5	1779	2387	10	39
5712	4/16/2018	6.8	18.0	0.6	761	1408	11	35
5712	5/14/2018	7.2	33.9	1.5	524	1308	12	36
5712	6/11/2018	7.6	45.4	2.7	12	1026	20	52
5712	8/13/2018	6.7	94.6	0.5	1653	3243	23	123

5712	9/10/2018	7.2	54.6	1.9	43	497	6	23
5712	10/15/2018	7.2	79.2	1.3	553	1013	3	28
5712	11/5/2018	7.4	50.1	0.6	3439	4453	8	36
5712	12/10/2018	5.9	23.3	0.2	3735	4916	11	46
5712	1/14/2019	6.2	27.6	0.4	2140	2962	6	24
5712	2/11/2019	6.1	17.2	0.1	3177	4548	14	50
5712	3/5/2019	6.7	26.8	0.5	1823	2579	8	30
5712	3/18/2019	6.4	16.0	0.2	2195	2958	17	57
5712	4/24/2019	7.3	50.5	1.4	1678	2204	3	21
5712	5/21/2019	7.2	56.2	2.0	270	1099	3	28
5712	5/27/2019	6.9	63.7	3.8	308	9381	452	520
5712	7/8/2019	7.1	70.2	1.4	5	743	9	34
5712	8/12/2019	6.9	58.8	0.6	2840	4899	44	147
5712	9/17/2019	7.0	62.6	1.5	2255	3333	15	79
5712	10/14/2019	6.7	47.1	1.0	1120	2407	39	175
5712	11/11/2019	6.5	24.1	0.6	1403	2181	14	49
5712	12/9/2019	6.4	18.1	0.4	1894	2898	17	61
5712	1/13/2020	6.9	26.4	0.9	1613	2012	15	39
5712	2/11/2020	6.7	25.9	0.7	2772	3718	29	99
5712	3/9/2020	6.7	19.5	0.5	2335	3004	14	52
5712	4/6/2020	7.1	30.6	1.1	1132	2109	14	43
5712	5/4/2020	7.0	19.9	0.6	1807	2644	17	66
5712	5/18/2020	7.4	29.5	1.1	779	1618	12	45
5712	6/8/2020	7.4	44.3	2.0	1180	1837	11	39
5712	7/6/2020	7.5	56.0	2.1	232	1521	25	83
5712	9/15/2020	7.1		2.1	7	376	11	24
5712	12/9/2020	6.8		0.6	3164	4006	13	53
5712	1/11/2021		21.4		1309	1992	9	31
5712	1/26/2021		20.7		1695	2463	13	37
5712	2/8/2021		30.4		1863	2664	13	32
5712	2/22/2021		38.0		2322	2981	32	59
5712	3/9/2021		26.6		1353	2367	35	115
5712	3/29/2021		28.6		1490	1762	16	42
5712	4/19/2021		47.0		1213	1826	6	27
5712	5/17/2021		31.3		1219	2041	16	74
5712	6/7/2021		25.8		862	1598	6	38
5712	10/11/2021		37.8		1034	2038	15	46
5712	11/10/2021		25.5		1774	2811	18	68
5712	11/22/2021		29.4		1163	2300	11	39
5712	12/6/2021		48.6		1379	2449	14	53
5712	12/14/2021		19.0		704	1610	17	56
5712	1/17/2022		20.2		1143	2028	17	46
5712	1/31/2022		17.9		2443	3497	16	60
5712	2/14/2022		20.1		956	2107	40	127
5712	2/28/2022		18.9		2055	2940	9	39
5712	3/15/2022		27.5		1618	2375	9	35
5712	4/19/2022		25.5		1523	2510	9	49
5712	5/16/2022		52.6		1787	2675		
5712	6/7/2022		32.5		2759	3522		
5712	10/10/2022		102.0		181	885	6	40

5712	11/8/2022		10.9		794	1492	11	29
5712	11/29/2022		29.5		4296	5518	17	91
5712	12/13/2022		31.4		2192	3009	12	39
5719	4/16/2013	6.1		0.1	209	972	29	128
5719	4/18/2013	6.2	4.6	0.1	192	1024	32	133
5719	4/22/2013	6.3	5.3	0.2	116	931	35	87
5719	4/29/2013	6.6	7.7	0.4	73	1025	23	73
5719	5/15/2013	6.9	19.5	1.0	72	1710	58	125
5719	6/10/2013	6.7		1.6	118	3863	129	378
5719	7/16/2013	6.6	36.3	2.1	3	3468	71	149
5719	9/18/2013	6.7	46.5	2.0	213	3412	108	268
5719	10/14/2013	6.7	59.0	1.9	3927	5565	34	88
5719	10/29/2013	6.2	47.9	0.4	5330	7183	68	192
5719	11/4/2013	6.3	36.7	0.5	3291	4791	40	116
5719	11/12/2013	6.3	13.1	0.3	990	1825	16	54
5719	12/11/2013	6.4	11.1	0.2	556	1387	17	50
5719	1/14/2014	6.3	7.8	0.2	320	1063	17	36
5719	2/11/2014	6.3	7.7	0.2	411	1134	21	63
5719	3/3/2014	6.5	11.7	0.3	314	943	21	60
5719	3/10/2014	6.7	11.5	0.4	252	1010	14	48
5719	4/1/2014	6.7	10.6	0.4	231	1027	20	60
5719	4/14/2014	6.7	9.0	0.3	189	947	16	51
5719	5/13/2014	6.8	13.6	0.5	371	1408	18	117
5719	6/10/2014	6.9	37.3	2.0	44	1675	53	97
5719	10/13/2014	6.3	13.4	0.2	657	1783	17	62
5719	11/10/2014	6.4	10.5	0.2	282	1231	14	41
5719	12/8/2014	6.8	13.6	0.4	427	1393	12	60
5719	1/19/2015	6.4	8.6	0.2	447	1126	6	35
5719	2/3/2015	6.2	7.1	0.1	405	1066	9	36
5719	2/16/2015	6.5	9.8	0.3	300	1023	11	29
5719	2/23/2015	6.5	9.6	0.2	501	1204	9	38
5719	3/3/2015	6.7	10.4	0.3	414	1199	14	47
5719	3/16/2015	6.8	12.2	0.5	362	1162	14	38
5719	4/14/2015	6.7	12.1	0.5	327	1287	26	58
5719	5/18/2015	6.6	11.7	0.3	1906	3493	19	92
5719	5/25/2015	6.8	9.3	0.4	462	1418	10	40
5719	6/9/2015	6.9	10.3	0.5	416	1371	9	38
5719	7/14/2015	7.0	25.6	1.4	112	1497	52	96
5719	8/18/2015	7.0	37.4	1.7	262	1973	66	101
5719	9/9/2015	6.1		0.1	88	1305	13	56
5719	9/15/2015	6.4	10.6	0.3	94	1543	24	94
5719	10/19/2015	6.6	30.0	1.5	240	1801	54	105
5719	11/9/2015	6.6	21.1	0.6	984	2606	52	152
5719	12/14/2015	6.7	10.1	0.4	278	1338	12	48
5719	1/18/2016	6.6	19.5	0.8	343	1232	21	49
5719	1/28/2016	6.7	15.2	0.3	699	1831	26	96
5719	2/15/2016	6.6	10.9	0.3	337	1104	13	42
5719	3/14/2016	6.7	12.5	0.4	364	1173	18	53
5719	4/11/2016	6.8	15.8	0.8	298	1203	27	68
5719	5/16/2016	7.1	31.3	1.4	272	1561	58	118

5719	6/20/2016	7.0	16.0	0.7	276	1435	31	92
5719	11/7/2016	6.4	16.2	0.3	1089	2240	8	54
5719	11/15/2016	6.4	13.0	0.1	1263	2209	24	76
5719	11/22/2016		8.8					
5719	12/12/2016	6.7	15.4	0.3	358	1201	6	38
5719	1/10/2017	6.6	17.7	0.5	265	1073	9	46
5719	1/30/2017	6.7	12.1	0.4	216	974	14	56
5719	2/13/2017	7.0	17.2	0.8	182	1059	17	51
5719	3/14/2017	6.6	11.2	0.3	236	965	13	59
5719	3/20/2017	6.8	12.0	0.4	239	959	11	41
5719	4/10/2017	7.1	17.0	0.7	165	1050	23	65
5719	5/15/2017	7.7	34.6	1.5	54	1021	43	75
5719	6/12/2017	7.6	50.0	1.9	8	1262	38	81
5719	9/12/2017	7.1	30.2	0.8	633	2043	25	78
5719	10/16/2017	6.3	9.9	0.2	372	1642	10	43
5719	11/15/2017	6.5	10.9	0.3	290	1385	9	44
5719	12/11/2017	6.8	10.0	0.3	285	1239	10	35
5719	1/15/2018	6.9	12.8	0.4	237	1021	11	32
5719	2/12/2018	6.8	11.9	0.4	261	1030	10	32
5719	3/19/2018	6.7	12.1	0.4	247	1077	13	81
5719	3/27/2018	6.5	8.4	0.2	288	925	12	43
5719	4/16/2018	6.7	11.3	0.4	207	866	15	43
5719	5/14/2018	7.4	21.1	1.1	74	885	26	59
5719	6/11/2018	7.7	50.0	2.8	3	816	31	61
5719	8/13/2018	6.7	111.8	0.3	1807	3386	15	108
5719	9/10/2018	7.3	72.8	0.8	1	348	0	15
5719	10/15/2018	7.2	86.2	0.6	2283	2969	1	17
5719	11/5/2018	6.6	38.2	0.3	3599	5283	10	47
5719	12/10/2018	6.3	14.6	0.1	1821	2944	11	47
5719	1/14/2019	6.6	17.4	0.2	1232	2139	7	21
5719	2/11/2019	6.0	13.6	0.1	1768	3082	14	47
5719	3/5/2019	6.5	14.8	0.2	667	1443	8	35
5719	3/18/2019	6.2	9.5	0.1	825	1571	13	44
5719	4/24/2019	7.3	33.1	1.0	1166	2129	11	38
5719	5/21/2019	7.3	43.9	1.5	453	1249	13	41
5719	5/27/2019	7.0	57.1	2.2	29	849	11	42
5719	8/12/2019	6.5	40.7	0.4	2797	4790	53	168
5719	9/17/2019	6.8	41.2	0.9	451	1589	29	83
5719	10/14/2019	6.9	29.5	0.7	569	2102	136	304
5719	11/11/2019	6.6	13.9	0.3	548	1382	18	61
5719	12/9/2019	6.5	10.1	0.2	544	1545	20	60
5719	1/13/2020	6.8	16.0	0.5	377	1108	20	50
5719	2/11/2020	6.7	13.2	0.4	731	1898	42	119
5719	3/9/2020	6.5	9.4	0.3	458	1250	15	53
5719	4/6/2020	7.0	18.2	0.7	351	1268	31	75
5719	5/4/2020	6.7	10.9	0.3	512	1441	20	79
5719	5/18/2020	7.1	17.9	0.8	215	1116	20	57
5719	6/8/2020	7.4	28.1	1.5	35	1018	28	69
5719	7/6/2020	7.4	48.8	1.7	273	1644	34	134
5719	12/9/2020	6.5		0.3	3759	3847	12	59

5719	1/11/2021		13.3		793	1558		11	36
5719	1/26/2021		11.2		807	1530		9	34
5719	2/8/2021		16.9		778	1617		15	41
5719	2/22/2021		24.6		1081	1935		36	77
5719	3/9/2021		15.5		613	1277		8	34
5719	3/29/2021		16.4		464	1095		19	48
5719	4/19/2021		30.3		616	1312		15	41
5719	5/17/2021		19.0		672	1650		21	92
5719	6/7/2021		16.8		745	1593		10	52
5719	10/11/2021		21.0		196	1263		19	75
5719	11/10/2021		14.7		787	1806		19	70
5719	11/22/2021		18.0		511	1589		11	41
5719	12/6/2021		29.3		492	1645		16	56
5719	12/14/2021		11.4		485	1464		17	56
5719	1/17/2022		12.2		641	1363		10	34
5719	1/31/2022		9.7		1010	1948		14	55
5719	2/14/2022		12.9		547	1387		22	67
5719	2/28/2022		9.7		746	1511		7	29
5719	3/15/2022		16.5		711	1487		17	51
5719	4/19/2022		15.0		719	1848		55	157
5719	5/16/2022		35.8		724	1520			
5719	6/7/2022		19.0		1095	2236			
5719	10/10/2022		118.9		123	1047		11	29
5719	11/8/2022		10.2		924	2001		39	91
5719	11/29/2022		17.8		1581	2684		20	58
5719	12/13/2022		18.7		870	1890		10	33
CW1in	1/14/2014		36.0						
CW1in	2/11/2014	6.8	30.5	1.5	2171	2823		29	56
CW1in	3/3/2014	7.1	39.4	2.0	1707	2524		18	39
CW1in	3/10/2014	7.1	43.4	2.4	1053	2372		17	35
CW1in	4/1/2014	7.0	43.3	2.5	2533	2877		5	52
CW1in	4/14/2014	7.1	38.6	2.1	2155	3032		34	65
CW1in	5/13/2014	7.0	35.1	2.1	1013	1856		39	85
CW1in	6/10/2014	7.1	59.0	3.8	1570	2833		65	122
CW1in	9/15/2014	7.8	52.9	3.8	112	443		27	58
CW1in	10/13/2014	6.6	30.0	1.4	2074	2414		42	96
CW1in	11/10/2014	6.9	36.3	2.0	1142	1729		19	37
CW1in	12/8/2014	7.0	39.3	2.4	1409	1802		17	40
CW1in	1/19/2015	6.8	26.7	1.5	931	1359		19	39
CW1in	2/3/2015	6.8	32.8	1.9	1929	2321		38	73
CW1in	2/16/2015	6.9	38.2	2.3	1511	2060		10	21
CW1in	2/23/2015	6.8	35.6	1.9	2589	2824		17	39
CW1in	3/3/2015	6.8	38.2	2.2	2166	2553		9	26
CW1in	3/16/2015	7.0	43.9	2.8	2207	2752		18	31
CW1in	4/14/2015	6.9	45.1	3.0	2388	2428		14	24
CW1in	5/18/2015	6.5	14.6	0.7	439	1243		26	99
CW1in	5/25/2015	6.9	35.8	2.5	681	1123		12	25
CW1in	6/9/2015	6.9	40.7	2.7	602	1012		15	23
CW1in	8/18/2015	7.0	64.3	4.2	694	936		36	49
CW1in	9/9/2015	6.8		4.0	1180	1709		33	56

CW1in	9/15/2015	7.4	61.5	4.4	805	1160	34	70
CW1in	10/19/2015	6.7	66.0	4.8	525	811	33	50
CW1in	11/9/2015	7.0	52.7	3.7	1275	1983	33	90
CW1in	12/14/2015	7.0	44.7	2.7	1398	2410	15	25
CW1in	1/18/2016	7.0	44.3	3.1	1082	1765	22	120
CW1in	1/28/2016	6.9	29.8	1.8	1599	2410	45	130
CW1in	2/15/2016	6.9	39.5	2.6	1387	1802	14	28
CW1in	3/14/2016	6.9	40.8	2.8	1065	4597	24	49
CW1in	4/11/2016	6.9	49.0	3.3	1610	2075	19	27
CW1in	5/16/2016	7.3	55.7	3.8	1136	1619	33	42
CW1in	6/20/2016	7.5	48.1	2.7	429	945	43	74
CW1in	7/19/2016	7.1		4.0	309	614	27	54
CW1in	9/13/2016	7.6	58.8	4.1	406	695	29	47
CW1in	10/10/2016	7.5	58.6	4.0	411	917	43	50
CW1in	11/7/2016	7.0	48.4	2.3	7589	8692	13	25
CW1in	11/15/2016	6.7	27.1	0.8	5598	7397	42	121
CW1in	11/22/2016	6.8	29.7	1.2	4919	6653	17	62
CW1in	12/12/2016	7.0	43.2	2.4	4837	5189	12	31
CW1in	1/10/2017	7.2	44.0	2.5	4351	4669	17	56
CW1in	1/30/2017	7.1	42.9	2.3	4238	5076	10	22
CW1in	2/13/2017	7.3	51.7	3.1	4622	4727	10	20
CW1in	3/14/2017	7.0	33.4	1.8	3852	4512	12	47
CW1in	3/20/2017	7.1	43.3	2.5	5286	5764	7	19
CW1in	4/10/2017	7.3	48.3	2.9	4805	5533	12	17
CW1in	5/15/2017	7.4	56.7	3.7	4994	5148	20	21
CW1in	6/12/2017	7.3	108.1	3.3	2915	3966	29	81
CW1in	10/16/2017	6.8	29.8	1.5	3402	4673	12	33
CW1in	11/15/2017	6.9	36.8	1.9	2778	4058	7	29
CW1in	12/11/2017	6.9	36.6	1.9	3742	4883	8	28
CW1in	1/15/2018	7.1	40.8	2.2	3483	4020	9	19
CW1in	2/12/2018	7.1	40.1	2.3	3280	3967	6	14
CW1in	3/19/2018	6.9	35.5	2.0	2536	3140	6	15
CW1in	3/27/2018	6.8	31.9	1.7	3277	3812	10	36
CW1in	4/16/2018	6.8	36.4	2.2	3525	3870	7	16
CW1in	5/14/2018	7.1	54.1	3.4	3195	3799	9	14
CW1in	6/11/2018	7.5	62.9	4.1	1588	1921	14	17
CW1in	11/5/2018	7.6	65.0	2.9	3613	3960	17	22
CW1in	12/10/2018	6.7	28.6	0.9	4602	5768	11	53
CW1in	1/14/2019	7.0	38.6	1.7	2844	3677	5	19
CW1in	2/11/2019	6.7	18.3	0.5	2756	4515	32	69
CW1in	3/5/2019	7.1	33.8	1.5	2903	4110	11	35
CW1in	3/18/2019	6.8	25.7	1.0	3704	4711	46	111
CW1in	4/24/2019	7.6	58.1	3.4	2887	3615	7	17
CW1in	5/21/2019	7.4	62.5	3.8	1911	2101	17	54
CW1in	7/8/2019	7.2	51.0	3.6	79	226	14	16
CW1in	8/12/2019	7.3	49.1	2.4	525	1378	53	118
CW1in	9/17/2019	7.6	66.5	3.8	2640	3144	17	26
CW1in	10/14/2019	7.0	41.5	2.1	3242	4939	39	262
CW1in	11/11/2019	7.1	35.9	2.3	3584	4306	6	33
CW1in	12/9/2019	7.0	31.2	1.5	4262	5156	15	64

CW1in	1/13/2020	7.2	47.6	2.8	4038	4615	10	31
CW1in	2/11/2020	7.0	37.3	2.0	5501	6615	25	103
CW1in	3/9/2020	7.3	31.9	1.6	4026	4830	40	79
CW1in	4/6/2020	7.3	50.8	3.1	4345	5224	11	26
CW1in	5/4/2020	7.1	32.0	1.8	4100	4936	13	57
CW1in	5/18/2020	7.5	50.8	3.2	4373	4943	11	28
CW1in	6/8/2020	7.4	61.1	4.0	3827	4348	9	19
CW1in	7/6/2020	8.0	60.6	3.5	666	1224	12	62
CW1in	9/15/2020	7.9		3.4	122	276	23	29
CW1in	12/9/2020	7.6		3.4	3948	4240	35	56
CW1in	1/11/2021		34.0		1729	2147	12	37
CW1in	1/26/2021		31.9		2158	2544	21	39
CW1in	2/8/2021		45.2		2173	2554	14	23
CW1in	2/22/2021		36.7		2743	3153	42	80
CW1in	3/9/2021		44.2		2134	2533	8	18
CW1in	3/29/2021		46.6		2272	2335	4	14
CW1in	4/19/2021		55.2		1806	2113	13	21
CW1in	5/17/2021		33.0		1293	1782	12	46
CW1in	6/7/2021		48.6		2504	2585	9	20
CW1in	10/11/2021		66.3		3659	4322	15	23
CW1in	11/10/2021		42.5		4680	5655	29	84
CW1in	11/22/2021		56.6		4349	5625	15	23
CW1in	12/6/2021		65.8		3011	3713	15	19
CW1in	12/14/2021		24.2		1245	2342	58	131
CW1in	1/17/2022		28.1		1587	2202	12	26
CW1in	1/31/2022		17.9		1101	1885	17	53
CW1in	2/14/2022		11.8		1912	3965	219	708
CW1in	2/28/2022		24.2		1109	2064	57	110
CW1in	3/15/2022		46.4		2309	3457	61	110
CW2in	11/4/2013	7.2	31.0	0.9	3510	5882	647	814
CW2in	11/12/2013	6.4	32.6	0.4	5536	6849	180	292
CW2in	12/11/2013	6.5	24.3	0.5	3342	4686	270	463
CW2in	1/14/2014	6.2	20.9	0.5	2034	3903	132	365
CW2in	2/11/2014	6.3	17.5	0.4	2916	3978	107	327
CW2in	3/3/2014	6.7	25.1	0.9	1582	3991	577	751
CW2in	3/10/2014	6.9	26.3	0.9	1647	3767	373	515
CW2in	4/1/2014	6.6	22.2	0.7	937	2878	0	117
CW2in	4/14/2014	6.6	22.5	0.6	1894	3187	89	201
CW2in	5/13/2014	6.6	21.9	0.8	691	1972	252	390
CW2in	6/10/2014	6.3	34.8	0.5	841	1465	71	123
CW2in	10/13/2014	6.0	38.8	0.3	5503	7799	141	350
CW2in	11/10/2014	6.4	41.2	0.5	2704	4052	127	231
CW2in	12/8/2014	6.5	43.9	0.5	2937	4210	173	312
CW2in	1/19/2015	6.2	31.8	0.5	4789	5728	62	146
CW2in	2/3/2015	6.2	20.1	0.3	4591	5200	57	186
CW2in	2/16/2015	6.5	25.7	0.5	2674	3639	58	129
CW2in	2/23/2015	6.2	26.9	0.4	5676	6550	126	298
CW2in	3/3/2015	6.4	24.8	0.5	2974	4392	130	224
CW2in	3/16/2015	6.6	26.1	0.6	2620	3573	54	109
CW2in	4/14/2015	6.5	29.3	0.6	4246	6903	42	82

CW2in	5/18/2015	6.2	27.7	0.4	7190	8896	185	462
CW2in	5/25/2015	6.6	21.4	0.5	3174	4622	21	120
CW2in	6/9/2015	6.4	22.5	0.6	2250	3313	37	108
CW2in	7/14/2015	6.3	38.2	0.4	881	1675	52	100
CW2in	9/9/2015	6.5		0.6	1269	3048	249	354
CW2in	9/15/2015	7.3	58.9	3.3	2034	11292	2697	3649
CW2in	11/9/2015	6.3	37.6	0.6	5365	7776	151	343
CW2in	12/14/2015	6.5	18.8	0.5	1405	2579	89	192
CW2in	1/18/2016	6.5	21.2	0.7	955	2489	48	94
CW2in	1/28/2016	6.4	19.3	0.5	3165	4759	111	295
CW2in	2/15/2016	6.3	19.1	0.5	2646	3520	66	160
CW2in	3/14/2016	6.4	20.6	0.6	2087	3330	62	191
CW2in	4/11/2016	6.5	24.7	0.9	1326	3021	109	264
CW2in	5/16/2016	6.8	46.6	1.1	2920	6311	272	370
CW2in	6/20/2016	7.0	48.5	1.2	3298	8655	817	1185
CW2in	11/7/2016	6.9	42.2	0.4	6193	8140	219	415
CW2in	11/15/2016	6.2	25.2	0.3	10493	12612	96	234
CW2in	11/22/2016	6.2	19.5	0.3	5374	7873	56	164
CW2in	12/12/2016	6.6	31.6	0.7	5496	5514	161	287
CW2in	1/10/2017	6.6	29.1	0.6	2660	4226	42	182
CW2in	1/30/2017	6.6	26.6	0.6	5813	7222	47	197
CW2in	2/13/2017	6.6	24.5	0.7	2871	4556	71	215
CW2in	3/14/2017	6.7	24.6	0.5	6599	8490	74	238
CW2in	3/20/2017	6.8	23.7	0.6	5199	6907	51	214
CW2in	4/10/2017	6.6	28.5	0.9	3334	4602	42	144
CW2in	5/15/2017	7.3	45.0	1.9	9	1954	238	513
CW2in	6/12/2017	6.9	9.5	0.3	589	1867	230	404
CW2in	9/12/2017	4.6	64.8	0.0	28	3584	5329	5688
CW2in	10/16/2017	6.3	24.6	0.5	3305	4872	82	206
CW2in	11/15/2017	6.6	20.6	0.4	1564	3068	55	150
CW2in	12/11/2017	6.5	18.1	0.4	1576	3106	65	162
CW2in	1/15/2018	6.6	17.5	0.5	1987	2342	64	211
CW2in	2/12/2018	6.8	18.2	0.5	1475	2402	99	214
CW2in	3/19/2018	6.5	16.5	0.5	1163	2120	79	153
CW2in	3/27/2018	6.4	18.3	0.5	1921	2791	103	197
CW2in	4/16/2018	6.6	18.5	0.6	886	1956	85	194
CW2in	5/14/2018	7.0	33.0	0.9	657	2058	129	236
CW2in	11/5/2018	5.1	110.7	0.0	3337	4583	31	290
CW2in	12/10/2018	6.2	51.7	0.3	11719	13736	112	230
CW2in	1/14/2019	6.2	35.0	0.4	3610	4731	43	102
CW2in	2/11/2019	6.0	22.7	0.2	7489	8858	90	167
CW2in	3/5/2019	6.5	21.2	0.3	3091	4286	129	234
CW2in	3/18/2019	6.2	21.3	0.2	7273	8183	113	265
CW2in	4/24/2019	6.8	51.5	0.4	6083	8021	74	159
CW2in	8/12/2019	6.8	54.8	0.9	2682	5947	722	1311
CW2in	9/17/2019	6.5	61.2	0.5	2465	4729	140	1266
CW2in	10/14/2019	6.7	30.8	1.0	725	4308	1122	1635
CW2in	11/11/2019	6.4	27.3	0.5	4743	6199	95	264
CW2in	12/9/2019	6.4	18.9	0.4	3834	5503	111	306
CW2in	1/13/2020	6.6	19.1	0.5	1439	2292	84	161

CW2in	2/11/2020	6.4	24.3	0.5	5102	7183	179	519
CW2in	3/9/2020	6.6	13.3	0.3	1643	2644	46	156
CW2in	4/6/2020	7.0	24.9	0.8	1326	2349	75	145
CW2in	5/4/2020	6.6	13.0	0.4	926	1972	69	198
CW2in	5/18/2020	6.9	22.1	0.8	725	987	150	217
CW2in	12/9/2020	7.0		1.2	14126	15516	344	1163
CW2in	1/11/2021		26.3		2542	3343	57	114
CW2in	1/26/2021		22.2		2728	4154	60	112
CW2in	2/8/2021		32.6		1823	4835	201	409
CW2in	2/22/2021		24.4		2656	3609	82	155
CW2in	3/9/2021		28.6		2424	3639	128	213
CW2in	3/29/2021		32.4		2068	2990	110	190
CW2in	4/19/2021		58.5		2228	3660	206	337
CW2in	5/17/2021		26.3		1138	3052	431	640
CW2in	6/7/2021		43.3		3992	4363	193	366
CW2in	11/10/2021		40.2		4480	9942	183	365
CW2in	11/22/2021		25.5		2115	3432	26	140
CW2in	12/6/2021		42.0		1735	3056	41	95
CW2in	12/14/2021		22.6		1131	5475	265	792
CW2in	1/17/2022		30.6		2173	4616	143	409
CW2in	1/31/2022		23.7		5104	6727	29	142
CW2in	2/14/2022		26.4		1039	7637	664	1952
CW2in	2/28/2022		18.2		3187	4294	33	119
CW2in	3/15/2022		37.6		1805	3497	55	266
CW3out	8/24/2014	7.6	79.3	6.0	12	8053	246	628
CW3out	9/15/2014	7.3	108.3	5.2	5	6628	69	194
CW3out	10/13/2014	6.4	21.2	0.5	1648	2883	24	84
CW3out	11/10/2014	6.8	19.4	0.6	750	1765	21	63
CW3out	12/8/2014	7.1	27.2	1.0	970	2123	12	63
CW3out	1/19/2015	6.7	14.8	0.4	1089	1734	11	42
CW3out	2/3/2015	6.6	13.1	0.4	1199	1803	17	56
CW3out	2/16/2015	6.8	19.4	0.7	762	1620	10	39
CW3out	2/23/2015	6.8	18.8	0.5	1488	2191	15	58
CW3out	3/3/2015	6.9	19.2	0.7	1012	1906	16	53
CW3out	3/16/2015	7.1	22.9	1.0	811	1704	15	52
CW3out	4/14/2015	7.2	22.9	1.1	511	1803	52	133
CW3out	5/18/2015	7.1	20.1	0.6	2540	3801	24	119
CW3out	5/25/2015	7.0	16.6	0.8	594	1750	26	78
CW3out	6/9/2015	7.0	17.2	0.7	222	1945	51	142
CW3out	7/14/2015	7.2	37.5	2.0	13	2163	95	223
CW3out	8/18/2015	7.5	47.2	2.7	9	2729	114	259
CW3out	9/9/2015	6.6		0.4	139	1498	21	95
CW3out	9/15/2015	7.0	23.2	1.8	22	1553	54	149
CW3out	10/19/2015	7.2	52.0	2.8	129	1922	40	85
CW3out	11/9/2015	7.0	39.9	1.2	1816	3578	32	148
CW3out	12/14/2015	7.0	22.7	0.8	601	1959	21	57
CW3out	1/18/2016	6.8	36.3	1.6	721	1937	24	60
CW3out	1/28/2016	7.0	25.0	0.8	1500	2842	31	96
CW3out	2/15/2016	6.9	22.4	0.8	779	1601	18	48
CW3out	3/14/2016	7.0	24.8	0.9	812	1604	18	46

CW3out	4/11/2016	7.3	26.6	1.2	607	1715	31	69
CW3out	5/16/2016	7.8	39.4	2.0	374	1575	16	63
CW3out	6/20/2016	7.2	28.0	1.2	376	2216	61	154
CW3out	7/19/2016	8.9	45.4	2.2	758	2138	70	113
CW3out	8/16/2016	9.5	52.3	1.8	34	1438	26	57
CW3out	9/13/2016	7.5	69.1	3.4	2313	3134	23	45
CW3out	10/10/2016	7.7	69.7	3.4	3314	6677	237	261
CW3out	11/7/2016	6.8	35.4	0.6	3223	4794	10	80
CW3out	11/15/2016	6.4	25.8	0.3	3719	4861	34	110
CW3out	11/22/2016	6.5	18.9	0.3	2000	3250	20	64
CW3out	12/12/2016	6.9	33.3	0.9	2156	2826	26	62
CW3out	1/10/2017	6.9	32.2	1.1	1177	2082	13	45
CW3out	1/30/2017	6.9	23.6	0.7	1160	1955	8	46
CW3out	2/13/2017	7.1	35.7	1.6	1466	2629	19	53
CW3out	3/14/2017	6.9	22.8	0.6	1565	2713	11	52
CW3out	3/20/2017	7.0	22.7	0.8	1469	2443	17	63
CW3out	4/10/2017	7.3	29.2	1.2	904	1752	32	67
CW3out	5/15/2017	8.9	44.3	1.9	17	1187	11	70
CW3out	6/12/2017	7.2	62.7	3.1	33	2859	45	153
CW3out	7/18/2017	7.7	64.8	2.9	211	3188	86	168
CW3out	8/14/2017	8.5	63.2	2.2	1725	2389	64	102
CW3out	9/12/2017	7.0	60.5	1.6	148	1904	86	146
CW3out	10/16/2017	6.4	18.5	0.4	1202	2748	40	84
CW3out	11/15/2017	6.7	22.5	0.7	1010	2162	27	62
CW3out	12/11/2017	6.6	20.1	0.6	1157	2306	27	70
CW3out	1/15/2018	6.9	23.0	0.9	1393	1803	24	53
CW3out	2/12/2018	6.8	23.0	0.8	1207	1949	22	45
CW3out	3/19/2018	6.6	23.0	0.7	916	1654	21	47
CW3out	3/27/2018	6.6	22.0	0.6	2300	2976	21	61
CW3out	4/16/2018	6.8	19.7	0.7	730	1474	29	62
CW3out	5/14/2018	7.3	32.5	1.4	42	1043	51	103
CW3out	8/13/2018	7.1	85.0	1.0	1465	3568	83	179
CW3out	9/10/2018	7.5	78.1	3.2	29	6292	57	101
CW3out	10/15/2018	7.1	81.0	2.6	0	1264	179	257
CW3out	11/5/2018	7.2	53.4	0.8	3158	4872	40	96
CW3out	12/10/2018	6.5	24.0	0.2	4006	4399	20	61
CW3out	1/14/2019	6.7	28.9	0.5	2171	2986	11	32
CW3out	2/11/2019	6.2	16.9	0.2	2698	4017	18	58
CW3out	3/5/2019	6.8	30.8	0.5	2025	2154	15	46
CW3out	3/18/2019	6.5	16.3	0.3	1959	2627	22	66
CW3out	4/24/2019	7.5	47.8	1.5	1088	1977	19	80
CW3out	5/21/2019	7.5	58.0	2.1	121	1628	15	134
CW3out	5/27/2019	7.1	61.6	2.8	27	2630	218	434
CW3out	7/8/2019	7.4	76.9	2.3	507	2725	152	204
CW3out	8/12/2019	6.8	57.6	0.7	2611	4588	62	172
CW3out	10/14/2019	6.8	38.7	1.2	499	2132	64	385
CW3out	11/11/2019	6.8	27.7	0.7	1995	2476	31	77
CW3out	12/9/2019	6.7	19.9	0.5	1983	3159	27	83
CW3out	1/13/2020	7.1	29.1	1.1	1395	1929	22	49
CW3out	2/11/2020	6.9	26.2	0.7	2887	3908	41	146

CW3out	3/9/2020	6.8	20.2	0.6	2046	2858	24	74
CW3out	4/6/2020	7.1	32.4	1.3	1056	2041	29	60
CW3out	5/4/2020	7.1	22.2	0.8	1948	2849	25	90
CW3out	5/18/2020	7.6	31.4	1.3	672	1548	24	52
CW3out	6/8/2020	7.3	46.5	2.3	198	1308	32	86
CW3out	7/6/2020	7.4	50.0	2.0	45	2025	194	421
CW3out	9/15/2020	7.3		2.9	1	1244	345	389
CW3out	12/9/2020	6.7		0.7	2759	3613	31	112
CW3out	1/11/2021		23.5		1361	2076	17	40
CW3out	1/26/2021		22.2		1705	2574	19	47
CW3out	2/8/2021		29.9		1482	2306	17	40
CW3out	2/22/2021		40.3		1891	2839	37	77
CW3out	3/9/2021		27.4		1389	1978	11	43
CW3out	3/29/2021		30.3		1223	1741	21	55
CW3out	4/19/2021		46.6		370	1283	48	99
CW3out	5/17/2021		33.6		220	1271	43	125
CW3out	6/7/2021		25.7		445	1413	61	109
CW3out	10/11/2021				619	1707	61	123
CW3out	11/10/2021		28.8		1876	2949	24	86
CW3out	11/22/2021		31.8		981	2276	26	64
CW3out	12/6/2021		50.2		955	1898	33	61
CW3out	12/14/2021		19.6		721	1861	17	74
CW3out	1/17/2022		22.4		1189	2204	27	82
CW3out	1/31/2022		16.2		2520	3619	20	68
CW3out	2/14/2022		24.4		1028	1855	34	94
CW3out	2/28/2022		20.4		2127	2956	15	43
CW3out	3/15/2022		30.6		1732	2507	20	44
CW3out	4/19/2022		27.3		1476	2400	13	56
CW3out	5/16/2022		52.6		164	1490		
CW3out	6/7/2022		32.6		2180	3543		
CW3out	10/10/2022		91.2		0	1810	228	413
CW3out	11/8/2022		90.8		0	941	26	100
CW3out	11/29/2022		31.3		3659	5201	16	62
CW3out	12/13/2022		30.9		1590	2591	15	44
LF1in	11/4/2013	7.4	59.4	2.8	2096	3258	10	278
LF1in	11/12/2013	7.1	46.1	1.8	4744	5711	28	80
LF1in	12/11/2013	6.9	30.9	1.6	1938	2514	22	49
LF1in	1/14/2014	6.8	35.7	1.7	1946	2928	35	65
LF1in	2/11/2014	6.8	28.2	1.1	2751	3748	43	93
LF1in	3/3/2014	7.3	39.6	1.8	1789	2912	12	30
LF1in	3/10/2014	8.0	42.9	2.2	1015	2049	11	35
LF1in	4/1/2014	7.7	45.8	2.3	1936	3514	0	385
LF1in	4/14/2014	7.7	38.8	1.9	1668	2618	20	74
LF1in	5/13/2014	8.6	48.7	2.4	50	1311	17	126
LF1in	6/10/2014	8.0	54.1	3.5	97	1953	67	254
LF1in	10/13/2014	6.7	40.1	1.4	3245	5538	29	119
LF1in	11/10/2014	7.1	44.7	2.1	2146	3340	41	62
LF1in	12/8/2014	7.5	52.0	2.8	2065	2275	10	32
LF1in	1/19/2015	6.8	26.0	1.3	998	1564	29	57
LF1in	2/3/2015	6.8	30.1	1.4	1909	3063	58	110

LF1in	2/16/2015	6.9	37.3	1.9	1693	2317	8	24
LF1in	2/23/2015	6.9	36.2	1.7	3378	3465	15	52
LF1in	3/3/2015	7.2	38.5	2.1	1918	2371	10	31
LF1in	3/16/2015	7.3	41.7	2.4	1588	2464	36	61
LF1in	4/14/2015	8.0	41.4	2.5	880	1608	13	40
LF1in	5/18/2015	7.7	44.8	2.5	143	1112	16	80
LF1in	5/25/2015	7.5	31.8	2.1	55	1278	20	114
LF1in	6/9/2015	8.0	32.1	2.2	15	951	13	63
LF1in	7/14/2015	7.2	47.4	3.5	0	841	11	58
LF1in	8/18/2015	7.2	56.2	3.6	2	675	10	31
LF1in	9/9/2015	8.0		3.4	1	550	5	23
LF1in	9/15/2015	7.6	47.8	2.9	5	823	9	54
LF1in	10/19/2015	7.5	57.9	4.2	0	393	1	10
LF1in	11/9/2015	7.9	55.6	3.9	0	465	11	44
LF1in	12/14/2015	7.1	44.0	2.8	845	1957	14	42
LF1in	1/18/2016	6.9	47.8	3.4	1192	2024	9	24
LF1in	1/28/2016	7.0	33.7	1.9	1876	2521	25	110
LF1in	2/15/2016	6.9	39.1	2.5	1287	1885	13	43
LF1in	3/14/2016	7.2	36.0	2.3	466	2096	23	147
LF1in	4/11/2016	7.5	45.9	3.3	587	1668	7	35
LF1in	5/16/2016	7.8	44.2	3.1	2	1148	3	83
LF1in	6/20/2016	7.6	47.0	3.1	5	3285	7	441
LF1in	7/19/2016	6.9		3.7	2	2504	22	285
LF1in	9/13/2016	7.1	54.0	3.8	4	973	6	56
LF1in	10/10/2016	7.3	54.3	3.2	2	854	6	57
LF1in	11/7/2016	7.1	52.9	1.9	11893	14121	12	129
LF1in	11/15/2016	6.9	33.8	1.1	5787	7011	34	116
LF1in	11/22/2016	6.8	30.2	1.2	4928	6825	25	113
LF1in	12/12/2016	7.1	44.4	2.2	3477	4104	6	29
LF1in	1/10/2017	7.0	51.0	2.9	3575	3990	1	19
LF1in	1/30/2017	7.0	41.1	2.1	4316	5099	6	28
LF1in	2/13/2017	7.2	56.4	3.2	3693	4102	3	21
LF1in	3/14/2017	7.1	34.0	1.8	2810	3581	20	62
LF1in	3/20/2017	7.3	42.0	2.4	3837	5450	56	161
LF1in	4/10/2017	8.0	42.1	2.6	1869	2576	6	36
LF1in	5/15/2017	7.6	44.7	2.5	0	1094	12	97
LF1in	6/12/2017	7.4	51.6	3.4	1	1895	18	147
LF1in	9/12/2017	7.1	36.1	2.0	0	1107	7	52
LF1in	10/16/2017	7.0	30.5	1.5	3583	4999	14	51
LF1in	11/15/2017	7.0	35.7	1.8	2534	3867	26	74
LF1in	12/11/2017	7.0	33.6	1.6	3153	4679	54	126
LF1in	1/15/2018	7.0	38.4	2.0	3498	4342	9	37
LF1in	2/12/2018	7.0	36.8	1.7	2972	4090	11	40
LF1in	3/19/2018	6.9	32.8	1.8	1977	2625	8	34
LF1in	3/27/2018	6.8	29.3	1.4	4360	4848	17	67
LF1in	4/16/2018	7.0	35.5	2.2	1818	2578	6	47
LF1in	5/14/2018	7.4	41.6	2.4	1	709	3	36
LF1in	6/11/2018	7.3	43.4	2.3	3	2309	29	577
LF1in	11/5/2018	7.4	56.4	2.8	17	742	15	44
LF1in	12/10/2018	6.8	33.9	1.1	6341	7473	19	90

LF1in	1/14/2019	6.8	36.9	1.5	3467	4148	15	31
LF1in	3/5/2019	7.0	37.0	1.6	2158	2857	4	20
LF1in	3/18/2019	6.8	27.5	0.8	3782	4869	48	128
LF1in	4/24/2019	7.7	54.9	3.1	20	701	2	42
LF1in	5/21/2019	7.3	58.5	3.4	2	941	5	67
LF1in	8/12/2019	7.1	34.6	1.8	3	988	31	100
LF1in	9/17/2019	7.2	48.8	2.6	0	1288	17	115
LF1in	11/11/2019	7.1	44.5	2.5	2229	2431	9	44
LF1in	12/9/2019	7.0	32.4	1.4	4991	5115	26	115
LF1in	1/13/2020	7.0	46.8	2.6	2653	3545	8	23
LF1in	2/11/2020	7.2	43.9	2.5	2378	3310	14	33
LF1in	3/9/2020	7.3	31.8	1.5	4048	5409	26	78
LF1in	4/6/2020	7.7	45.9	2.9	1531	2521	4	30
LF1in	5/4/2020	7.4	41.6	2.2	6210	7155	10	61
LF1in	5/18/2020	7.8	40.1	2.8	14	1205	2	56
LF1in	6/8/2020	7.5	52.7	3.6	0	1401	0	74
LF1in	7/6/2020	7.5	60.5	4.7	4	1458	4	73
LF1in	12/9/2020	7.2		3.1	798	1941	78	145
LF1in	1/11/2021		37.2		1112	1828	47	82
LF1in	1/26/2021		31.5		2599	3165	41	75
LF1in	2/8/2021		44.5		612	1688	68	120
LF1in	2/22/2021		50.4		1408	1843	71	86
LF1in	3/9/2021		42.9		1589	2061	13	34
LF1in	3/29/2021		44.2		520	942	5	26
LF1in	4/19/2021		53.5		0	681	1	44
LF1in	5/17/2021		38.0		2	956	4	96
LF1in	6/7/2021		38.9		13	940	7	63
LF1in	10/11/2021		56.7		1	875	22	66
LF1in	11/10/2021		55.1		2357	3486	20	118
LF1in	11/22/2021		55.0		2323	4024	8	78
LF1in	12/6/2021		79.7		4	1659	62	135
LF1in	12/14/2021		41.6		1564	3320	139	223
LF1in	1/17/2022		27.4		1910	3024	23	95
LF1in	1/31/2022		15.3		1764	3329	135	308
LF1in	2/14/2022		20.6		356	1135	35	132
LF1in	2/28/2022		19.8		880	1640	21	70
LF1in	3/15/2022		39.5		382	1035	13	54
LF1out	11/4/2013	9.0	54.1	2.0	2121	2993	12	39
LF1out	11/12/2013	7.7	46.4	1.9	4839	5453	21	58
LF1out	12/11/2013	7.8	31.9	2.1	2457	3071	15	42
LF1out	1/14/2014	8.0	45.2	2.4	1828	3924	23	52
LF1out	2/11/2014	7.5	33.7	1.7	3192	3863	22	65
LF1out	3/3/2014	7.9	43.0	2.1	1945	2723	8	23
LF1out	3/10/2014	8.4	42.7	2.0	1456	2047	3	21
LF1out	4/1/2014	9.9	33.8	1.1	985	2280	3	58
LF1out	4/14/2014	9.8	37.9	1.0	7	2190	4	100
LF1out	5/13/2014	8.9	51.2	2.5	45	780	1	30
LF1out	6/10/2014	8.7	51.0	3.4	1	1968	1	150
LF1out	10/13/2014	7.4	53.2	1.9	2615	4331	14	58
LF1out	11/10/2014	7.8	52.9	2.9	1904	2852	6	21

LF1out	12/8/2014	7.6	59.4	3.3	2059	2310	3	13
LF1out	1/19/2015	7.6	35.5	2.2	1161	1749	13	33
LF1out	2/3/2015	7.2	41.4	2.1	2208	3041	19	60
LF1out	2/16/2015	7.4	42.6	2.6	1453	1806	4	16
LF1out	2/23/2015	7.3	42.0	2.0	3048	3206	6	42
LF1out	3/3/2015	7.5	43.0	2.4	2041	2333	4	17
LF1out	3/16/2015	7.5	43.2	2.6	1576	2204	19	43
LF1out	4/14/2015	8.0	42.0	2.6	610	1348	4	24
LF1out	5/18/2015	7.7	48.8	2.8	5	704	2	31
LF1out	5/25/2015	8.0	34.6	2.4	0	913	3	48
LF1out	6/9/2015	8.1	34.9	2.5	2	752	1	29
LF1out	7/14/2015	7.5	47.9	3.7	6	808	0	23
LF1out	8/18/2015	7.6	58.2	3.8	3	665	2	15
LF1out	9/9/2015	7.7		3.5	0	534	2	17
LF1out	9/15/2015	7.5	51.7	3.3	5	603	0	36
LF1out	10/19/2015	7.3	57.3	4.1	3	395	1	6
LF1out	11/9/2015	7.6	57.7	4.1	2	377	6	33
LF1out	12/14/2015	7.3	43.2	2.9	872	1751	11	31
LF1out	1/18/2016	6.9	51.0	3.3	1031	1941	9	32
LF1out	1/28/2016	7.1	41.1	2.0	1445	1874	18	58
LF1out	2/15/2016	6.9	40.3	2.5	1061	1732	13	39
LF1out	3/14/2016	7.1	36.4	2.4	546	1276	14	54
LF1out	4/11/2016	7.4	47.5	3.4	686	1307	3	19
LF1out	5/16/2016	7.6	45.7	3.3	0	697	3	30
LF1out	6/20/2016	7.7	50.8	3.7	9	3337	1	213
LF1out	7/19/2016	7.6		4.2	0	1187	1	73
LF1out	9/13/2016	7.4	57.1	4.5	8	1073	5	40
LF1out	10/10/2016	7.5	60.9	4.1	8	871	2	21
LF1out	11/7/2016	7.4	57.0	2.3	11899	13885	7	41
LF1out	11/15/2016	7.1	34.9	1.2	5498	7764	35	122
LF1out	11/22/2016	6.8	30.6	1.3	4824	6926	23	108
LF1out	12/12/2016	7.2	44.6	2.4	3981	4016	4	16
LF1out	1/10/2017	7.1	50.8	3.0	3600	4002	0	15
LF1out	1/30/2017	7.1	42.2	2.2	3865	4877	4	26
LF1out	2/13/2017	7.2	56.1	3.2	3724	4161	1	15
LF1out	3/14/2017	7.1	33.8	1.7	2984	3642	17	71
LF1out	3/20/2017	7.3	42.3	2.4	3912	5567	46	142
LF1out	4/10/2017	7.7	42.6	2.7	1608	2470	5	36
LF1out	5/15/2017	7.6	45.5	2.7	2	686	6	38
LF1out	6/12/2017	7.2	53.3	3.9	3	1195	19	87
LF1out	9/12/2017	7.2	46.6	2.9	3	1242	11	57
LF1out	10/16/2017	7.1	32.4	1.5	3020	4238	15	57
LF1out	11/15/2017	7.0	36.4	1.8	2411	3722	30	70
LF1out	12/11/2017	7.0	33.7	1.7	3066	4577	46	106
LF1out	1/15/2018	7.0	39.1	2.2	4573	4777	9	28
LF1out	2/12/2018	7.0	38.0	2.0	2977	3966	9	24
LF1out	3/19/2018	6.9	34.0	1.8	2038	2618	8	23
LF1out	3/27/2018	6.9	30.5	1.4	3972	4480	16	61
LF1out	4/16/2018	7.2	37.1	2.2	2106	2708	5	31
LF1out	5/14/2018	7.4	42.8	2.6	6	556	16	40

LF1out	6/11/2018	7.4	49.3	3.1	100	1074	25	58
LF1out	11/5/2018	7.5	59.6	3.1	17	1005	15	38
LF1out	12/10/2018	6.9	35.3	1.2	6609	7083	18	85
LF1out	1/14/2019	7.0	39.5	1.7	3093	3834	9	31
LF1out	3/5/2019	7.1	37.7	1.7	2130	2901	4	18
LF1out	4/24/2019	7.5	55.6	3.2	7	455	2	20
LF1out	5/21/2019	7.4	61.4	3.8	4	766	14	56
LF1out	8/12/2019	7.1	87.1	7.1	6	1528	21	80
LF1out	9/17/2019	7.2	56.0	3.4	2	655	4	38
LF1out	11/11/2019	7.0	45.7	2.6	2310	2452	6	27
LF1out	12/9/2019	7.0	32.6	1.5	3921	5344	27	117
LF1out	1/13/2020	7.2	44.6	2.5	2972	3752	6	18
LF1out	2/11/2020	7.2	43.5	2.5	2370	3233	14	33
LF1out	3/9/2020	7.3	32.2	1.6	4297	4885	27	81
LF1out	4/6/2020	7.6	45.5	2.8	1311	2294	2	35
LF1out	5/4/2020	7.5	41.8	2.2	5452	6256	11	62
LF1out	5/18/2020	7.6	42.5	2.8	423	1237	4	33
LF1out	6/8/2020	7.3	55.5	4.2	1	945	22	63
LF1out	7/6/2020	7.4	67.4	5.6	115	1188	13	58
LF1out	12/9/2020	7.3		3.5	19	1083	27	77
LF1out	1/11/2021		34.8		1521	2078	21	50
LF1out	1/26/2021		31.8		2463	2725	32	58
LF1out	2/8/2021		43.3		1688	2212	16	27
LF1out	2/22/2021		49.1		1421	1874	23	38
LF1out	3/9/2021		43.1		1779	2206	8	25
LF1out	3/29/2021		44.3		562	965	5	24
LF1out	4/19/2021		53.1		5	444	4	21
LF1out	5/17/2021		40.2		4	697	9	43
LF1out	6/7/2021		42.2		8	571	16	40
LF1out	10/11/2021		59.0		9	715	6	33
LF1out	11/10/2021		58.7		1912	2826	17	89
LF1out	11/22/2021		55.6		192	1061	8	74
LF1out	12/6/2021		77.5		1	994	25	57
LF1out	12/14/2021		38.1		1664	3565	65	298
LF1out	1/17/2022		28.3		1634	2417	19	49
LF1out	2/14/2022		33.3		881	1410	18	52
LF1out	3/15/2022		36.9		1084	1866	12	62
LF2in	11/4/2013	6.9	24.4	0.4	594	4164	66	421
LF2in	11/12/2013	6.6	31.7	0.4	5275	6349	136	355
LF2in	12/11/2013	6.5	22.3	0.4	3243	4703	203	428
LF2in	1/14/2014	6.3	16.0	0.4	2136	3684	159	401
LF2in	2/11/2014	6.4	18.9	0.5	2322	3800	119	336
LF2in	3/3/2014	7.0	19.3	0.6	1610	2934	129	271
LF2in	3/10/2014	7.3	20.4	0.6	1338	2542	100	237
LF2in	4/1/2014	7.0	20.1	0.6	1940	3289	17	239
LF2in	4/14/2014	7.3	20.0	0.6	1840	3333	84	254
LF2in	5/13/2014	8.1	22.4	0.8	11	1466	19	148
LF2in	6/10/2014	7.2	27.7	1.2	0	2329	29	175
LF2in	10/13/2014	6.3	30.1	0.3	3038	5080	150	364
LF2in	11/10/2014	6.4	42.9	0.5	2717	4184	79	176

LF2in	12/8/2014	6.7	42.3	0.7	1208	2497	56	109
LF2in	1/19/2015	6.3	23.5	0.4	4639	5728	73	170
LF2in	2/3/2015	6.3	17.3	0.4	3607	4658	91	204
LF2in	2/16/2015	6.5	23.2	0.5	2071	4076	48	294
LF2in	2/23/2015	6.4	23.2	0.4	2675	4009	23	181
LF2in	3/3/2015	6.7	27.8	0.6	3046	4459	79	233
LF2in	3/16/2015	7.3	23.9	0.7	1547	3661	24	188
LF2in	4/14/2015	7.0	24.9	0.7	3525	5138	19	155
LF2in	5/18/2015	7.0	14.4	0.5	7	1339	6	82
LF2in	5/25/2015	7.0	19.5	0.6	2305	3967	16	205
LF2in	6/9/2015	6.8	14.9	0.6	416	2426	16	167
LF2in	7/14/2015	6.7	19.3	1.2	0	1964	117	367
LF2in	9/9/2015	6.7		0.9	550	2313	125	272
LF2in	9/15/2015	6.9	22.0	0.8	0	1476	57	171
LF2in	11/9/2015	5.8	43.2	0.6	2	2168	1215	1321
LF2in	12/14/2015	6.7	19.9	0.8	3	1846	92	312
LF2in	1/18/2016	6.3	23.6	0.8	899	2982	184	334
LF2in	1/28/2016	6.5	21.2	1.0	158	2640	372	456
LF2in	2/15/2016	6.3	20.5	0.7	2225	3498	146	295
LF2in	3/14/2016	6.4	20.9	0.7	1139	2394	57	172
LF2in	4/11/2016	6.9	21.6	0.9	4	1376	25	211
LF2in	5/16/2016	7.0	24.0	1.2	0	1377	42	146
LF2in	6/20/2016	6.9	24.8	0.8	390	2126	101	212
LF2in	11/7/2016	6.5	34.3	1.0	2583	5845	31	494
LF2in	11/15/2016	6.3	22.3	0.3	6369	8484	88	218
LF2in	11/22/2016	6.2	21.0	0.4	6706	9033	99	331
LF2in	12/12/2016	6.6	25.7	1.1	1	1776	364	512
LF2in	1/10/2017	6.6	28.2	1.2	2	2072	183	289
LF2in	1/30/2017	6.6	25.9	0.7	4714	6534	82	225
LF2in	2/13/2017	6.5	26.2	1.2	45	2398	141	402
LF2in	3/14/2017	6.4	15.3	0.4	1426	2295	17	108
LF2in	3/20/2017	6.6	20.5	0.8	262	1779	58	226
LF2in	4/10/2017	6.9	25.0	1.2	19	1196	41	122
LF2in	5/15/2017	7.2	28.7	1.5	4	1325	27	108
LF2in	6/12/2017	7.3	32.2	2.2	4	1593	245	332
LF2in	9/12/2017	6.9	33.0	2.0	2	3222	791	1090
LF2in	10/16/2017	6.5	21.6	0.6	1856	3890	145	309
LF2in	11/15/2017	6.5	21.8	0.7	342	1983	197	292
LF2in	12/11/2017	6.4	19.6	0.5	1387	2737	112	219
LF2in	1/15/2018	6.4	18.1	0.6	1731	2430	116	228
LF2in	2/12/2018	6.5	17.7	0.5	1531	2492	126	256
LF2in	3/19/2018	6.4	17.3	0.5	1199	2246	85	158
LF2in	3/27/2018	6.4	20.6	0.5	1768	2675	123	218
LF2in	4/16/2018	6.7	18.1	0.7	290	1281	66	183
LF2in	5/14/2018	7.1	20.9	0.9	1	1596	97	255
LF2in	11/5/2018	6.5	45.0	0.6	244	2049	20	224
LF2in	12/10/2018	6.3	42.8	0.3	8674	10271	85	214
LF2in	1/14/2019	6.2	33.2	0.4	3253	4512	43	88
LF2in	2/11/2019	6.1	22.8	0.2	7434	8214	66	133
LF2in	3/5/2019	6.6	23.0	0.5	2326	3661	83	161

LF2in	3/18/2019	6.2	21.9	0.2	6735	7985	100	261
LF2in	4/24/2019	7.2	38.3	1.0	0	2400	24	337
LF2in	8/12/2019	6.8	16.9	0.6	77	2030	48	448
LF2in	9/17/2019	6.8	35.8	1.1	0	2252	29	429
LF2in	11/11/2019	6.5	21.3	0.6	1704	2906	118	210
LF2in	12/9/2019	6.4	19.5	0.5	3542	4893	127	300
LF2in	1/13/2020	6.7	17.3	0.6	1140	2326	123	198
LF2in	2/11/2020	6.5	14.4	0.4	1395	1933	52	109
LF2in	3/9/2020	6.7	11.3	0.3	1289	2331	44	216
LF2in	4/6/2020	7.0	16.9	0.8	0	1595	44	225
LF2in	5/4/2020	6.7	15.5	0.5	905	2044	50	189
LF2in	5/18/2020	7.0	16.8	0.9	4	1220	63	178
LF2in	6/8/2020	7.3	19.9	1.0	2	1421	60	215
LF2in	12/9/2020	7.0		1.9	11	2787	625	958
LF2in	1/11/2021		25.3		2340	3257	51	98
LF2in	1/26/2021		21.5		2696	3966	51	105
LF2in	2/8/2021		28.0		767	1731	30	86
LF2in	2/22/2021		19.3		1360	2503	179	250
LF2in	3/9/2021		26.2		1753	2451	62	106
LF2in	3/29/2021		26.2		1	1187	130	288
LF2in	4/19/2021		33.3		2	1423	46	225
LF2in	5/17/2021		28.2		69	1327	70	176
LF2in	6/7/2021		23.6		280	1499	36	143
LF2in	10/11/2021		28.8		3	1710	240	349
LF2in	11/10/2021		33.1		1551	3107	665	792
LF2in	11/22/2021		30.3		48	2265	255	680
LF2in	12/6/2021		35.2		2	1878	549	771
LF2in	12/14/2021		16.8		885	2074	43	127
LF2in	1/17/2022		23.9		1584	2720	46	114
LF2in	1/31/2022		23.1		5432	6741	33	133
LF2in	2/14/2022		24.4		137	1069	65	150
LF2in	2/28/2022		17.2		2884	3968	35	137
LF2in	3/15/2022		25.0		0	1282	232	385
LF2out	11/12/2013	6.9	76.0	3.2	526	7599	205	385
LF2out	12/11/2013	7.1	26.7	0.8	3195	4752	189	384
LF2out	1/14/2014	6.7	20.2	0.8	2227	3764	146	366
LF2out	2/11/2014	6.7	20.8	0.6	2224	3444	106	284
LF2out	3/3/2014	7.2	21.4	0.7	1839	2978	118	252
LF2out	3/10/2014	7.4	21.9	0.8	1275	2562	98	224
LF2out	4/1/2014	7.3	21.7	0.8	2180	3049	17	199
LF2out	4/14/2014	7.5	21.9	0.8	2040	3251	70	213
LF2out	5/13/2014	9.2	25.0	1.1	79	1509	19	126
LF2out	6/10/2014	8.0	38.5	2.2	2	2970	82	212
LF2out	10/13/2014	6.8	35.8	0.9	2837	4729	47	149
LF2out	11/10/2014	6.9	47.2	0.9	2597	4219	39	149
LF2out	12/8/2014	6.9	46.3	0.9	1228	2219	34	76
LF2out	1/19/2015	6.9	29.5	0.8	4045	4953	35	88
LF2out	2/3/2015	6.6	22.0	0.7	4117	4375	45	134
LF2out	2/16/2015	6.7	26.8	0.7	2295	3436	52	129
LF2out	2/23/2015	6.6	30.4	0.8	4883	5556	34	125

LF2out	3/3/2015	6.6	30.5	0.8	3079	4121	50	132
LF2out	3/16/2015	7.3	25.4	0.8	1672	2892	31	111
LF2out	4/14/2015	7.2	27.0	1.0	3704	5108	15	165
LF2out	5/18/2015	7.1	25.9	1.0	51	1271	13	60
LF2out	5/25/2015	6.9	20.8	0.7	853	3943	21	150
LF2out	6/9/2015	6.9	16.6	0.7	352	1726	15	101
LF2out	7/14/2015	8.9	22.8	1.7	1	1470	9	73
LF2out	9/9/2015	7.4		1.6	49	1522	31	93
LF2out	9/15/2015	7.4	28.4	1.6	5	1317	19	81
LF2out	11/9/2015	6.8	45.2	1.6	35	2253	110	156
LF2out	12/14/2015	6.7	26.4	1.3	8	2206	171	344
LF2out	1/18/2016	6.5	28.1	1.2	30	2178	199	272
LF2out	1/28/2016	6.7	27.1	1.6	111	2706	140	192
LF2out	2/15/2016	6.5	33.9	1.8	10	2787	209	257
LF2out	3/14/2016	6.6	30.2	1.7	18	2499	135	185
LF2out	4/11/2016	9.7	26.4	1.2	3	1492	80	191
LF2out	5/16/2016	7.3	27.2	1.5	3	1242	83	116
LF2out	6/20/2016	7.8	28.4	1.1	13	1825	42	172
LF2out	11/7/2016	6.9	40.6	1.5	271	2761	26	268
LF2out	11/15/2016	6.8	32.5	1.3	42	1675	59	195
LF2out	11/22/2016	6.6	31.1	1.0	1771	3922	63	226
LF2out	12/12/2016	6.7	29.8	1.7	1	1859	105	230
LF2out	1/10/2017	6.7	29.7	1.5	7	1806	139	249
LF2out	1/30/2017	6.7	28.4	1.7	7	2082	119	228
LF2out	2/13/2017	6.7	32.6	2.2	8	2354	147	250
LF2out	3/14/2017	6.5	27.1	1.4	39	2338	135	258
LF2out	3/20/2017	6.7	25.2	1.6	1	2046	152	282
LF2out	4/10/2017	6.8	28.5	1.6	8	1825	107	186
LF2out	5/15/2017	7.1	28.8	1.5	43	1900	60	225
LF2out	6/12/2017	7.3	34.1	2.5	24	1730	76	216
LF2out	9/12/2017	7.0	33.5	2.0	4	2999	738	1094
LF2out	10/16/2017	6.5	21.7	0.6	1661	3743	142	305
LF2out	11/15/2017	6.6	23.4	1.1	9	3004	189	852
LF2out	12/11/2017	6.6	21.5	1.0	74	1731	104	270
LF2out	1/15/2018	6.6	25.3	1.2	101	3040	186	276
LF2out	2/12/2018	6.6	23.2	1.0	185	1762	118	311
LF2out	3/19/2018	6.5	24.5	1.1	233	1825	121	280
LF2out	3/27/2018	6.5	16.1	1.0	410	2982	98	159
LF2out	4/16/2018	6.6	19.7	0.7	255	1463	73	215
LF2out	5/14/2018	7.1	23.1	1.2	6	1490	99	216
LF2out	11/5/2018	6.9	62.8	1.6	90	3263	69	744
LF2out	12/10/2018	6.2	44.5	0.4	8540	8960	83	219
LF2out	1/14/2019	6.2	34.8	0.5	2982	4373	70	120
LF2out	2/11/2019	6.1	19.0	0.2	4503	5894	56	113
LF2out	3/5/2019	6.5	27.3	0.6	2718	3846	64	128
LF2out	3/18/2019	6.3	23.7	0.3	5075	7427	81	486
LF2out	4/24/2019	7.1	14.9	1.4	18	1579	41	162
LF2out	8/12/2019	7.0	22.2	1.2	42	1821	63	356
LF2out	9/17/2019	7.2	40.3	2.1	4	1491	74	167
LF2out	11/11/2019	6.5	23.3	0.8	1492	2856	155	250

LF2out	12/9/2019	6.4	19.7	0.5	3297	4566	119	286
LF2out	1/13/2020	6.6	17.9	0.7	1032	2415	159	328
LF2out	2/11/2020	6.5	20.1	0.7	1379	2478	101	194
LF2out	3/9/2020	6.6	14.1	0.5	1342	2573	89	263
LF2out	4/6/2020	7.0	18.4	0.8	46	1280	77	203
LF2out	5/4/2020	6.8	17.6	0.7	481	1776	63	196
LF2out	5/18/2020	7.1	19.1	1.0	3	1399	72	191
LF2out	6/8/2020	7.0	25.3	1.7	7	2424	189	967
LF2out	12/9/2020	6.9		1.9	269	2727	448	815
LF2out	1/11/2021		26.6		2238	3364	70	121
LF2out	1/26/2021		26.0		2845	4373	70	135
LF2out	2/8/2021		29.8		596	2286	136	280
LF2out	2/22/2021		29.4		60	1586	485	552
LF2out	3/9/2021		29.6		2	1776	230	300
LF2out	3/29/2021		25.9		1	1026	92	202
LF2out	4/19/2021		35.6		1	2144	254	358
LF2out	5/17/2021		28.8		47	1279	77	207
LF2out	6/7/2021		23.3		224	1367	51	150
LF2out	10/11/2021		35.1		6	1701	244	393
LF2out	11/10/2021		37.4		28	2091	1195	1294
LF2out	11/22/2021		25.0		54	2032	342	580
LF2out	12/6/2021		35.7		8	1842	546	685
LF2out	12/14/2021		25.3		758	2084	139	264
LF2out	1/17/2022		29.3		16	1559	197	333
LF2out	1/31/2022		29.2		412	2358	230	376
LF2out	2/14/2022		29.4		0	2251	346	448
LF2out	2/28/2022		28.1		276	2866	173	410
LF2out	3/15/2022		30.5		5	1653	288	445
LF3in	6/9/2015	7.3	14.2	0.8	0	924	64	108
LF3in	9/9/2015	6.7		0.3	34	1035	79	131
LF3in	9/15/2015	6.9	15.8	0.5	50	1446	143	272
LF3in	11/9/2015	6.9	23.6	0.4	515	2214	113	211
LF3in	12/14/2015	6.7	16.3	0.5	328	1188	44	87
LF3in	1/28/2016	6.8	12.2	0.3	537	2115	86	233
LF3in	2/15/2016	6.6	13.9	0.4	351	1144	38	89
LF3in	3/14/2016	6.9	14.9	0.5	386	1618	72	161
LF3in	4/11/2016	7.2	17.1	0.7	262	1345	109	233
LF3in	5/16/2016	7.4	22.0	0.9	359	1638	141	252
LF3in	11/7/2016	6.6	37.3	0.3	2402	4139	86	200
LF3in	11/15/2016	6.6	14.7	0.2	1466	2043	108	171
LF3in	11/22/2016	6.4	13.1	0.2	429	1320	67	123
LF3in	12/12/2016	6.7	18.6	0.3	964	1568	74	139
LF3in	1/10/2017	6.7	18.0	0.4	629	1729	116	177
LF3in	1/30/2017	6.9	15.8	0.4	584	1625	67	276
LF3in	2/13/2017	6.6	24.2	0.9	361	1850	94	206
LF3in	3/14/2017	6.9	12.7	0.3	363	1419	66	144
LF3in	3/20/2017	7.0	15.6	0.4	513	1487	73	144
LF3in	4/10/2017	7.2	19.0	0.6	504	1670	134	237
LF3in	10/16/2017	6.8	14.0	0.3	185	1290	100	169
LF3in	11/15/2017	6.8	13.9	0.4	285	1317	65	128

LF3in	12/11/2017	6.9	12.5	0.4	305	1465	61	122
LF3in	1/15/2018	7.0	13.7	0.5	303	1146	61	114
LF3in	2/12/2018	6.5	14.9	0.6	479	1561	69	128
LF3in	3/27/2018	6.8	10.2	0.3	282	1134	52	119
LF3in	4/16/2018	6.9	14.0	0.4	300	1286	69	154
LF3in	12/10/2018	6.7	17.1	0.2	1260	1891	52	133
LF3in	1/14/2019	6.8	26.5	0.3	738	2888	58	63
LF3in	2/11/2019	6.4	13.7	0.2	506	1604	61	132
LF3in	3/5/2019	6.8	17.0	0.3	493	1314	51	95
LF3in	3/18/2019	6.7	12.8	0.2	494	1354	51	104
LF3in	10/14/2019	7.1	26.3	0.6	762	2739	280	494
LF3in	11/11/2019	7.1	19.4	0.5	441	1457	103	169
LF3in	12/9/2019	6.7	93.5	0.3	437	1557	72	155
LF3in	1/13/2020	7.1	19.1	0.8	300	1462	97	377
LF3in	2/11/2020	7.0	13.8	0.4	690	2230	84	232
LF3in	3/9/2020	7.1	13.5	0.4	382	1283	67	148
LF3in	4/6/2020	7.3	19.6	0.8	70	1051	103	165
LF3in	5/4/2020	7.2	14.0	0.3	204	1178	75	164
LF3in	5/18/2020	7.6	20.1	0.8	235	1336	162	233
LF3in	12/9/2020	6.8		0.4	4999	5575	169	484
LF3in	1/11/2021		16.0		851	1622	61	121
LF3in	1/26/2021		12.7		861	1385	49	91
LF3in	2/8/2021		18.0		894	1940	93	145
LF3in	2/22/2021		18.2		1115	2661	146	262
LF3in	3/9/2021		17.3		588	1501	90	144
LF3in	3/29/2021		18.6		357	1176	113	211
LF3in	5/17/2021		21.7		464	1976	143	343
LF3in	6/7/2021		36.2		298	1412	131	226
LF3in	11/10/2021		25.3		1523	2709	125	250
LF3in	12/14/2021		13.8		621	1882	102	181
LF3in	1/17/2022		14.1		542	1359	59	108
LF3in	1/31/2022		11.3		645	1601	45	110
LF3in	2/14/2022		11.3		400	2310	161	441
LF3in	2/28/2022		11.3		403	1103	40	93
LF3in	3/15/2022		16.7		438	1391	81	130
LF3out	4/16/2013	6.6		0.3	459	1736	111	215
LF3out	4/18/2013	6.6	8.2	0.3	355	1814	142	272
LF3out	4/22/2013	6.7	9.5	0.4	276	1281	135	214
LF3out	4/29/2013	7.1	12.4	0.7	181	1153	138	243
LF3out	5/15/2013	7.3	21.6	1.7	25	1068	186	222
LF3out	6/10/2013	6.6		3.2	21	2607	1523	1749
LF3out	10/29/2013	6.3	42.6	0.2	1408	2864	200	317
LF3out	11/4/2013	6.6	34.9	0.3	732	1912	111	206
LF3out	11/12/2013	6.7	18.0	0.3	585	1416	81	134
LF3out	12/11/2013	6.8	14.0	0.3	811	1609	78	130
LF3out	1/14/2014	6.5	10.8	0.3	472	1321	72	105
LF3out	2/11/2014	6.7	10.5	0.4	690	1469	57	104
LF3out	3/3/2014	6.9	15.4	0.4	394	1389	70	152
LF3out	3/10/2014	7.1	13.4	0.5	236	989	62	126
LF3out	4/1/2014	7.1	12.3	0.5	184	967	55	108

LF3out	4/14/2014	7.1	11.8	0.5	167	1121	61	121
LF3out	5/13/2014	7.3	16.5	0.6	263	1658	91	189
LF3out	10/13/2014	6.7	14.2	0.2	344	1721	88	152
LF3out	11/10/2014	6.9	14.7	0.3	551	1521	104	157
LF3out	12/8/2014	7.1	18.3	0.5	759	1719	65	162
LF3out	1/19/2015	6.9	10.5	0.3	462	1196	46	92
LF3out	2/3/2015	6.7	8.7	0.2	466	1175	43	91
LF3out	2/16/2015	6.7	12.7	0.5	374	1207	53	82
LF3out	2/23/2015	6.9	12.6	0.4	511	1570	69	128
LF3out	3/3/2015	7.0	14.2	0.5	474	1404	61	120
LF3out	3/16/2015	7.3	14.3	0.6	246	1359	50	94
LF3out	4/14/2015	7.4	15.2	0.9	218	1077	75	122
LF3out	5/18/2015	7.0	14.4	0.4	367	1688	97	245
LF3out	5/25/2015	7.4	12.0	0.5	22	967	72	127
LF3out	6/9/2015	7.6	15.0	0.9	0	871	56	96
LF3out	9/9/2015	6.9		0.3	41	1024	80	131
LF3out	9/15/2015	7.0	16.1	0.7	59	1449	141	285
LF3out	11/9/2015	7.0	24.6	0.5	375	2188	114	212
LF3out	12/14/2015	6.8	16.4	0.6	346	1232	44	91
LF3out	1/18/2016	7.0	19.6	1.0	527	1705	80	149
LF3out	1/28/2016	6.9	12.3	0.3	579	2221	89	230
LF3out	2/15/2016	6.7	14.6	0.5	359	1166	34	72
LF3out	3/14/2016	7.0	15.6	0.6	381	1515	63	152
LF3out	4/11/2016	7.3	19.0	0.9	272	1272	105	192
LF3out	5/16/2016	7.5	24.8	1.2	442	1651	134	208
LF3out	6/20/2016	7.1	37.6	0.7	69	1251	89	162
LF3out	11/7/2016	6.8	37.7	0.3	2481	3742	83	139
LF3out	11/15/2016	6.7	15.4	0.2	1079	2076	114	179
LF3out	11/22/2016		13.8					
LF3out	12/12/2016	6.7	19.1	0.4	1006	1578	79	150
LF3out	1/10/2017	6.8	18.7	0.5	656	1727	115	186
LF3out	1/30/2017	6.9	16.3	0.4	621	1317	64	140
LF3out	2/13/2017	6.8	24.6	1.1	419	1560	83	158
LF3out	3/14/2017	6.9	13.3	0.3	418	1426	69	140
LF3out	3/20/2017	6.9	15.9	0.4	509	1452	74	145
LF3out	4/10/2017	7.2	19.9	0.7	527	1649	136	227
LF3out	10/16/2017	6.9	15.4	0.4	191	1198	88	138
LF3out	11/15/2017	6.8	15.7	0.4	305	1330	87	152
LF3out	12/11/2017	6.9	13.2	0.5	323	1427	65	126
LF3out	1/15/2018	7.0	14.8	0.6	310	986	65	111
LF3out	2/12/2018	6.7	15.5	0.7	471	1478	64	113
LF3out	3/19/2018	6.8	13.9	0.5	338	1217	59	109
LF3out	3/27/2018	6.8	10.7	0.4	313	1093	52	105
LF3out	4/16/2018	6.4	16.2	0.5	319	1284	68	135
LF3out	12/10/2018	6.7	17.0	0.2	1336	1959	53	102
LF3out	1/14/2019	6.8	27.1	0.3	734	1334	49	79
LF3out	2/11/2019	6.4	14.3	0.1	783	2038	54	92
LF3out	3/5/2019	6.8	17.5	0.3	556	1391	51	91
LF3out	3/18/2019	6.7	13.1	0.2	575	1452	54	110
LF3out	10/14/2019	7.1	26.6	0.6	784	2686	259	453

LF3out	11/11/2019	7.0	20.1	0.5	465	1433	99	151
LF3out	12/9/2019	6.8	14.4	0.4	449	1549	90	149
LF3out	1/13/2020	7.1	21.0	0.9	306	1111	74	157
LF3out	2/11/2020	6.9	14.2	0.5	776	2257	88	220
LF3out	3/9/2020	6.8	14.7	0.5	445	1308	68	151
LF3out	4/6/2020	7.2	21.9	1.0	103	1074	94	151
LF3out	5/4/2020	7.1	14.6	0.4	237	1293	76	163
LF3out	5/18/2020	7.4	22.1	0.9	261	1336	150	219
LF3out	12/9/2020	6.9		0.5	4350	4634	95	154
LF3out	1/11/2021		16.8		858	1568	60	102
LF3out	1/26/2021		13.6		820	1454	49	88
LF3out	2/8/2021		19.8		932	1794	87	132
LF3out	2/22/2021		18.2		1396	2932	139	234
LF3out	3/9/2021		19		678	1495	82	129
LF3out	3/29/2021		20.3		405	1222	122	226
LF3out	4/19/2021		33.6		780	1815	153	211
LF3out	5/17/2021		22		503	1931	138	325
LF3out	6/7/2021		36.7		14	764	75	115
LF3out	11/10/2021		25.7		1516	2667	118	209
LF3out	12/14/2021		14.1		639	2003	102	186
LF3out	1/17/2022		14.6		551	1376	57	100
LF3out	1/31/2022		11.8		689	1668	47	113
LF3out	2/14/2022		12.5		449	2191	134	327
LF3out	2/28/2022		11.7		421	1150	38	98
LF3out	3/15/2022		18.3		480	1330	77	120
LF3out	4/19/2022		17.2		580	1596	86	158
LF3out	6/7/2022		17.6		83	1057		
LF3out	11/29/2022		17.7		1990	3052	105	171

**Sub-catch**	**x**	**y**	**SWEREF99**
27	6574214	700379	
31	6573820	701002	
32	6573010	701414	
48	6571445	702583	
49	6570336	702216	
50	6571454	700888	
57	6574399	699743	
58	6574399	699743	
59	6573533	700413	
60	6573945	700128	
5702	6574509	699616	
5712	6574671	699425	
5719	6574423	699142	
CW1in	6574747	699113	
CW2in	6574372	699894	
CW3out	6574227	699924	
LF1in	6574600	699280	
LF1out	6574604	699294	
LF2in	6574338	699998	
LF2out	6574333	699973	

LF3in	6574072	699345
LF3out	6574090	699331
